# Supplementary figures and images for: The RAB27A effector SYTL5 regulates mitophagy and mitochondrial metabolism
Source: eLife. 2025 Nov 26;14:RP105541. doi: 10.7554/eLife.105541 (PMC12656530; doi:10.7554/eLife.105541)

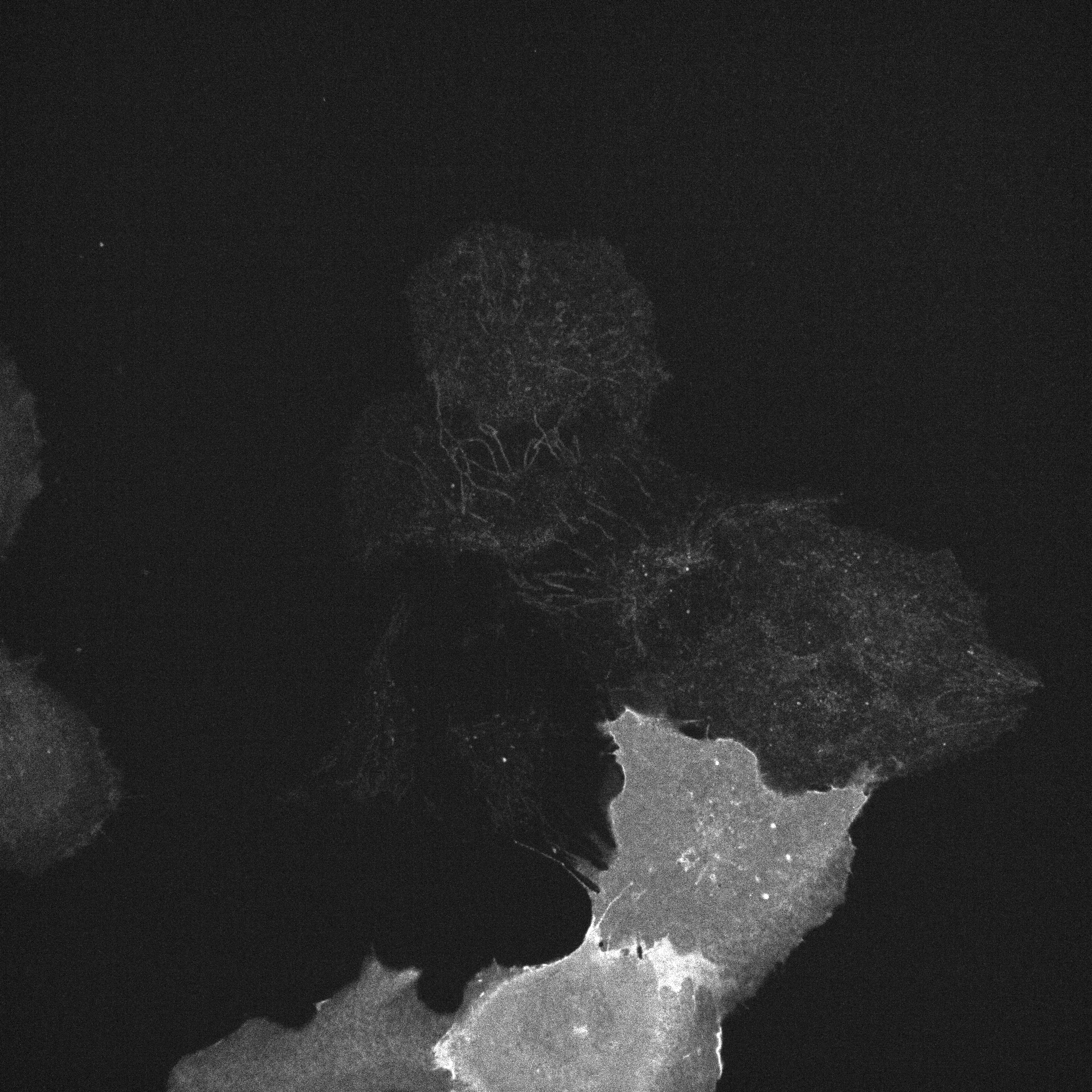

Supplement: Figure 1—source data 1. [file elife-105541-fig1-data1.zip › Figure 1-Source Data 1/Figure 1A-Source Data.tif]

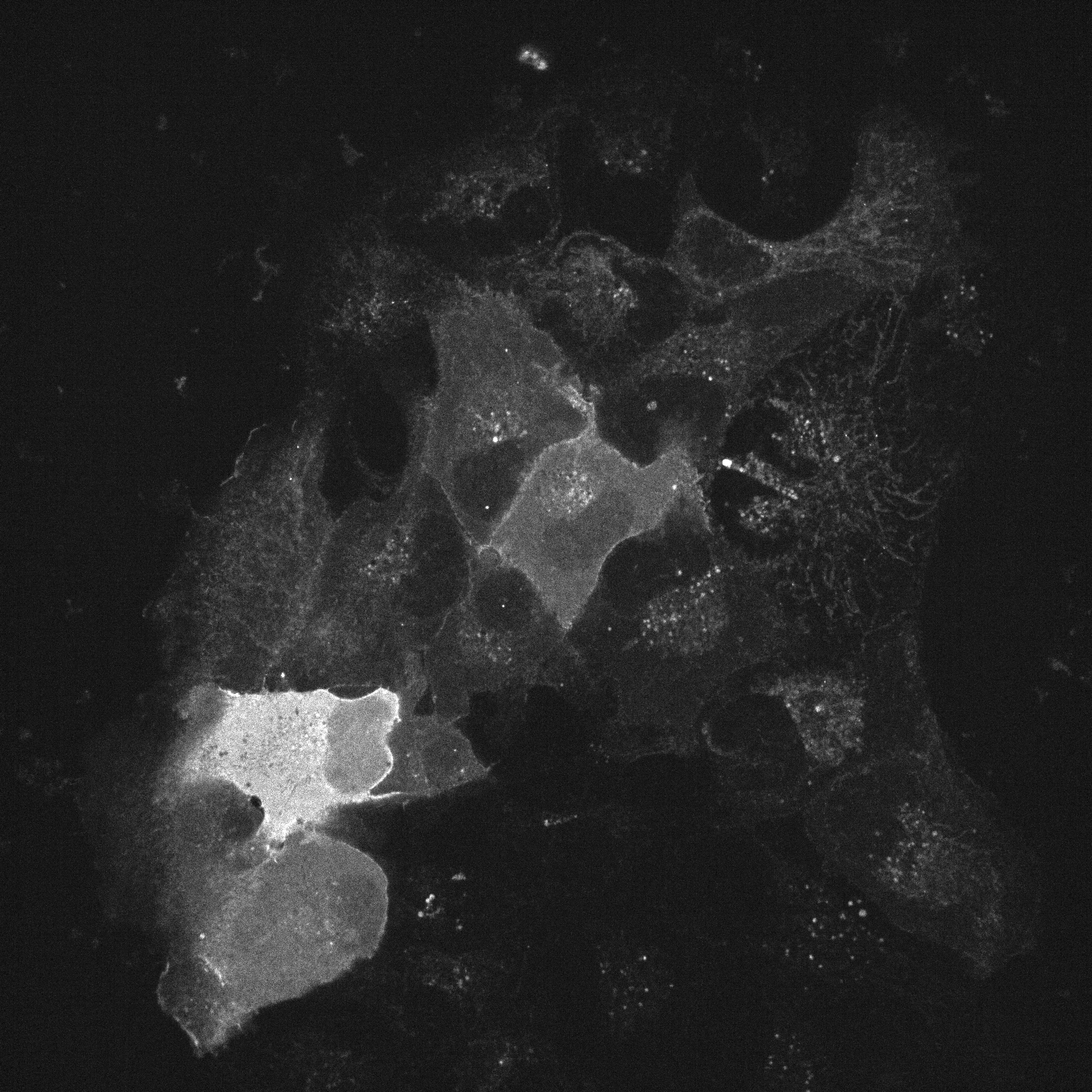

Supplement: Figure 1—source data 1. [file elife-105541-fig1-data1.zip › Figure 1-Source Data 1/Figure 1C-Source Data.tif]

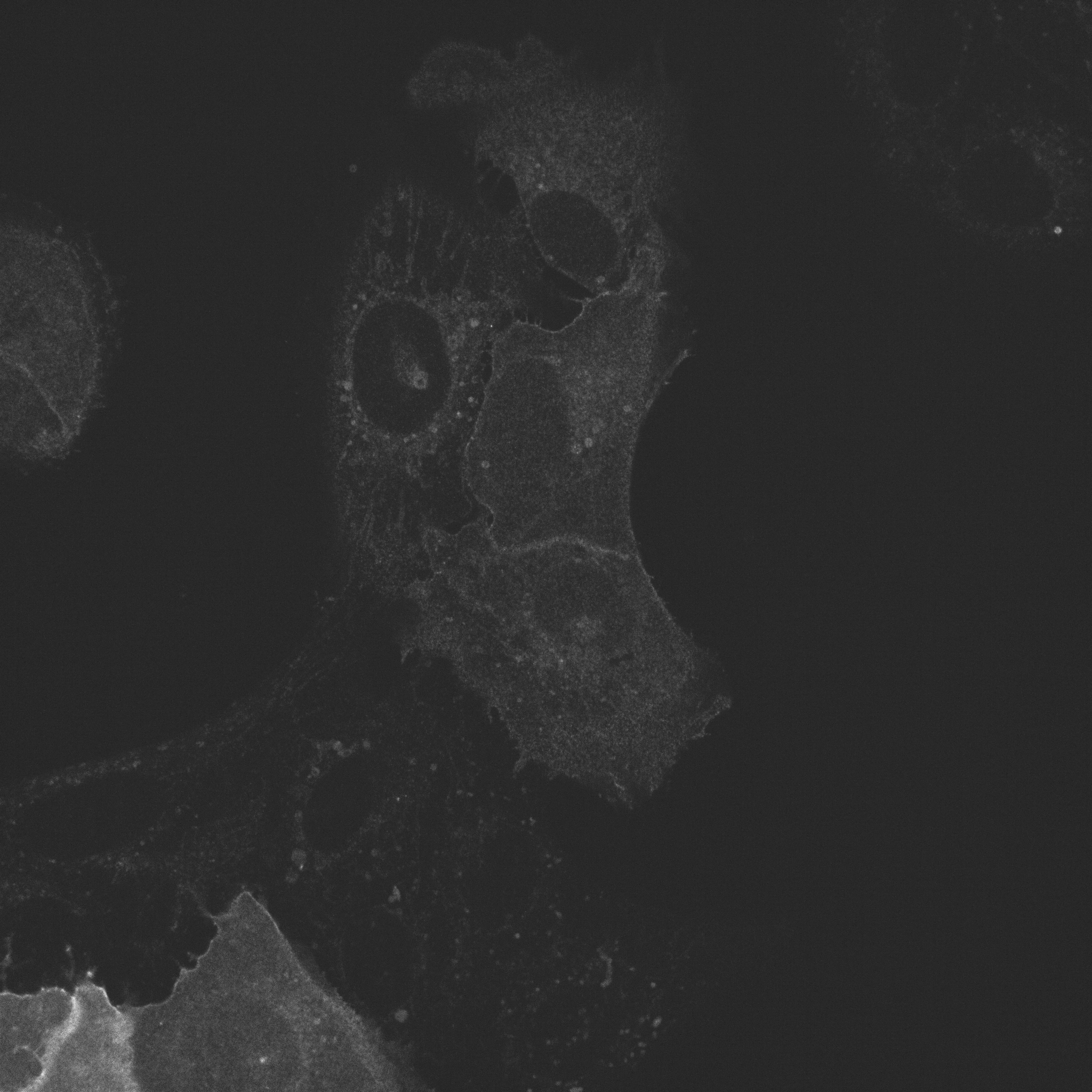

Supplement: Figure 1—source data 1. [file elife-105541-fig1-data1.zip › Figure 1-Source Data 1/Figure 1D-Source Data.tif]

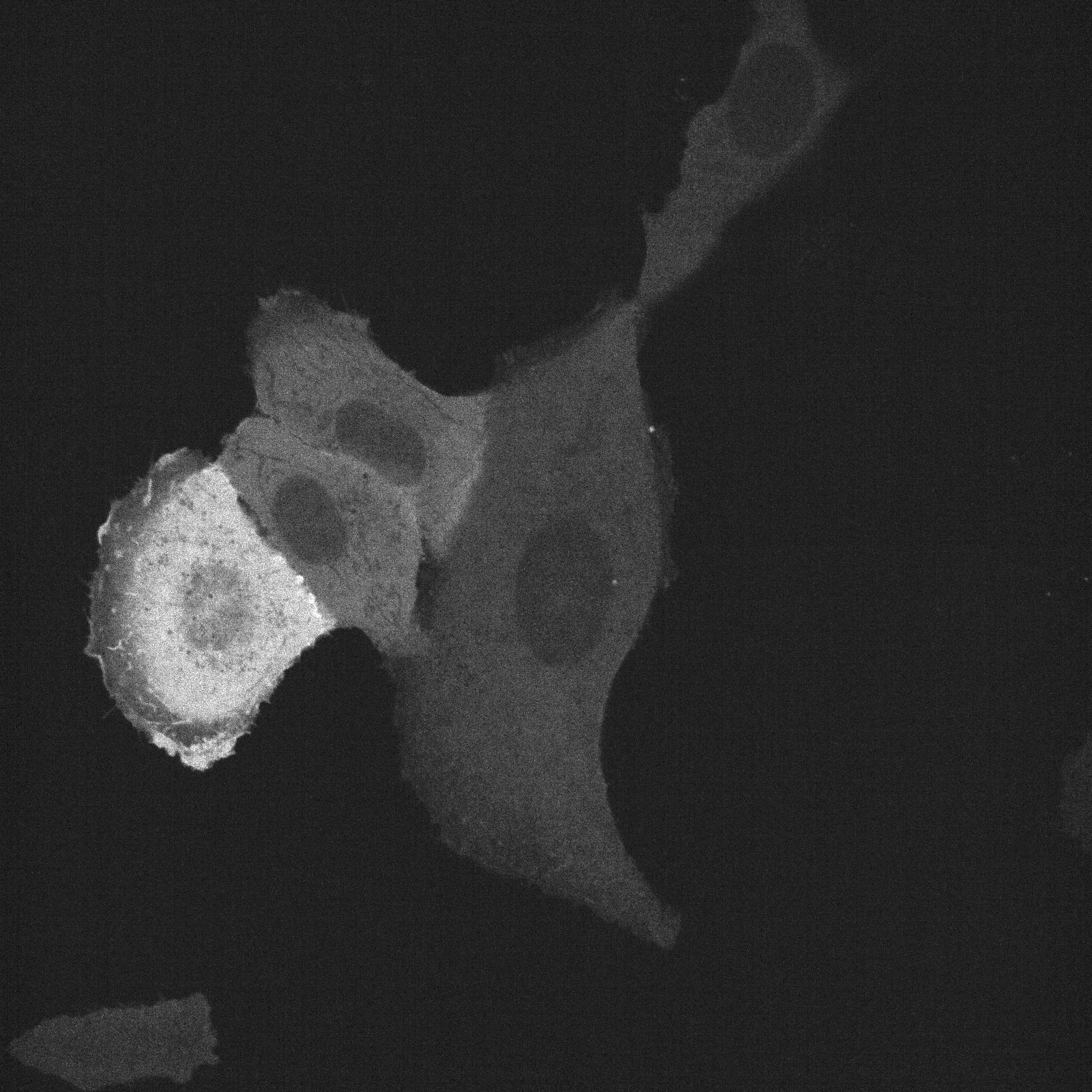

Supplement: Figure 1—source data 1. [file elife-105541-fig1-data1.zip › Figure 1-Source Data 1/Figure 1G-Source Data.tif]

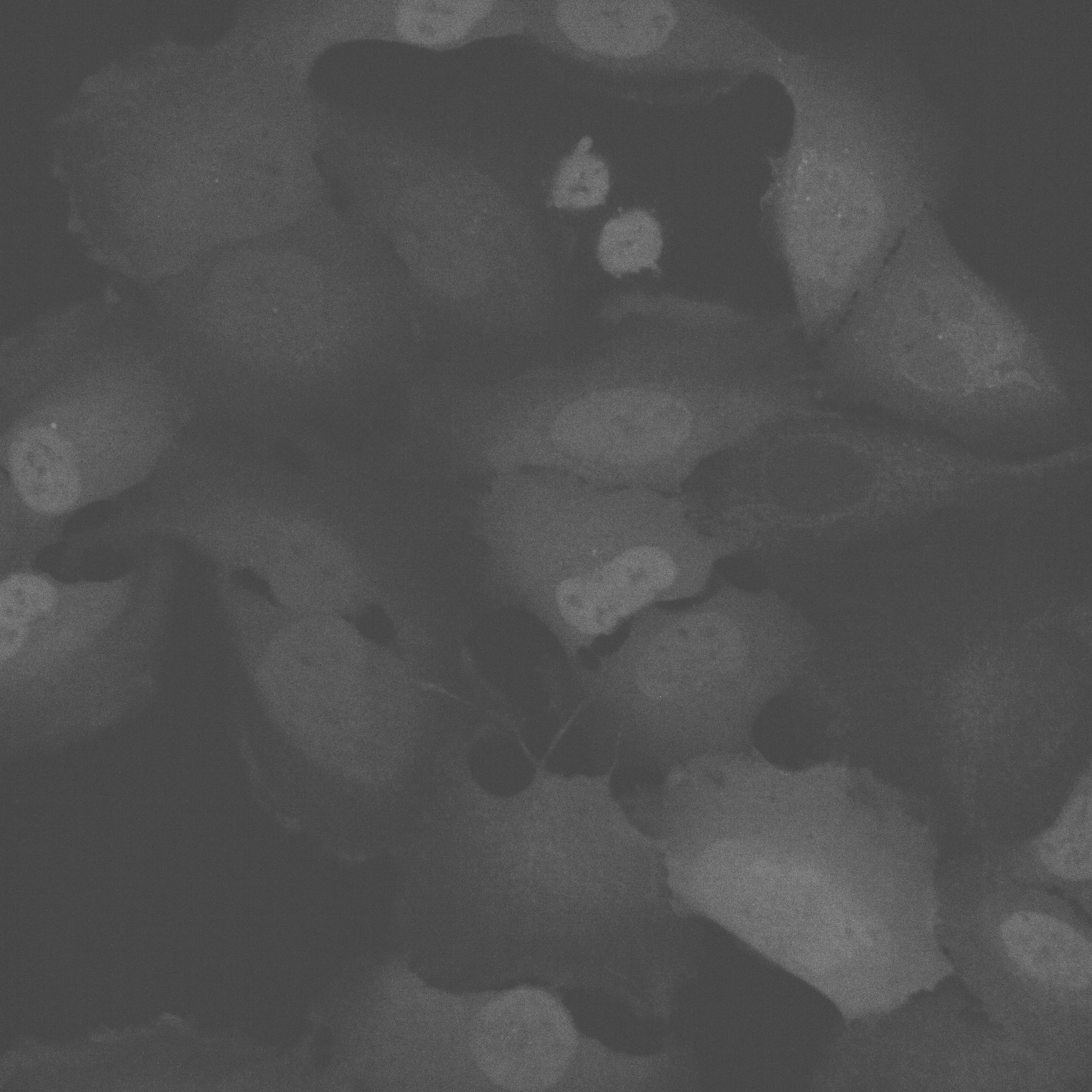

Supplement: Figure 1—source data 1. [file elife-105541-fig1-data1.zip › Figure 1-Source Data 1/Figure 1H-Source Data.tif]

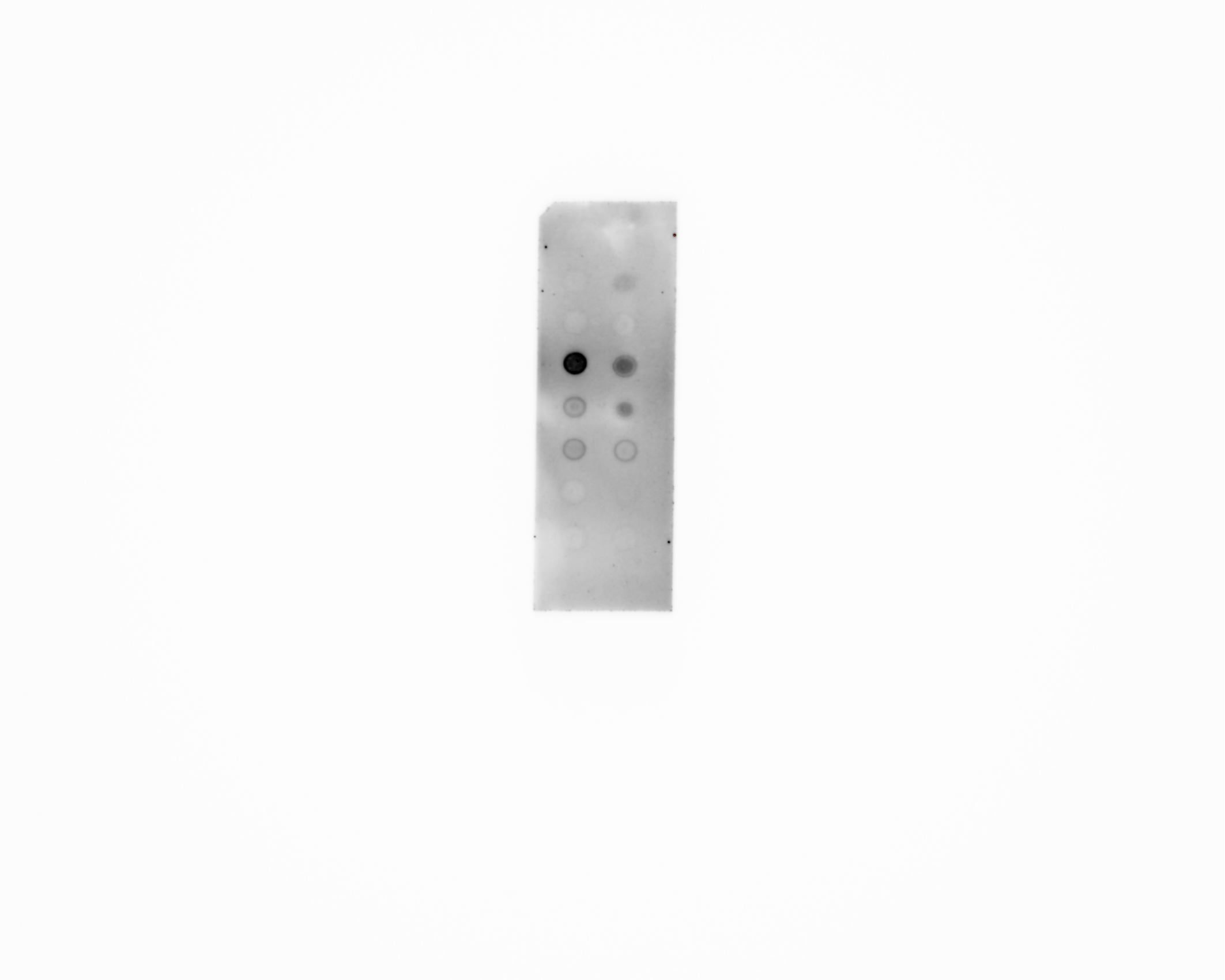

Supplement: Figure 1—source data 2. [file elife-105541-fig1-data2.zip › Figure 1-Source Data 2/Figure 1F-Source data 1.tif]

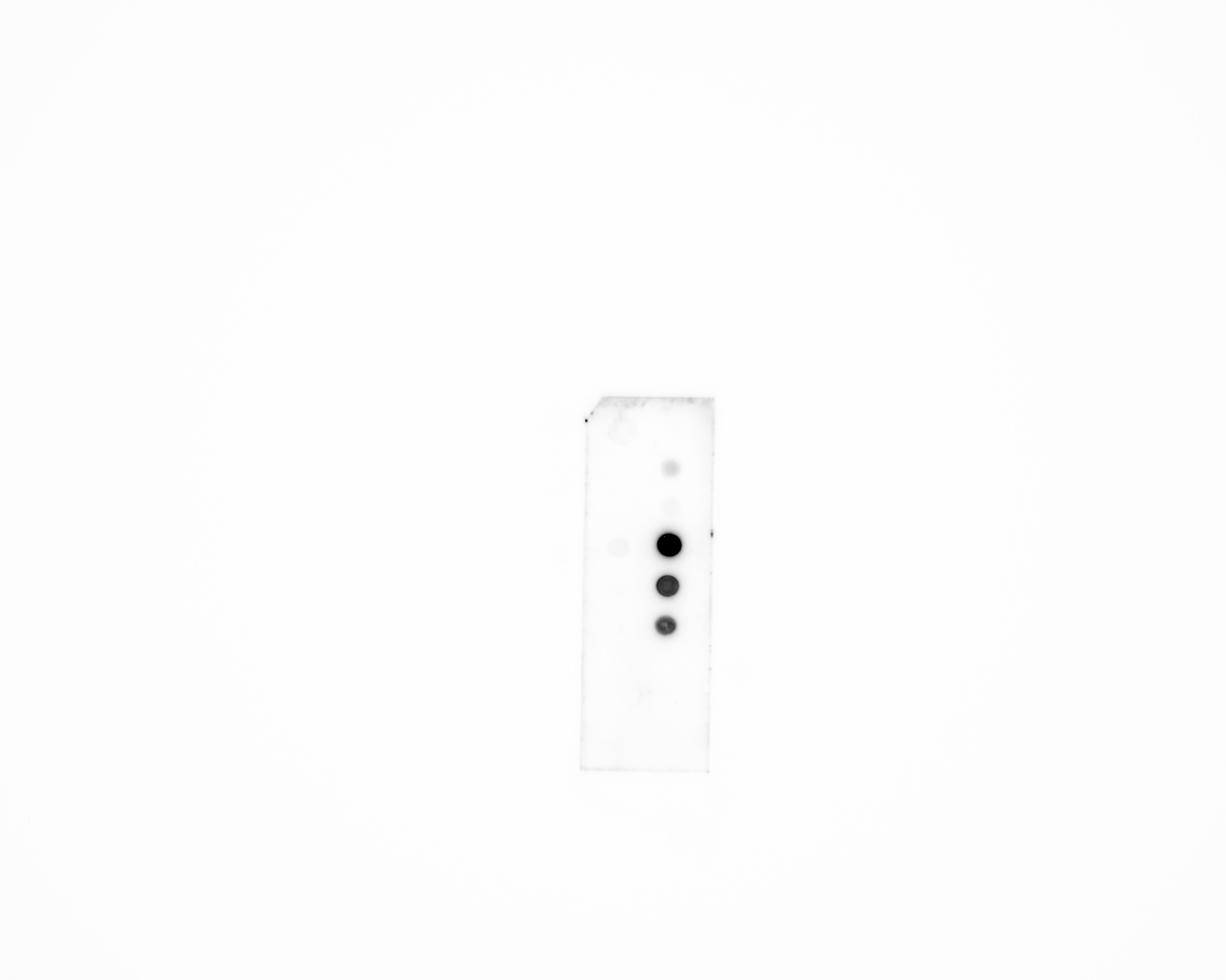

Supplement: Figure 1—source data 2. [file elife-105541-fig1-data2.zip › Figure 1-Source Data 2/Figure 1F-Source data 2.tif]

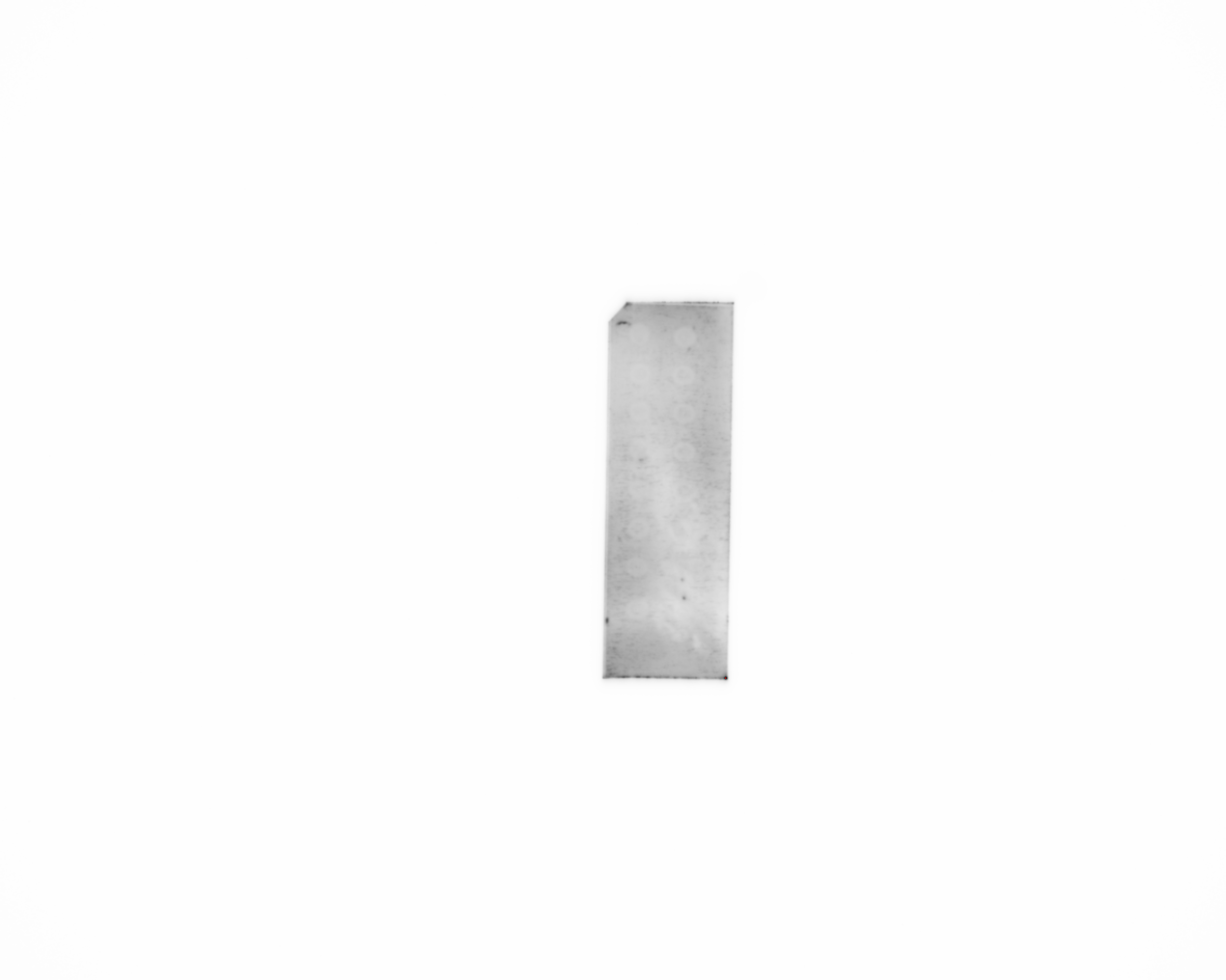

Supplement: Figure 1—source data 2. [file elife-105541-fig1-data2.zip › Figure 1-Source Data 2/Figure 1F-Source data 3.tif]

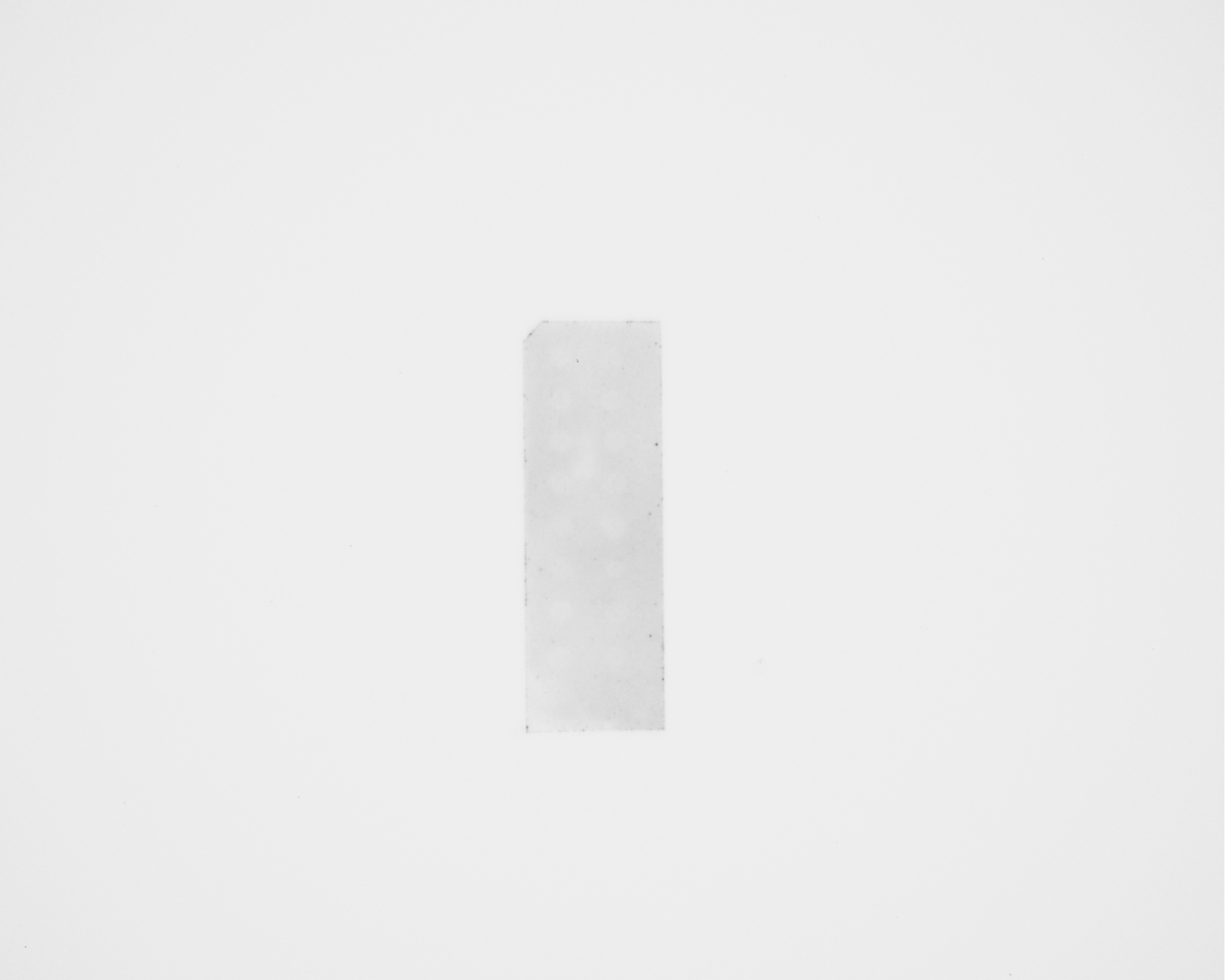

Supplement: Figure 1—source data 2. [file elife-105541-fig1-data2.zip › Figure 1-Source Data 2/Figure 1F-Source data 4.tif]

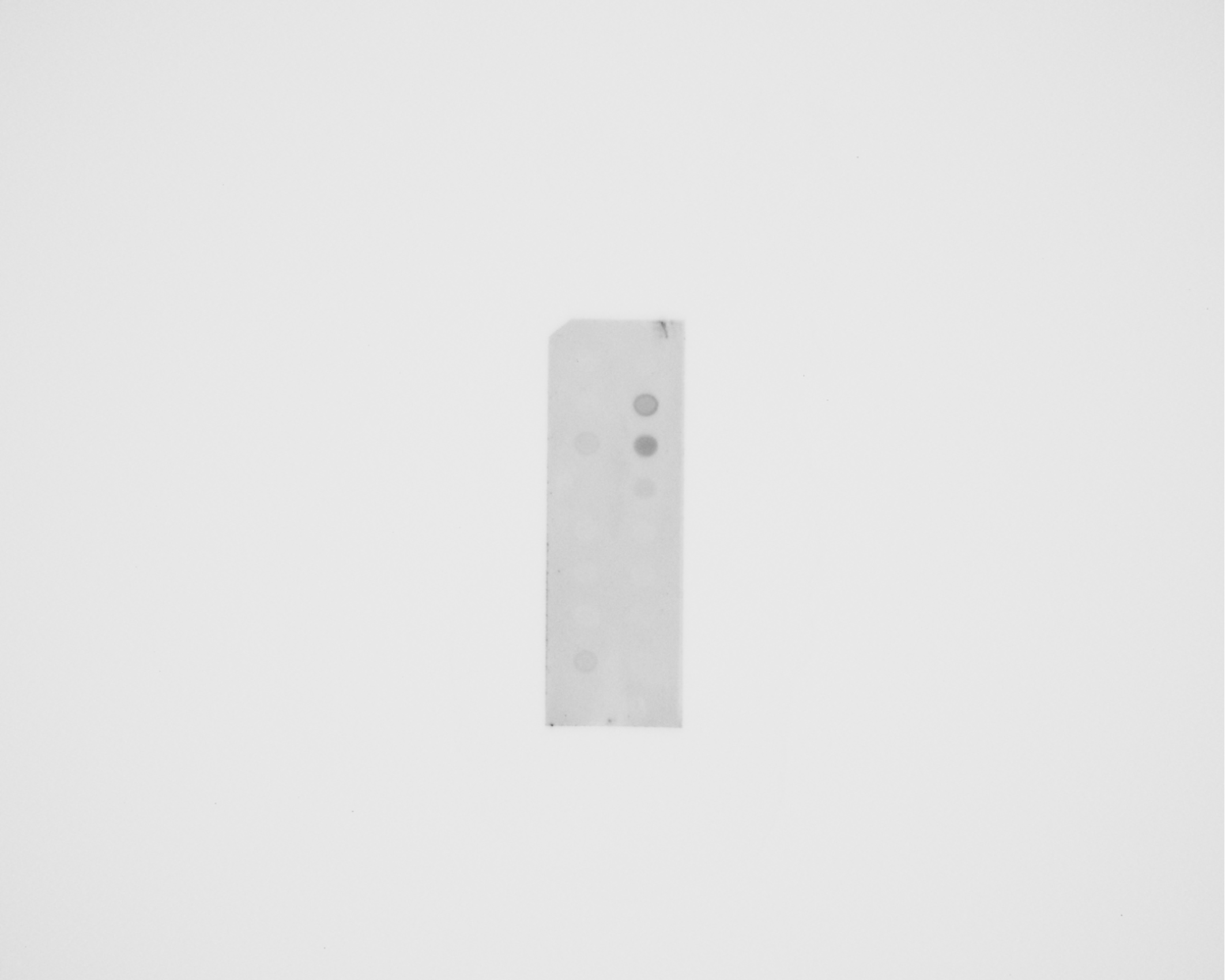

Supplement: Figure 1—source data 2. [file elife-105541-fig1-data2.zip › Figure 1-Source Data 2/Figure 1F-Source data 5.tif]

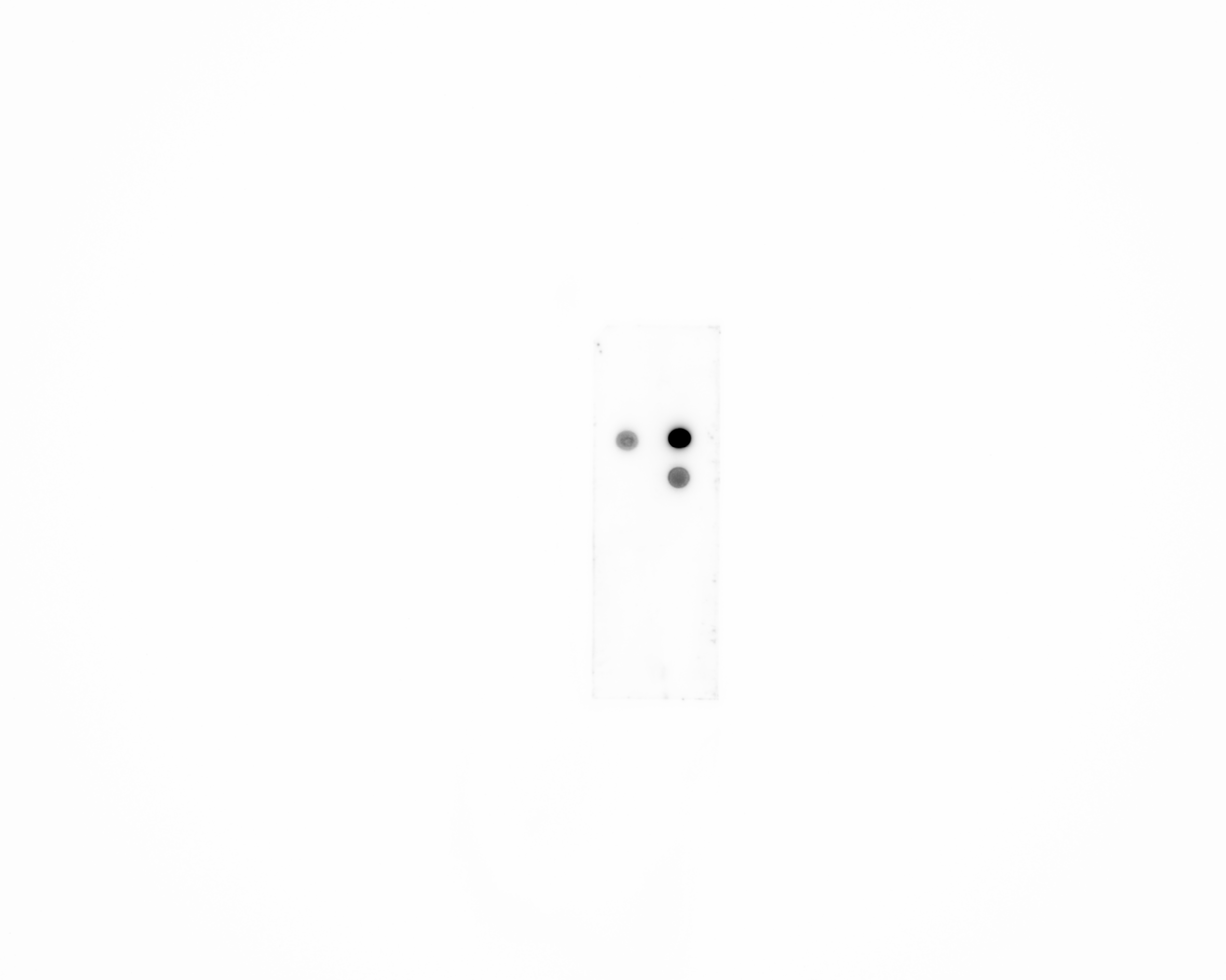

Supplement: Figure 1—source data 2. [file elife-105541-fig1-data2.zip › Figure 1-Source Data 2/Figure 1F-Source data 6.tif]

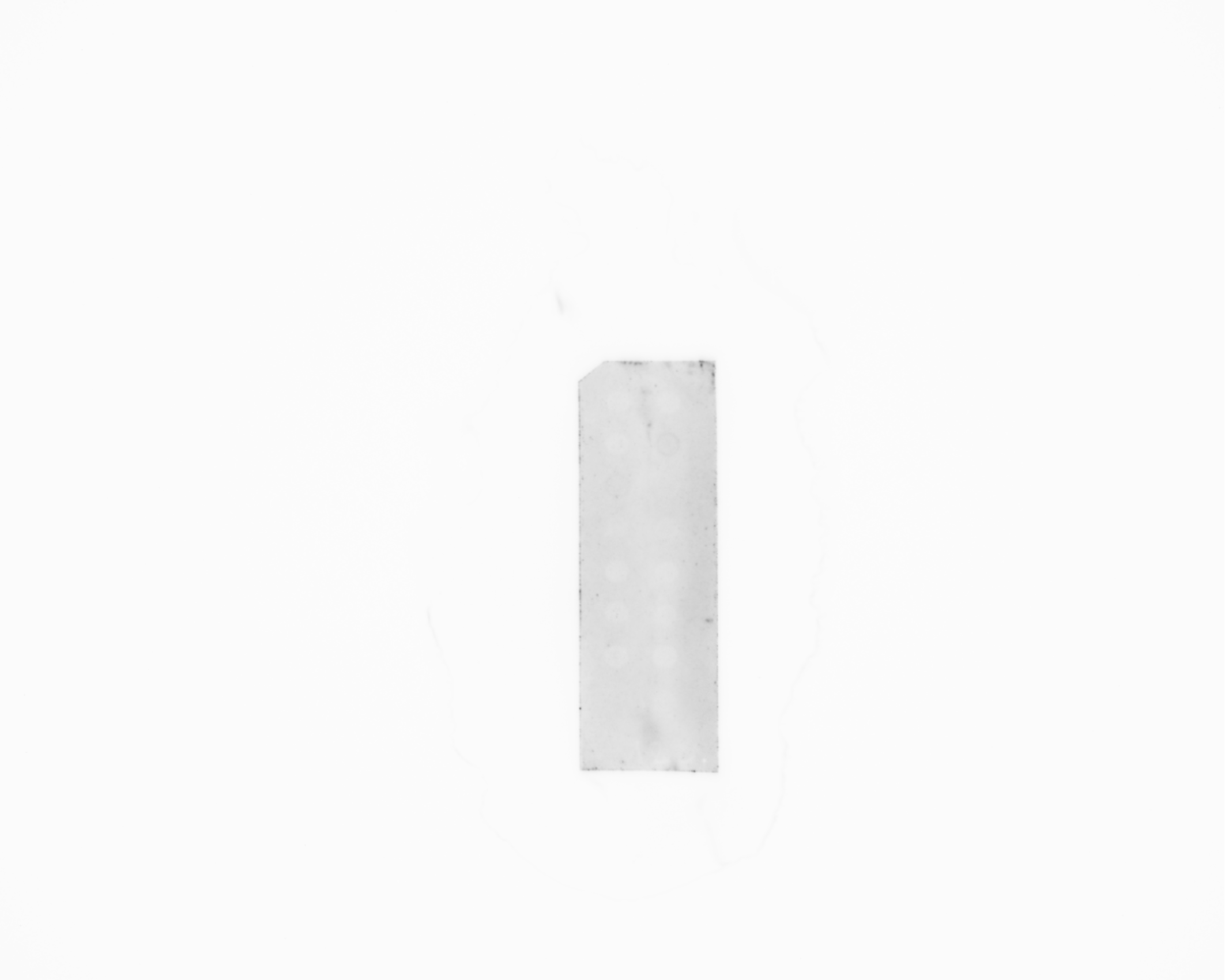

Supplement: Figure 1—source data 2. [file elife-105541-fig1-data2.zip › Figure 1-Source Data 2/Figure 1F-Source data 7.tif]

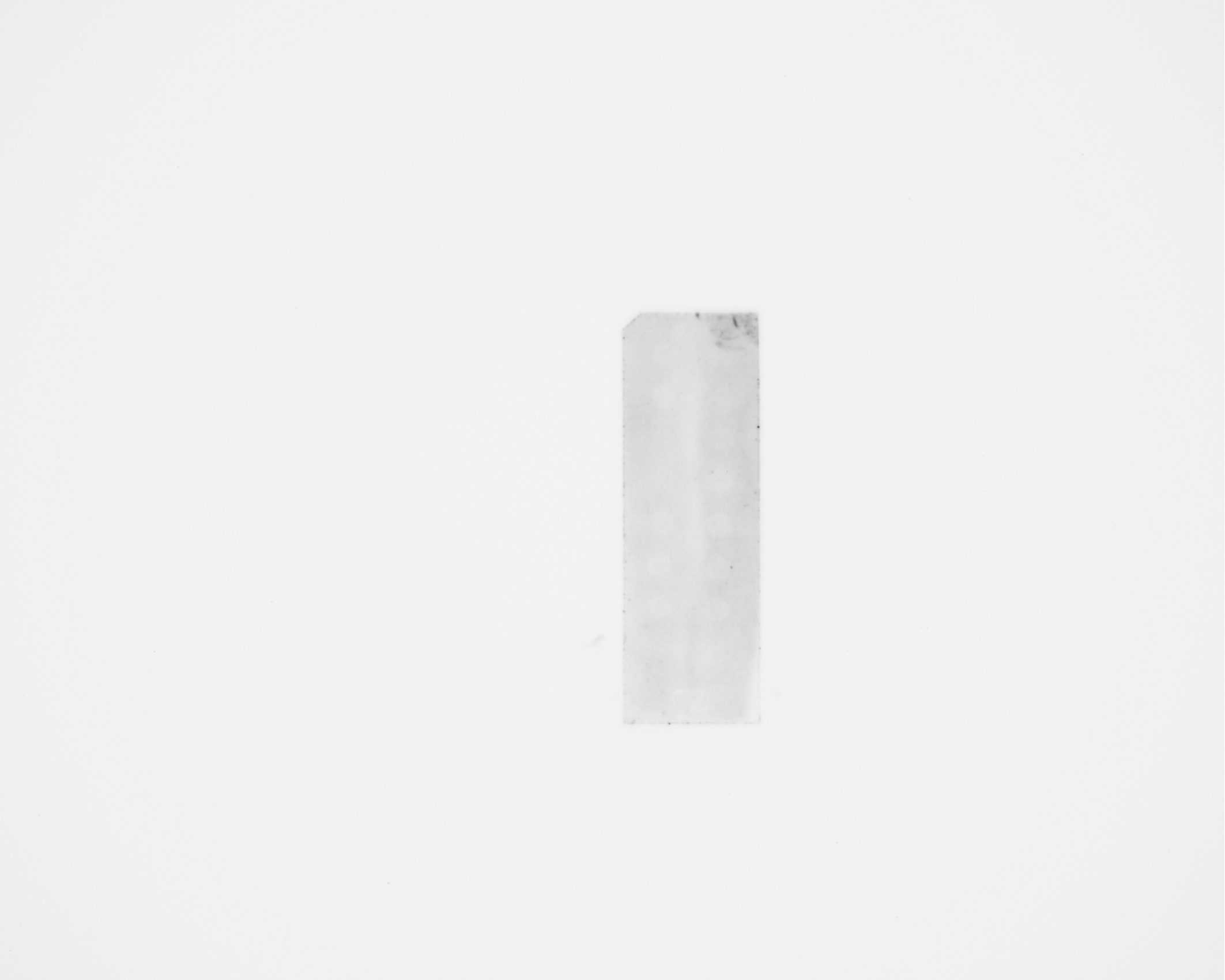

Supplement: Figure 1—source data 2. [file elife-105541-fig1-data2.zip › Figure 1-Source Data 2/Figure 1F-Source data 8.tif]

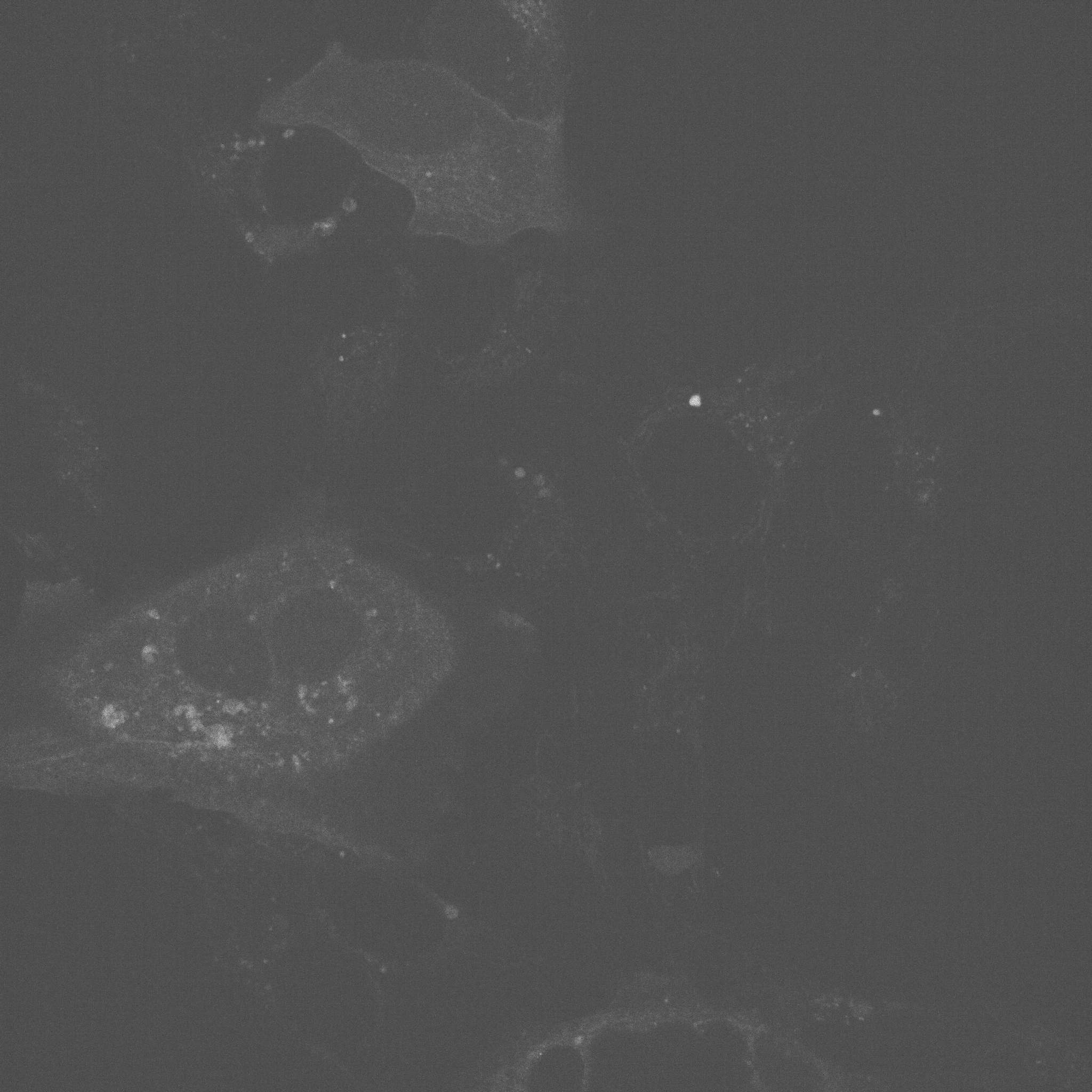

Supplement: Figure 1—figure supplement 1—source data 1. [file elife-105541-fig1-figsupp1-data1.zip › Figure 1 – figure supplement 1-Source Data 1/Figure 1 – figure supplement 1A-Source Data.tif]

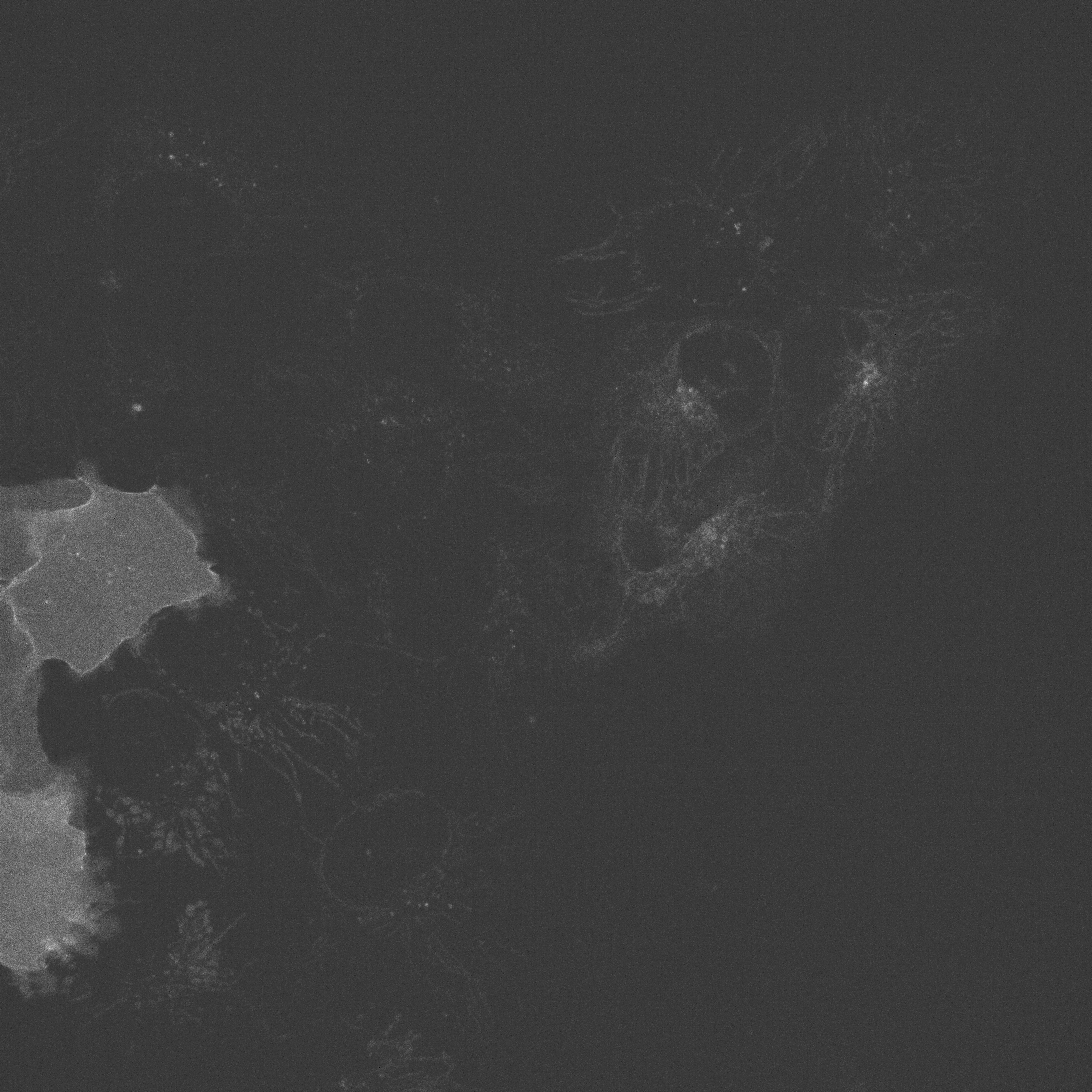

Supplement: Figure 1—figure supplement 1—source data 1. [file elife-105541-fig1-figsupp1-data1.zip › Figure 1 – figure supplement 1-Source Data 1/Figure 1 – figure supplement 1B-Source Data.tif]

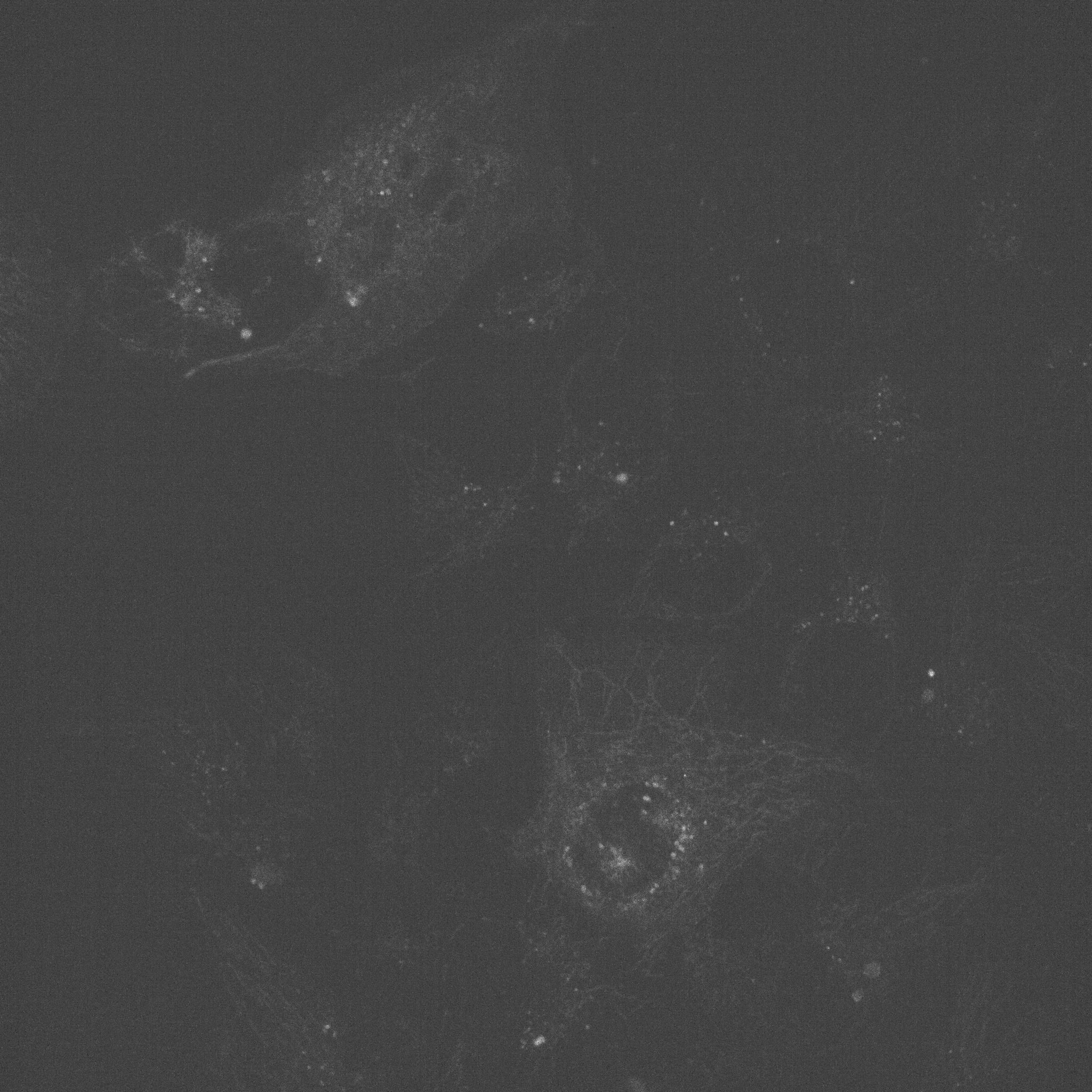

Supplement: Figure 1—figure supplement 1—source data 1. [file elife-105541-fig1-figsupp1-data1.zip › Figure 1 – figure supplement 1-Source Data 1/Figure 1 – figure supplement 1C-Source Data.tif]

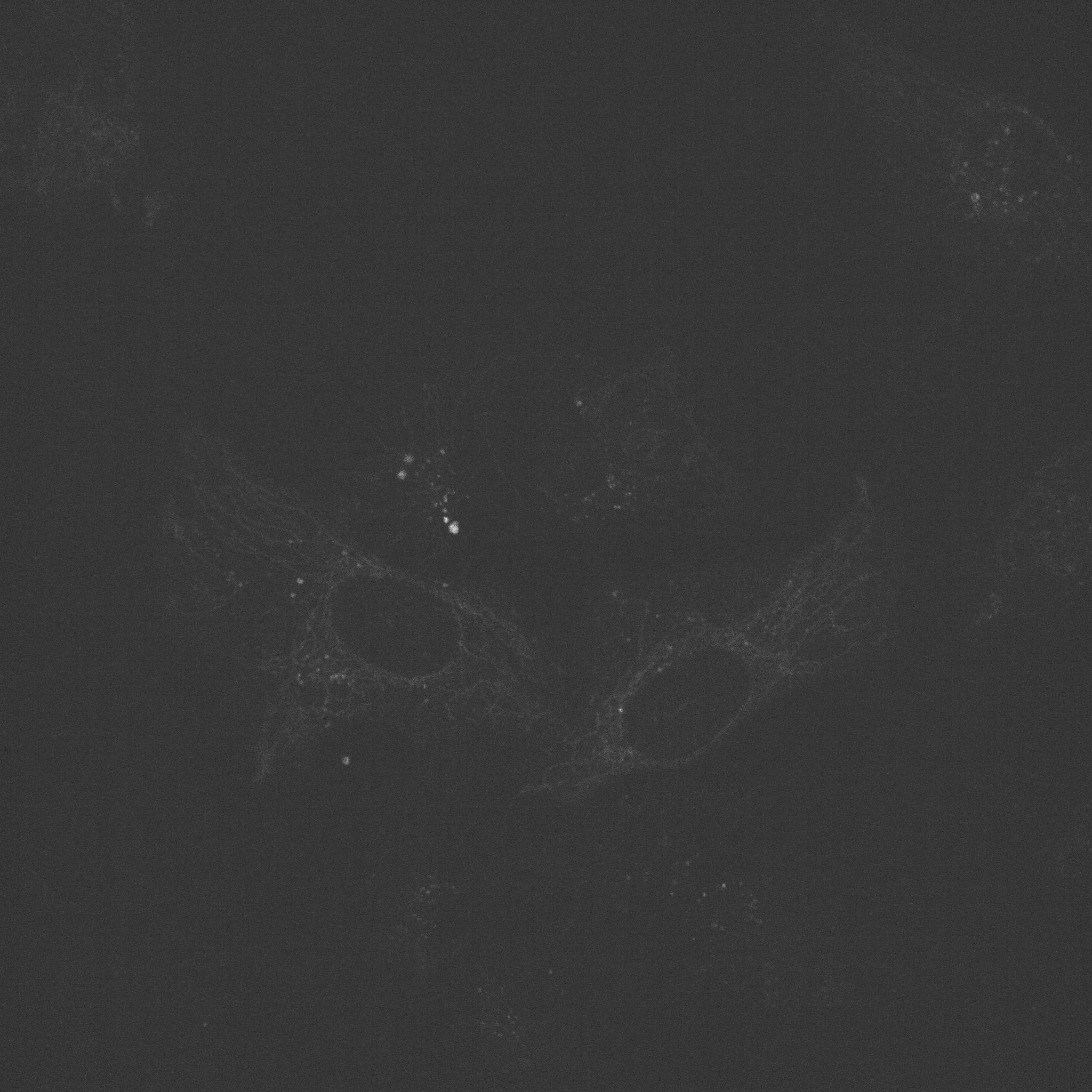

Supplement: Figure 1—figure supplement 1—source data 1. [file elife-105541-fig1-figsupp1-data1.zip › Figure 1 – figure supplement 1-Source Data 1/Figure 1 – figure supplement 1D-Source Data.tif]

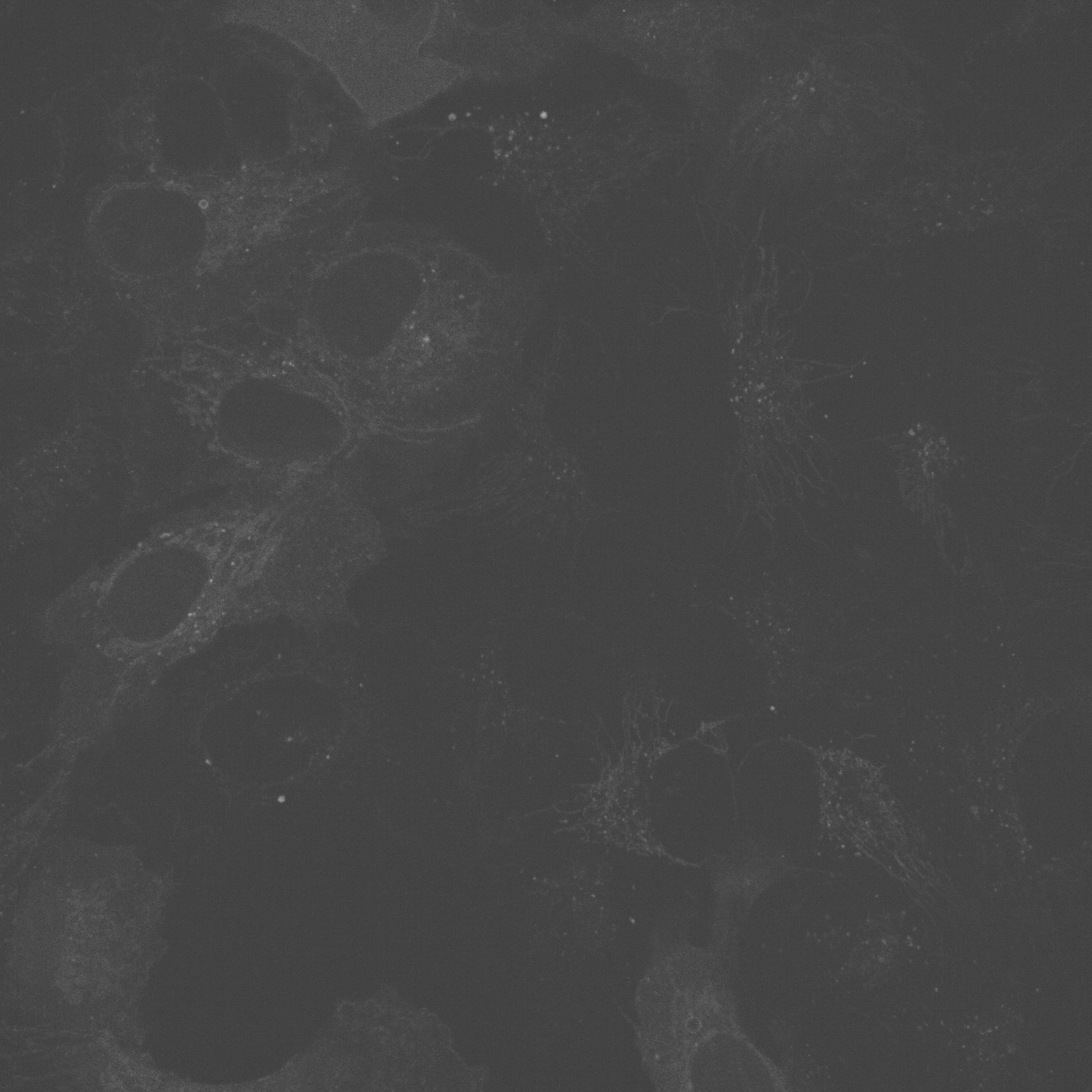

Supplement: Figure 1—figure supplement 1—source data 1. [file elife-105541-fig1-figsupp1-data1.zip › Figure 1 – figure supplement 1-Source Data 1/Figure 1 – figure supplement 1E-Source Data.tif]

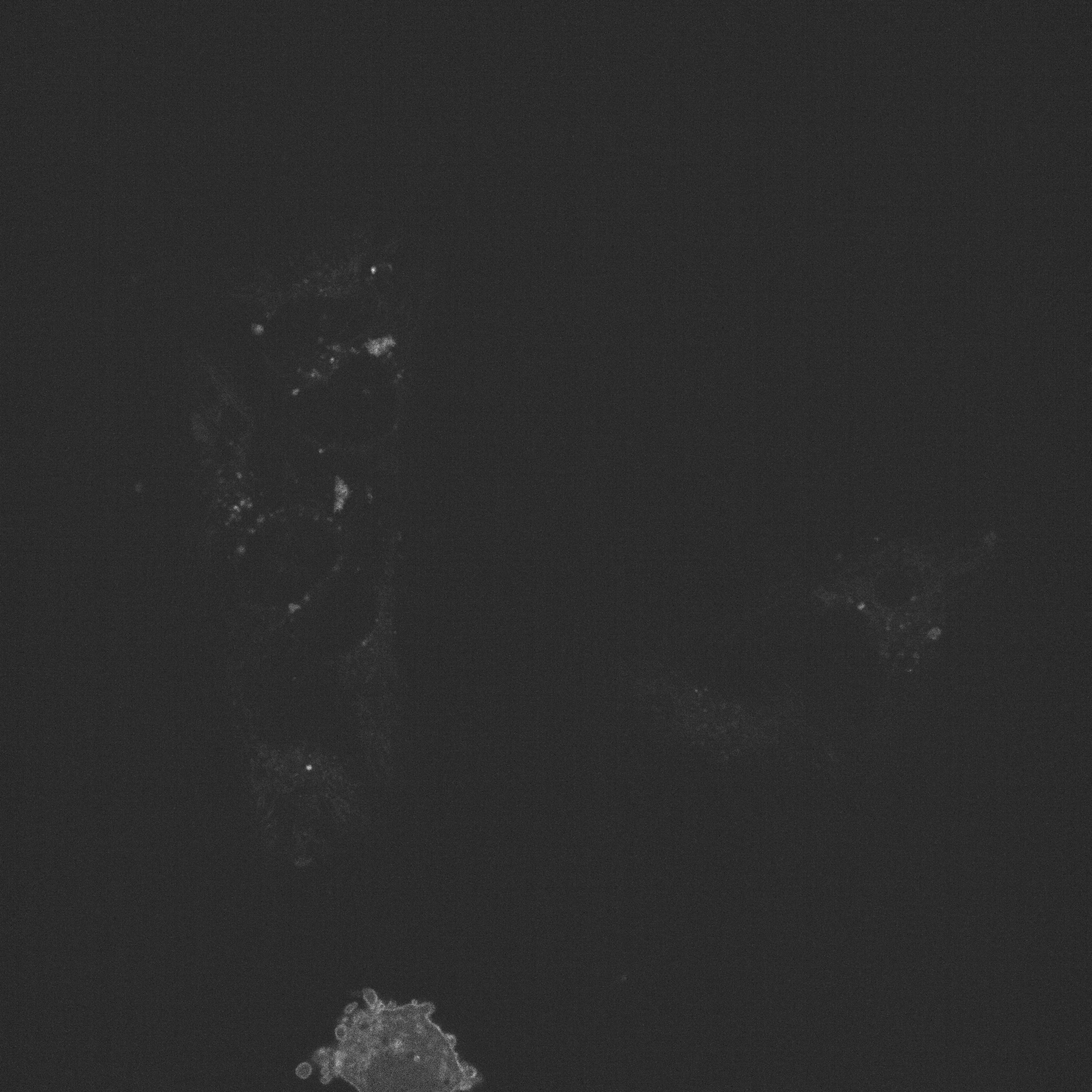

Supplement: Figure 1—figure supplement 1—source data 1. [file elife-105541-fig1-figsupp1-data1.zip › Figure 1 – figure supplement 1-Source Data 1/Figure 1 – figure supplement 1F-Source Data.tif]

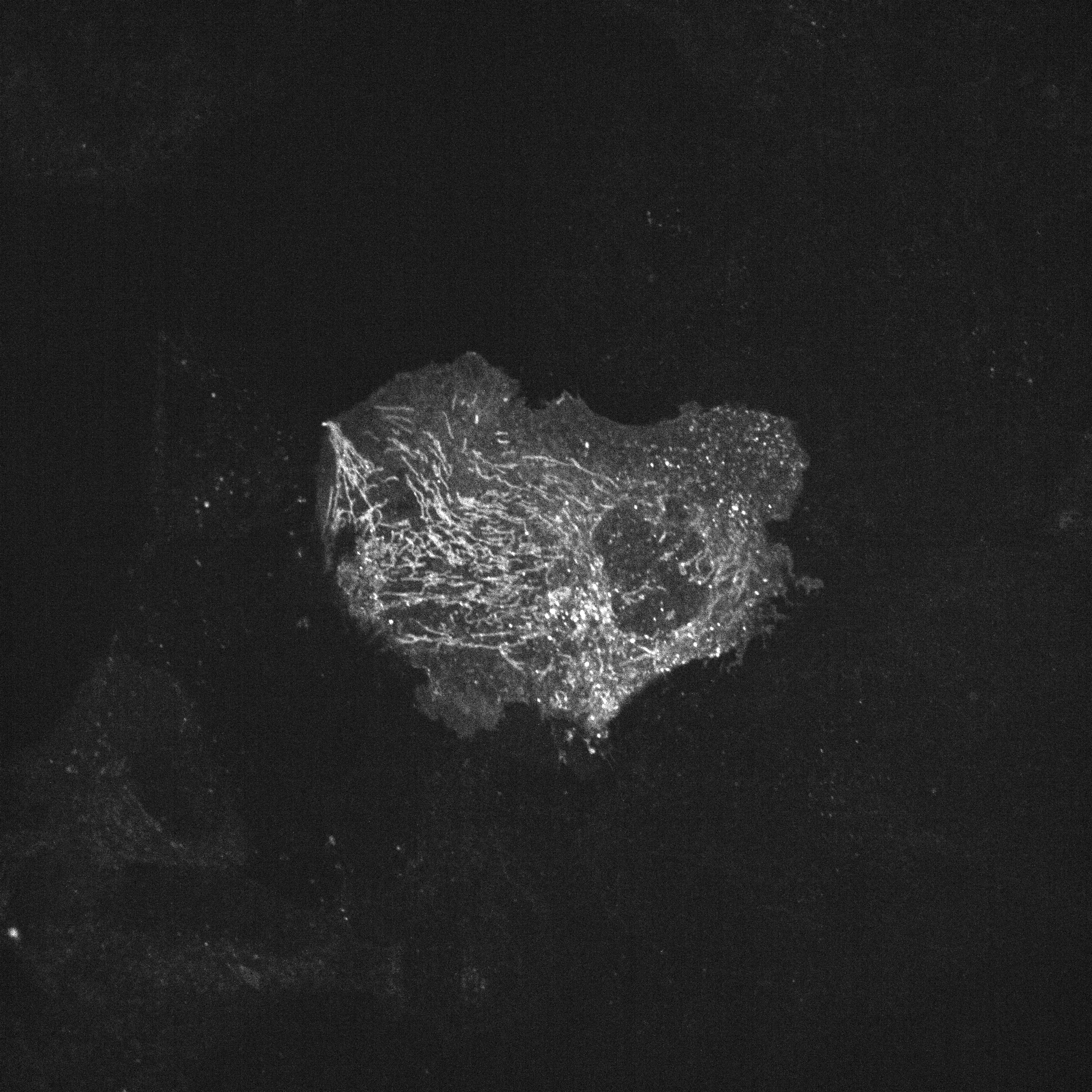

Supplement: Figure 2—source data 1. [file elife-105541-fig2-data1.zip › Figure 2-Source Data 1/Figure 2A-Source Data.tif]

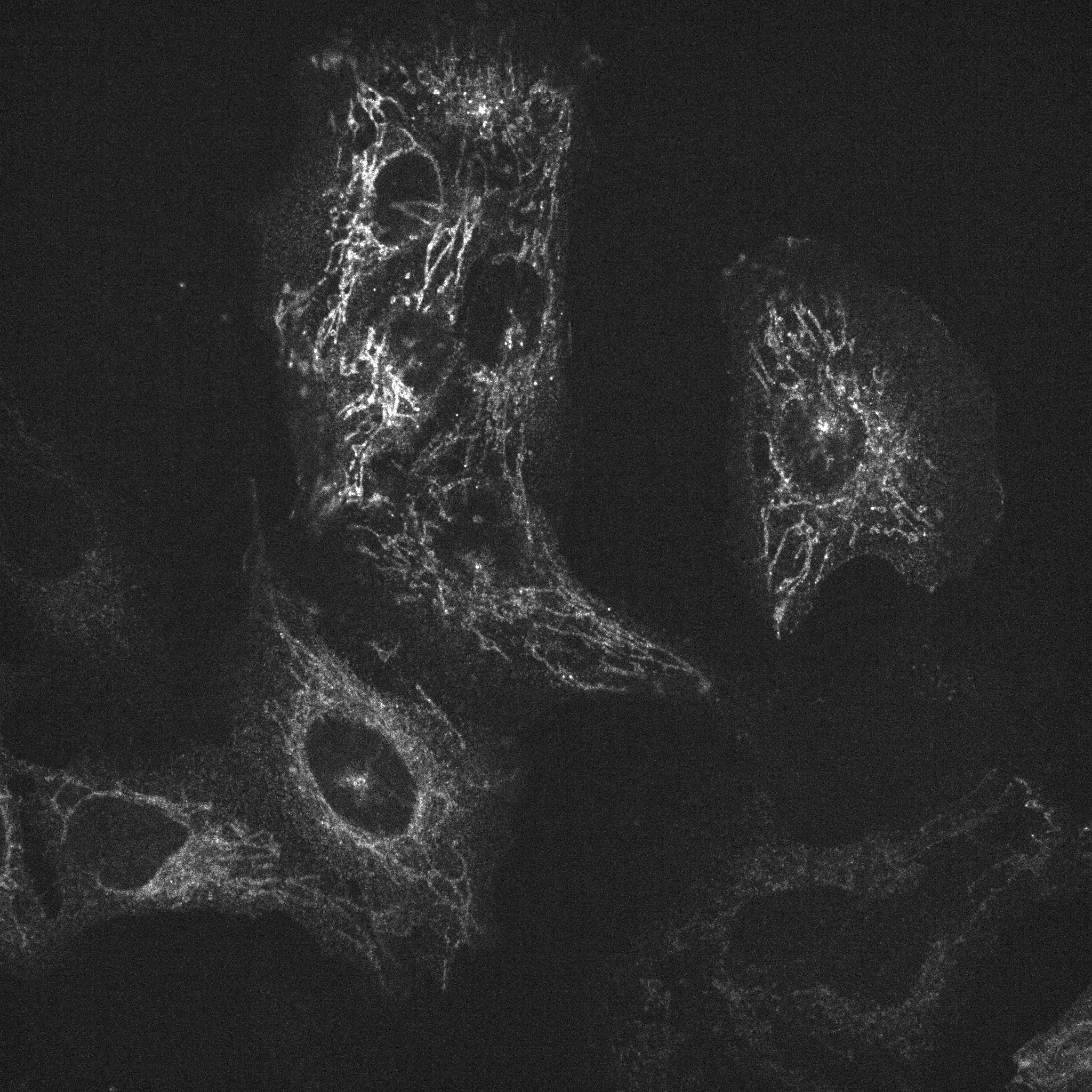

Supplement: Figure 2—source data 1. [file elife-105541-fig2-data1.zip › Figure 2-Source Data 1/Figure 2B-Source Data.tif]

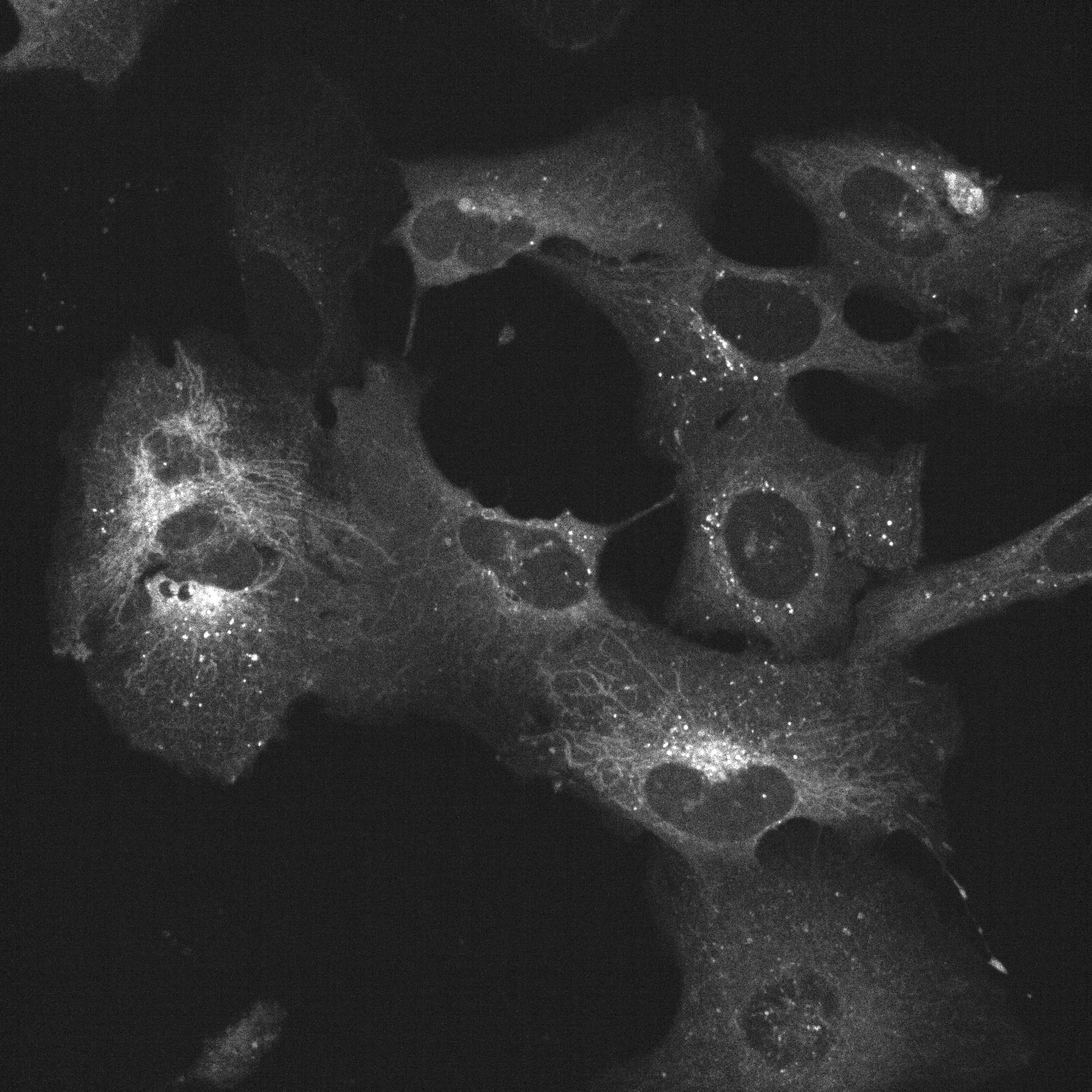

Supplement: Figure 2—source data 1. [file elife-105541-fig2-data1.zip › Figure 2-Source Data 1/Figure 2C-Source Data.tif]

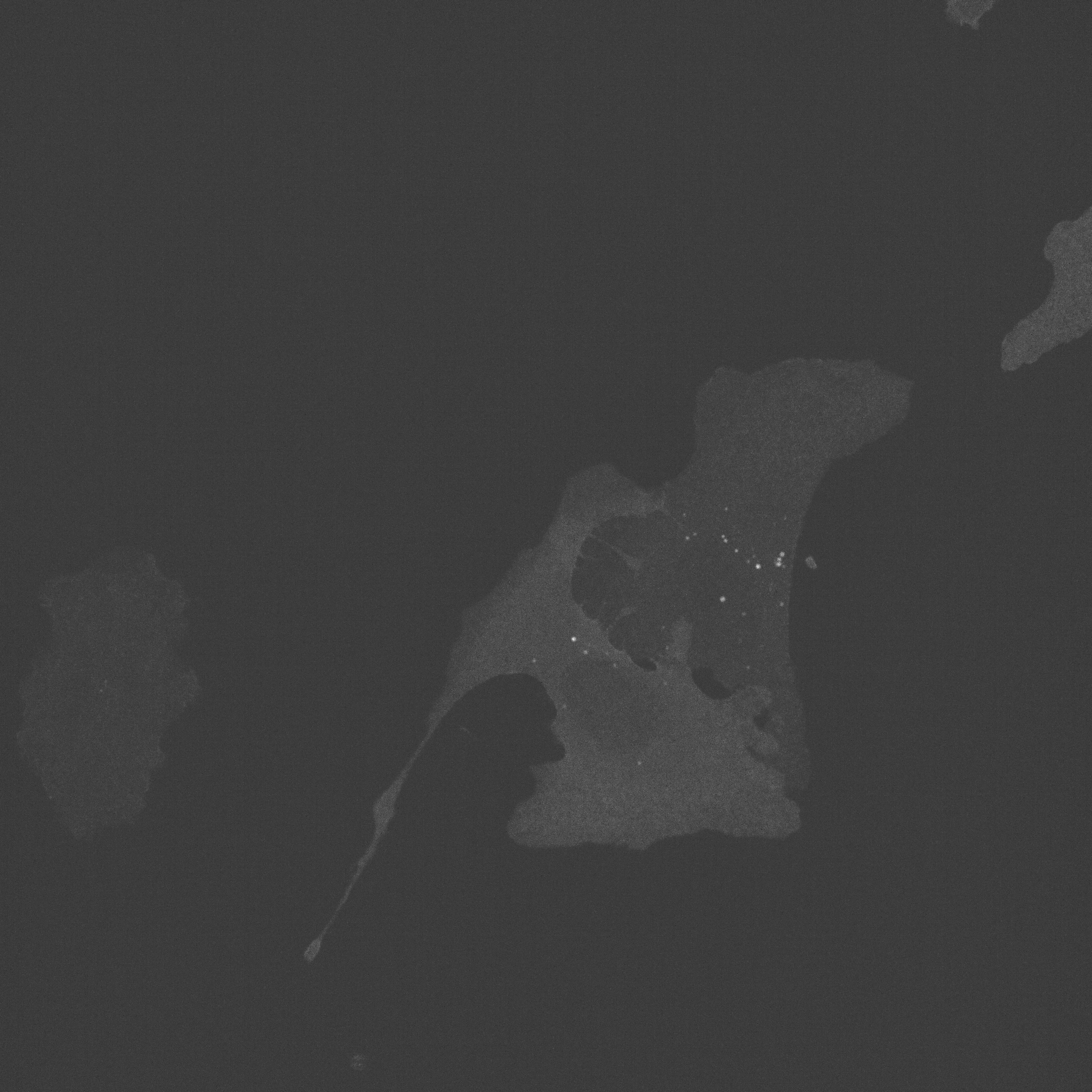

Supplement: Figure 2—source data 1. [file elife-105541-fig2-data1.zip › Figure 2-Source Data 1/Figure 2D-Source Data.tif]

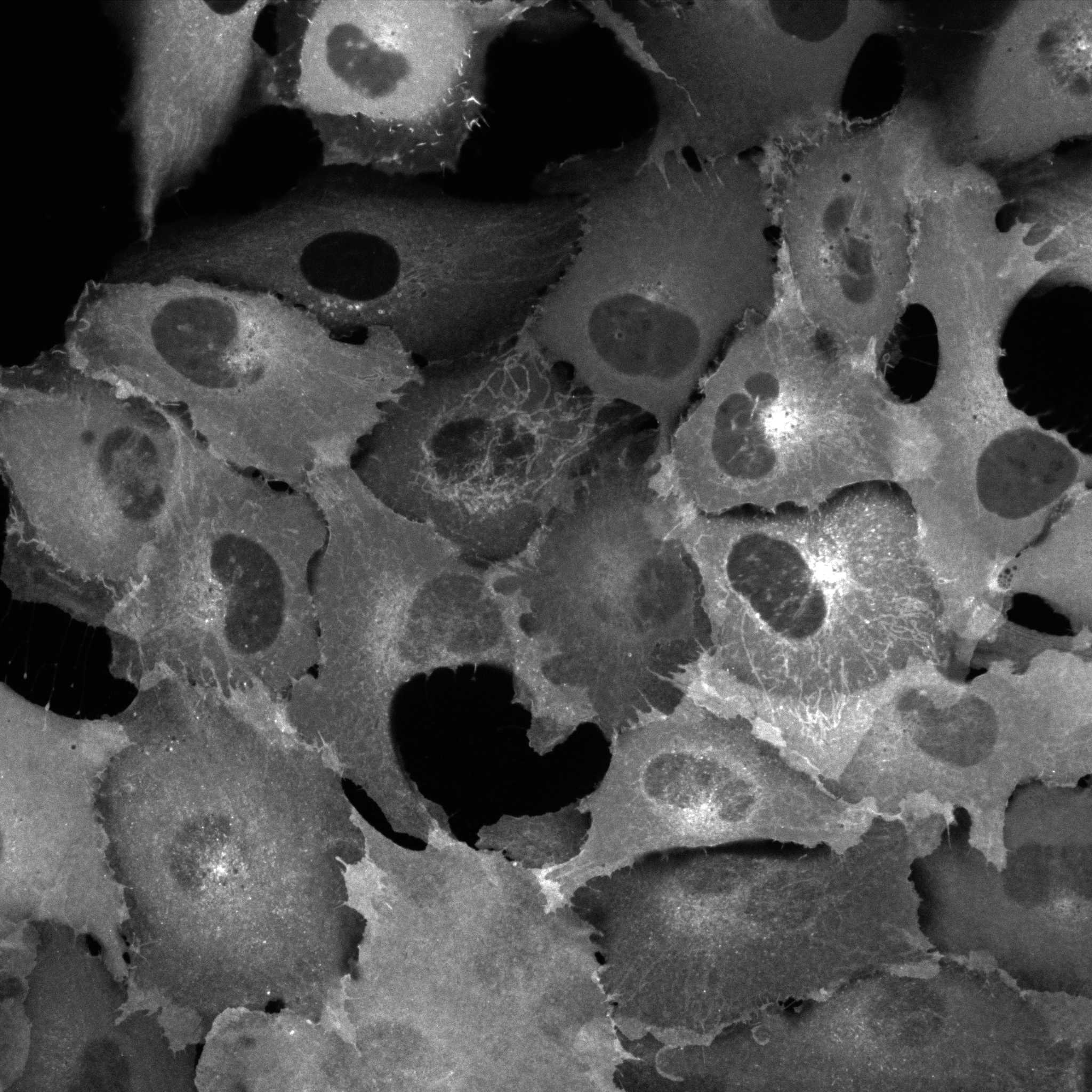

Supplement: Figure 2—source data 1. [file elife-105541-fig2-data1.zip › Figure 2-Source Data 1/Figure 2E-Source Data 1.tif]

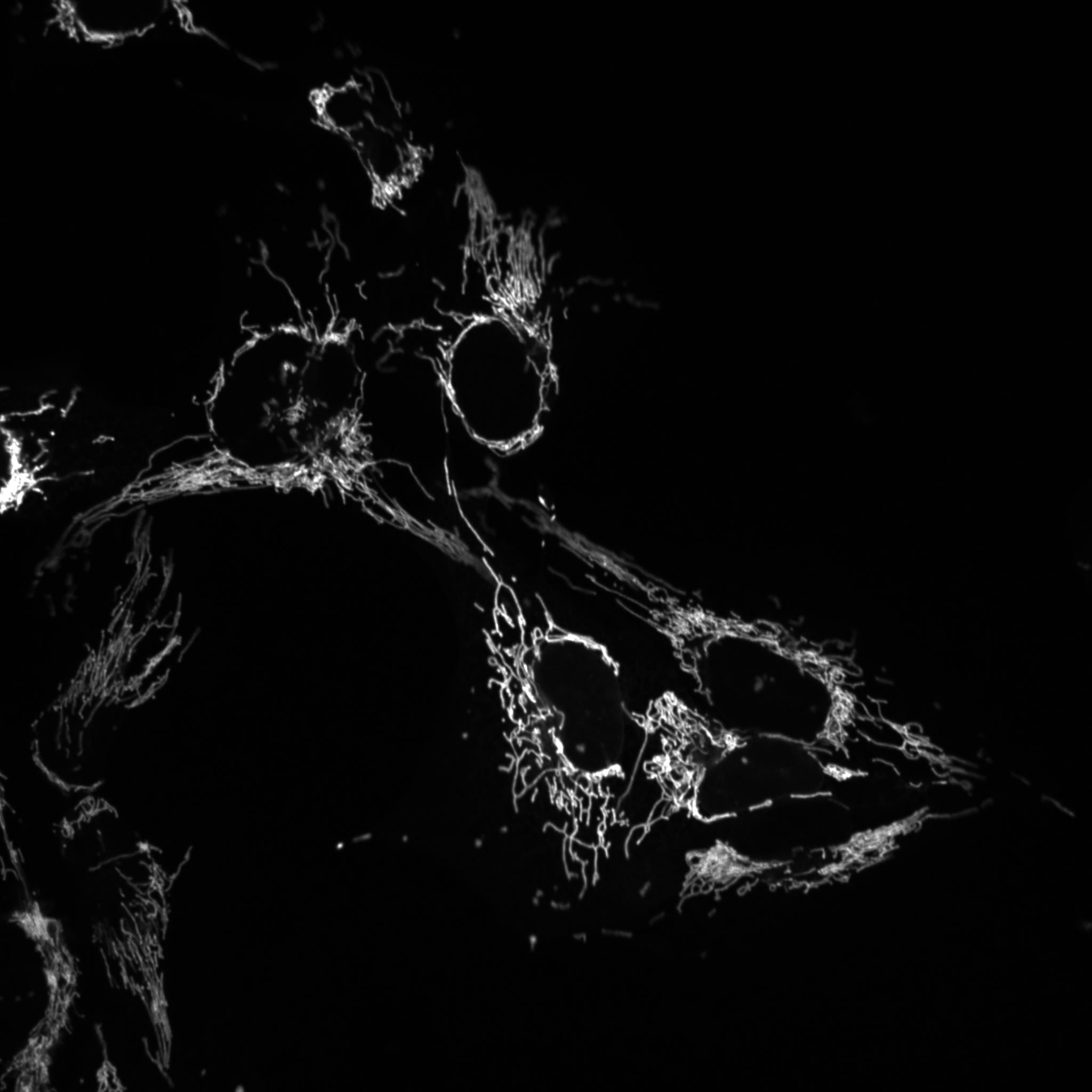

Supplement: Figure 2—source data 1. [file elife-105541-fig2-data1.zip › Figure 2-Source Data 1/Figure 2E-Source Data 2.tif]

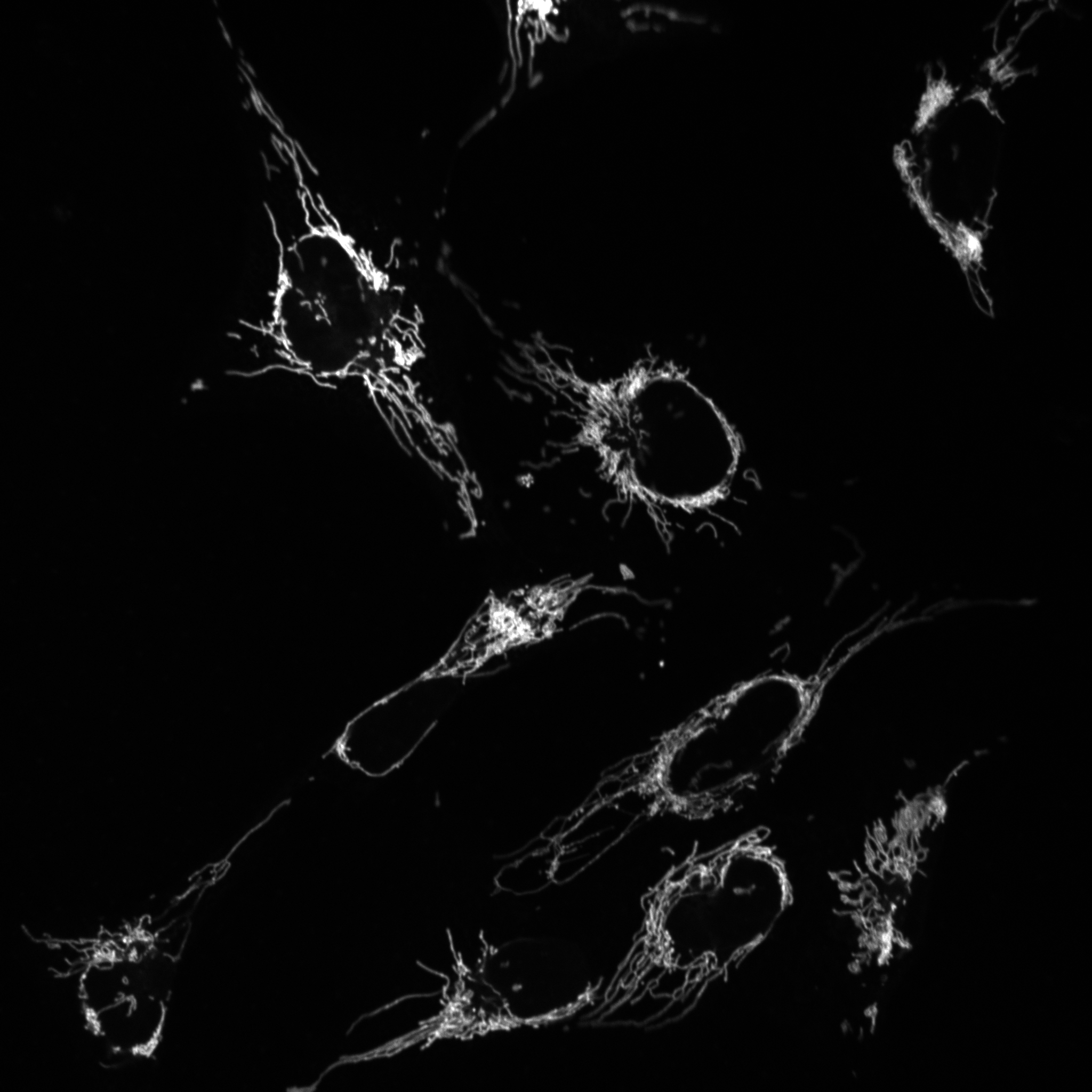

Supplement: Figure 2—source data 1. [file elife-105541-fig2-data1.zip › Figure 2-Source Data 1/Figure 2E-Source Data 3.tif]

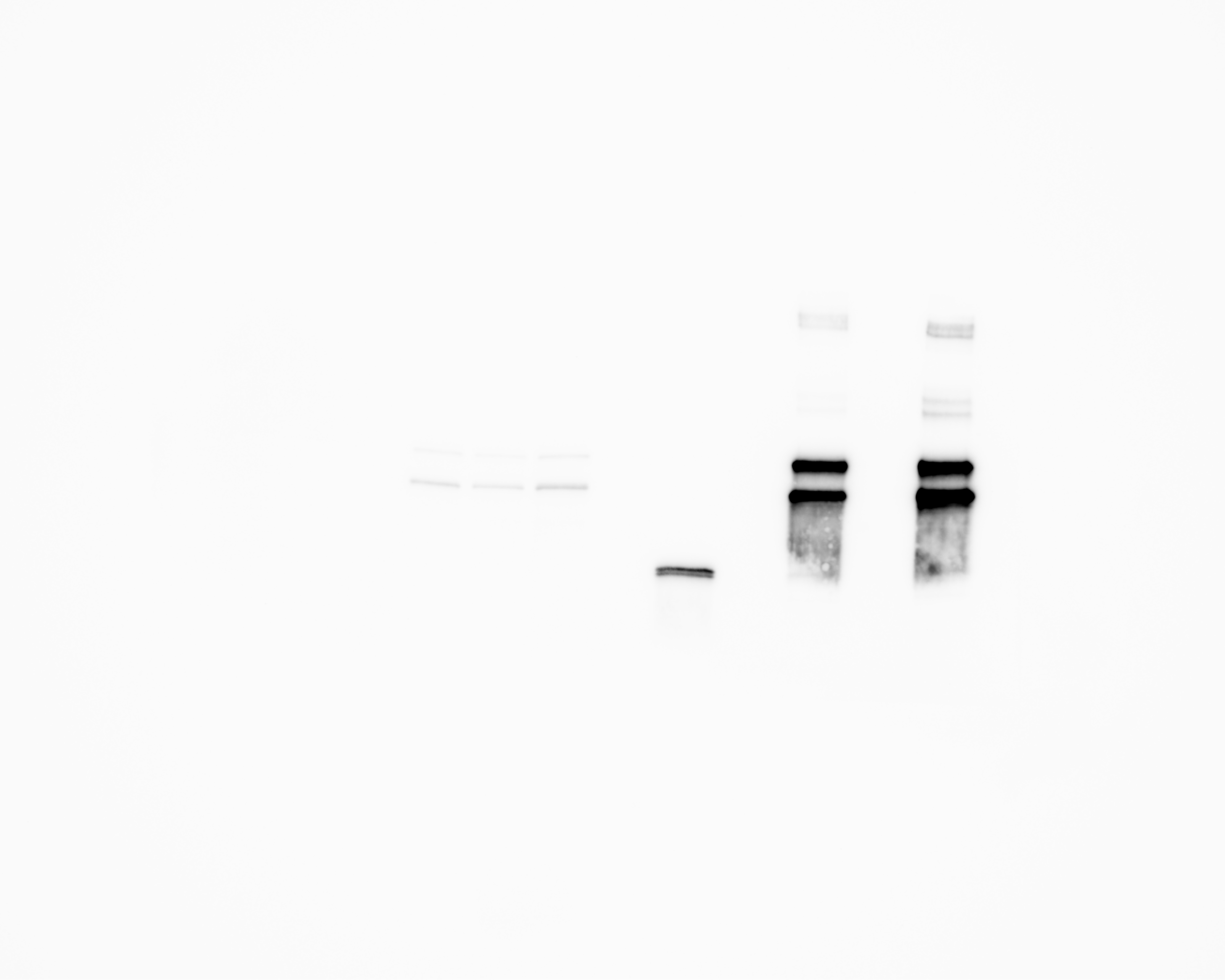

Supplement: Figure 2—source data 2. [file elife-105541-fig2-data2.zip › Figure 2-Source Data 2/Figure 2F-Source Data 1.tif]

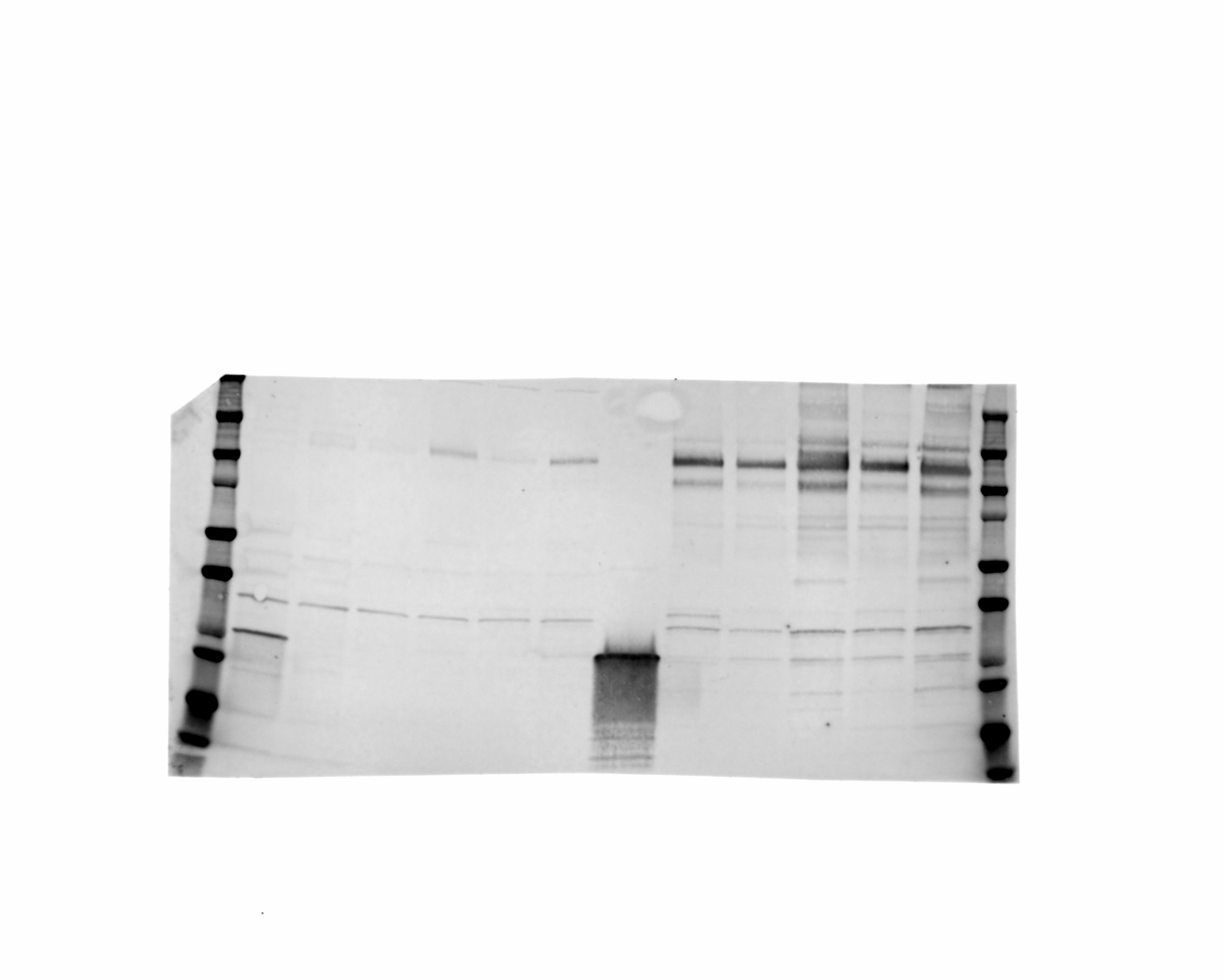

Supplement: Figure 2—source data 2. [file elife-105541-fig2-data2.zip › Figure 2-Source Data 2/Figure 2F-Source Data 2.tif]

Figure 2F

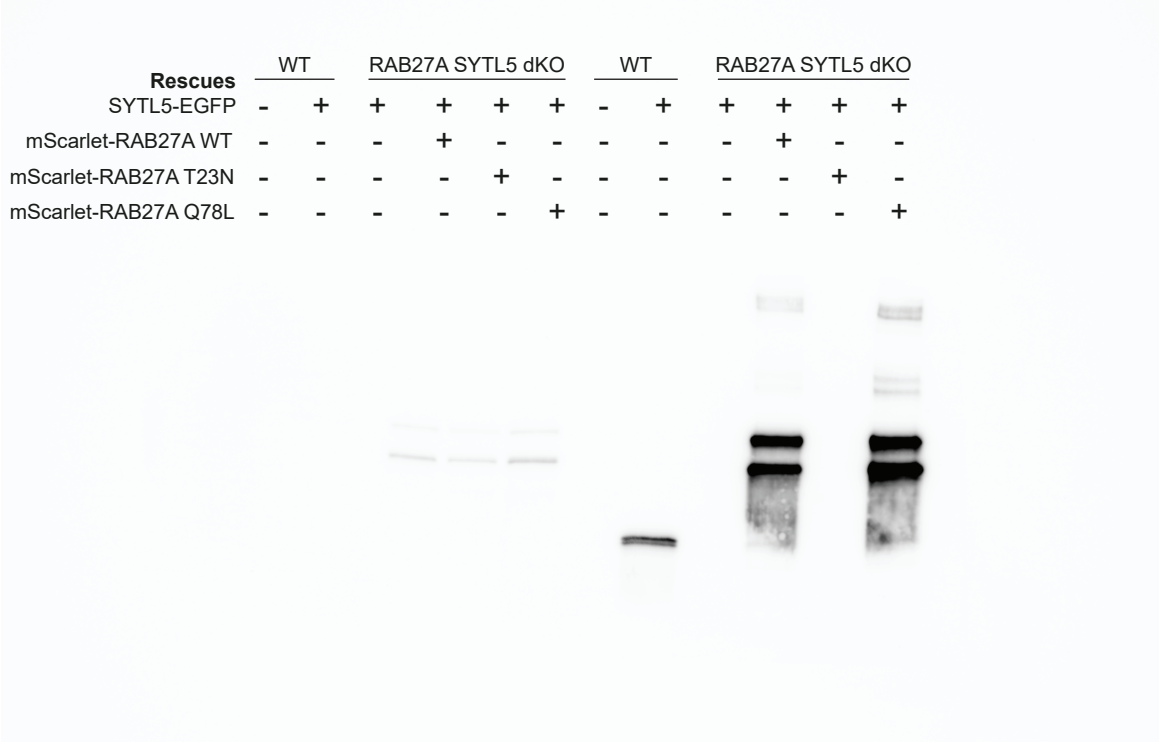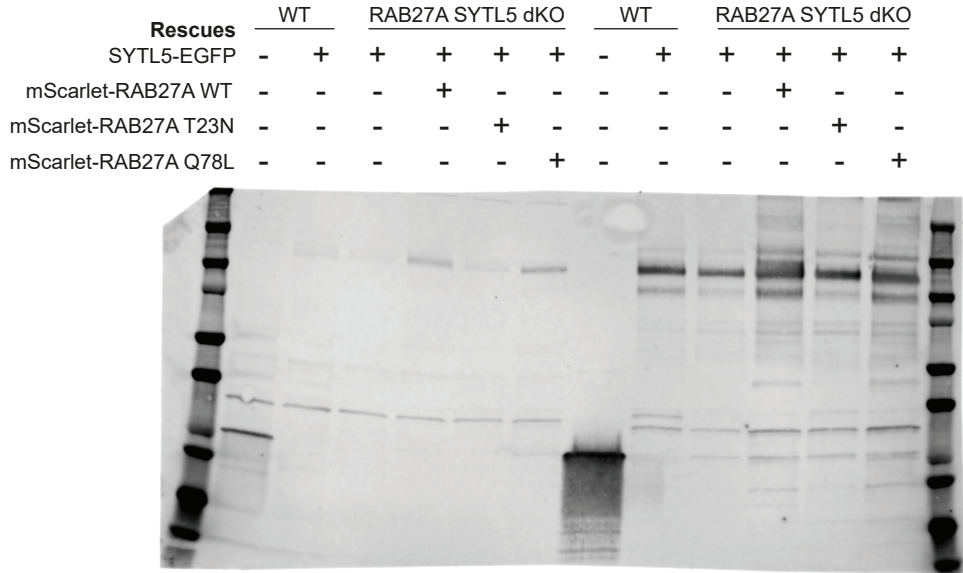

Supplement: Figure 2—source data 3. [file elife-105541-fig2-data3.zip › Figure 2-Source Data 3/Figure 2F-Source Data 3.pdf]

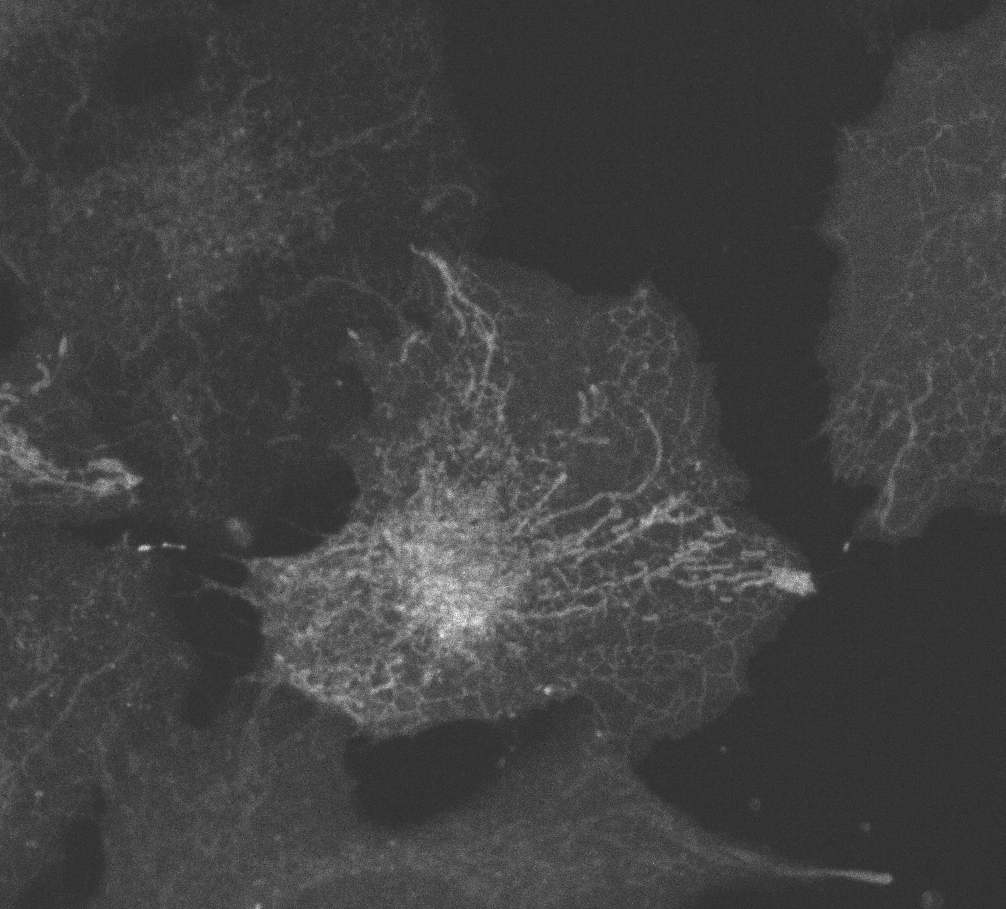

Supplement: Figure 2—figure supplement 1—source data 1. [file elife-105541-fig2-figsupp1-data1.zip › Figure 2 – figure supplement 1-Source Data 1/Figure 2 – figure supplement 1A-Source Data.tif]

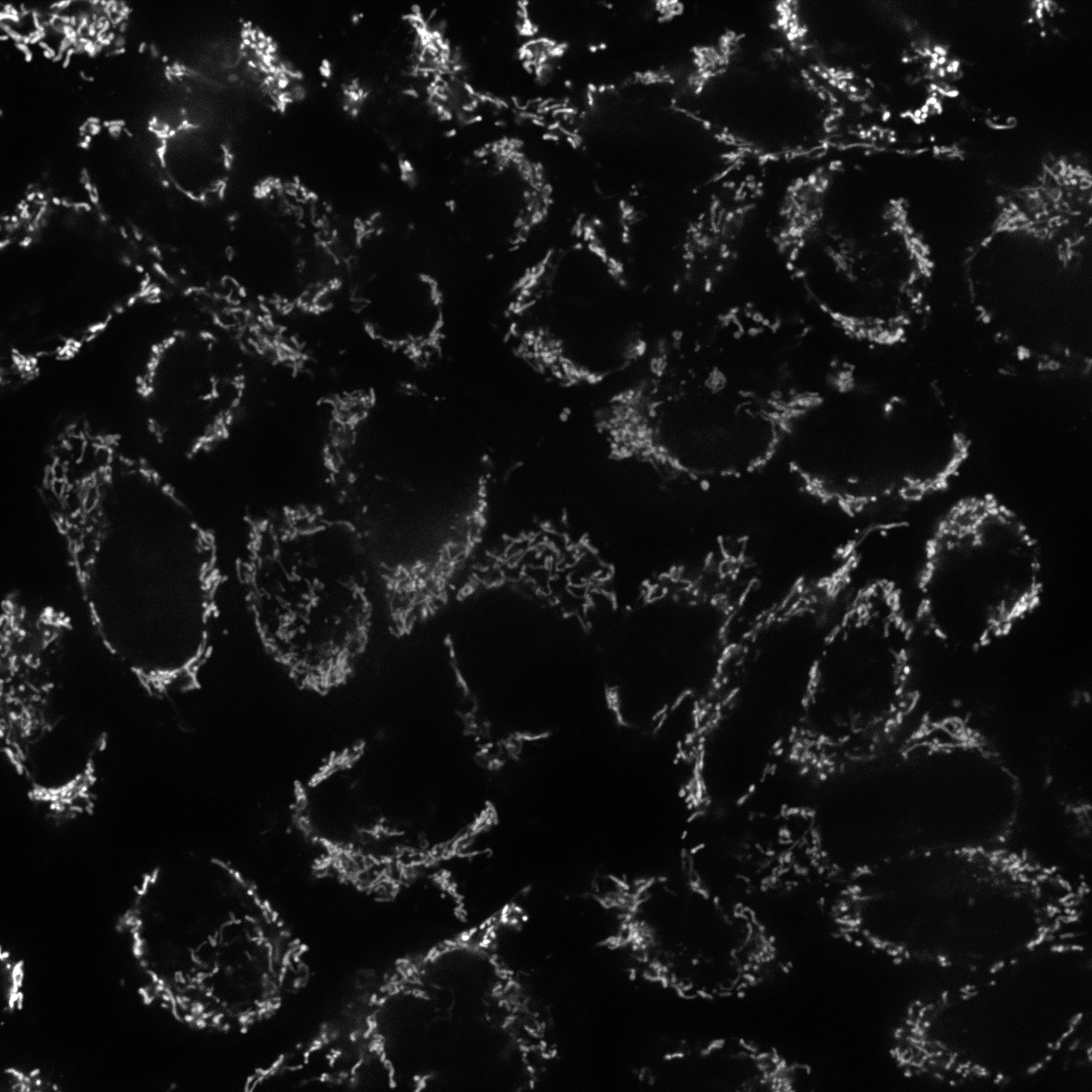

Supplement: Figure 2—figure supplement 1—source data 1. [file elife-105541-fig2-figsupp1-data1.zip › Figure 2 – figure supplement 1-Source Data 1/Figure 2 – figure supplement 1C-Source Data.tif]

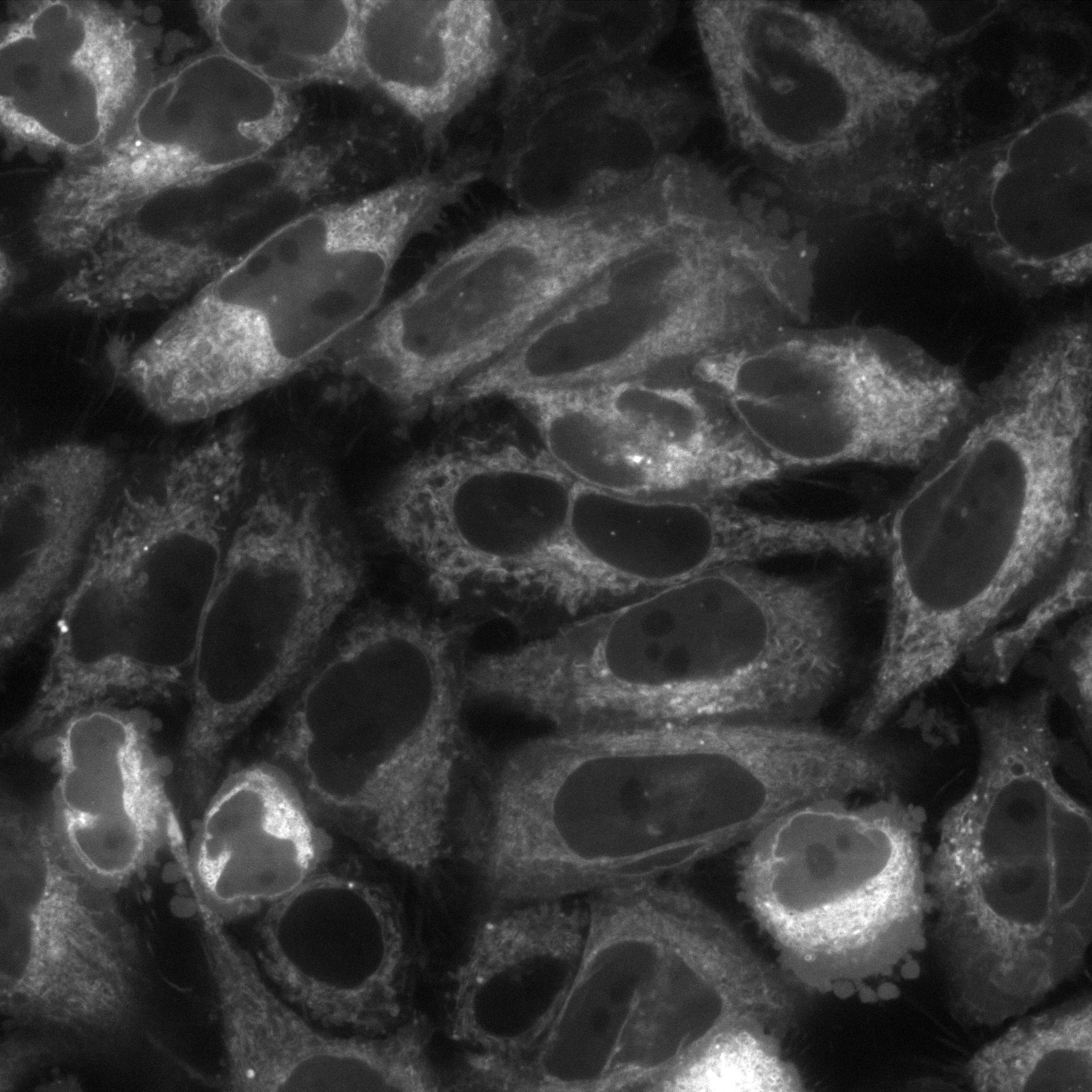

Supplement: Figure 2—figure supplement 1—source data 1. [file elife-105541-fig2-figsupp1-data1.zip › Figure 2 – figure supplement 1-Source Data 1/Figure 2 – figure supplement 1D-Source Data.tif]

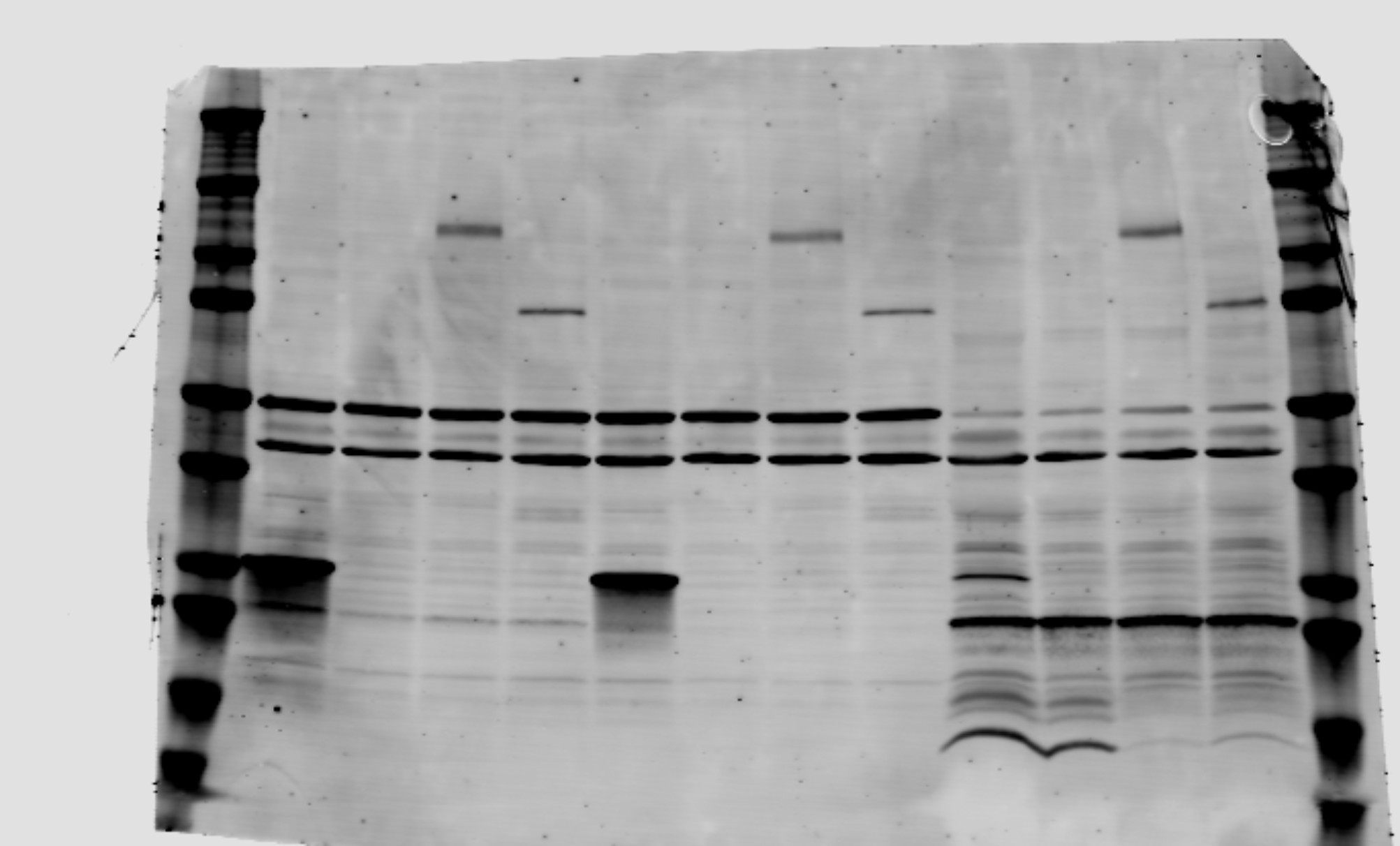

Supplement: Figure 2—figure supplement 1—source data 2. [file elife-105541-fig2-figsupp1-data2.zip › Figure 2 – figure supplement 1-Source Data 2/Figure 2 – figure supplement 1B-Source Data 1.tif]

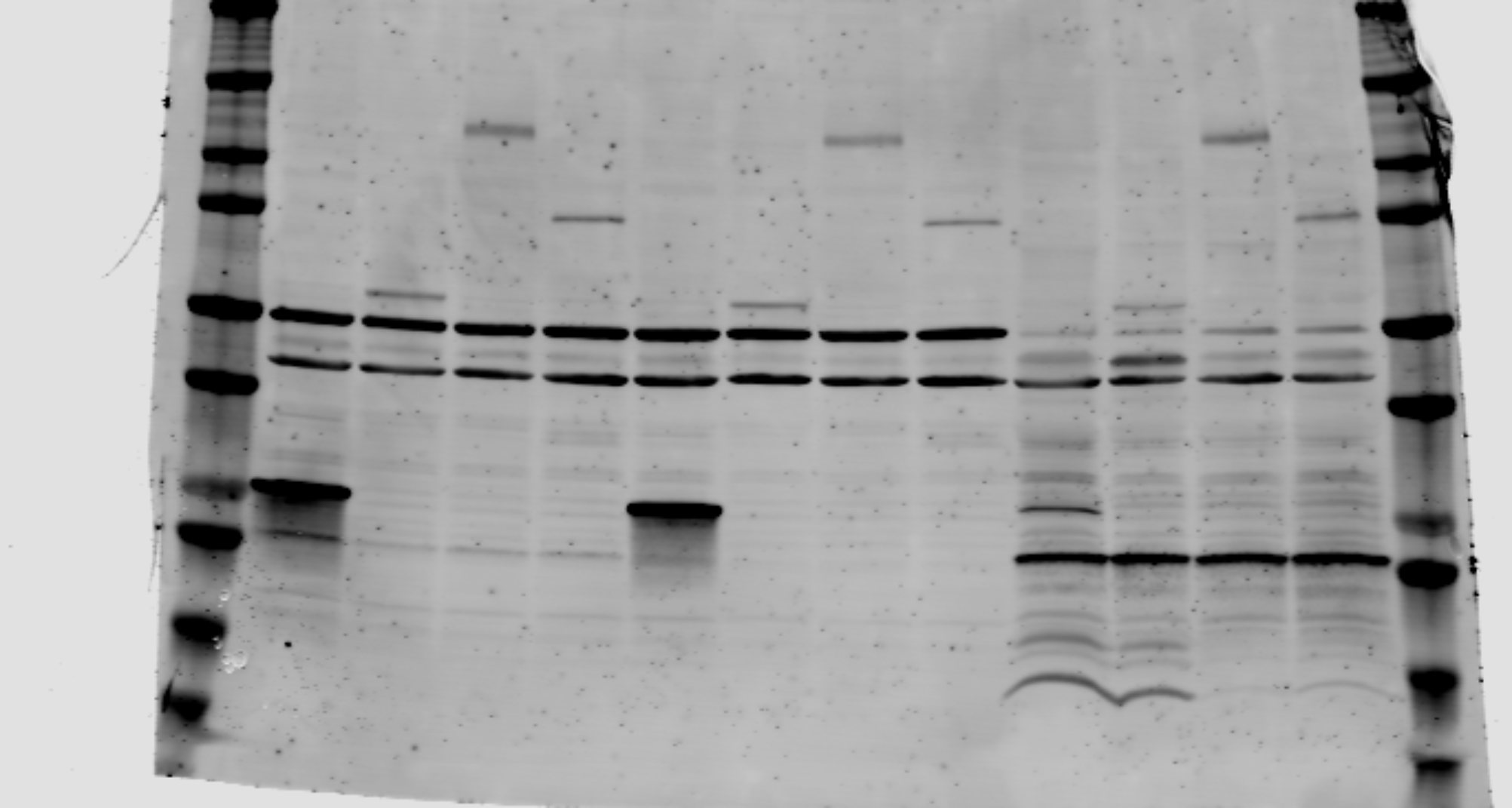

Supplement: Figure 2—figure supplement 1—source data 2. [file elife-105541-fig2-figsupp1-data2.zip › Figure 2 – figure supplement 1-Source Data 2/Figure 2 – figure supplement 1B-Source Data 2.tif]

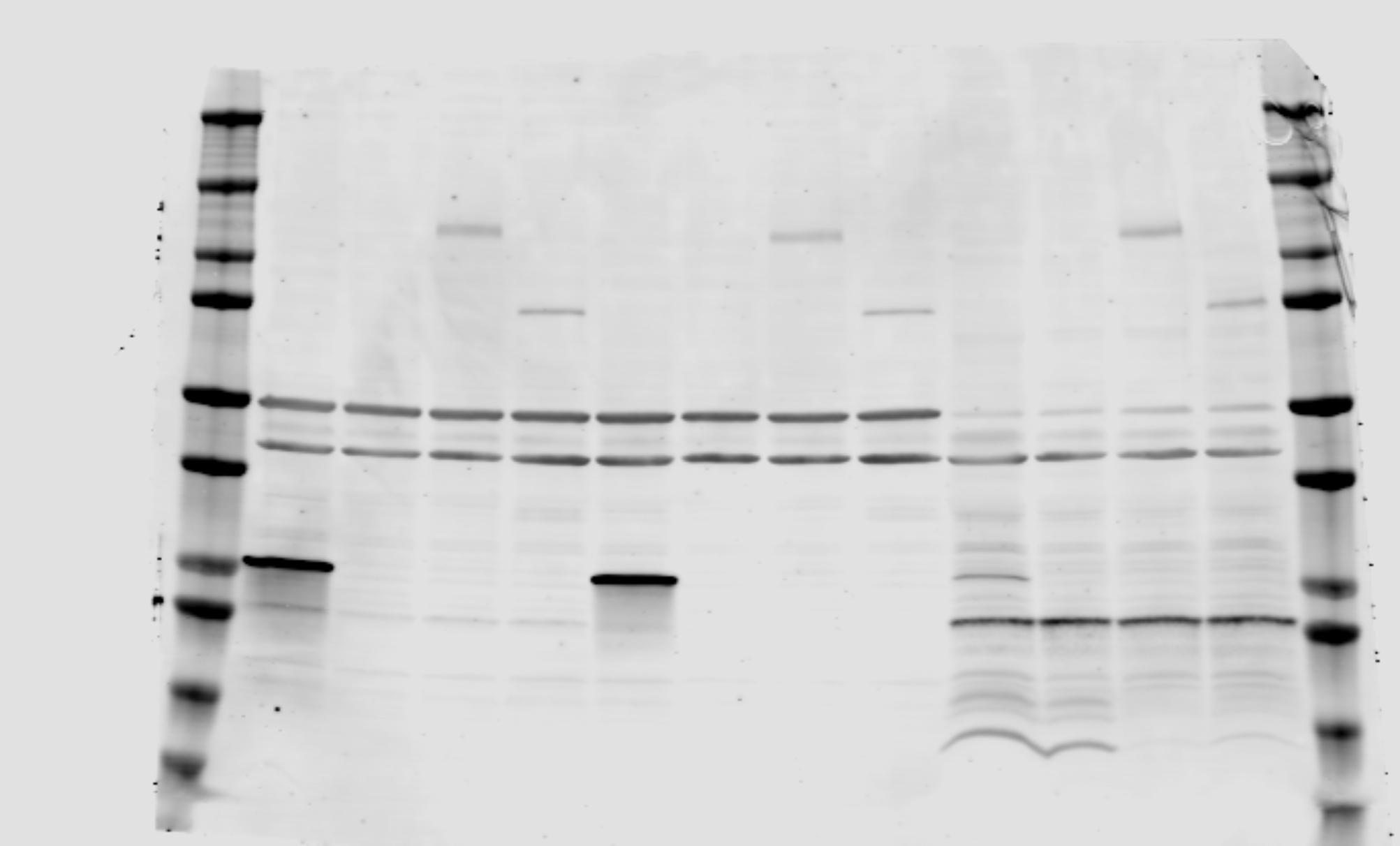

Supplement: Figure 2—figure supplement 1—source data 2. [file elife-105541-fig2-figsupp1-data2.zip › Figure 2 – figure supplement 1-Source Data 2/Figure 2 – figure supplement 1B-Source Data 3.tif]

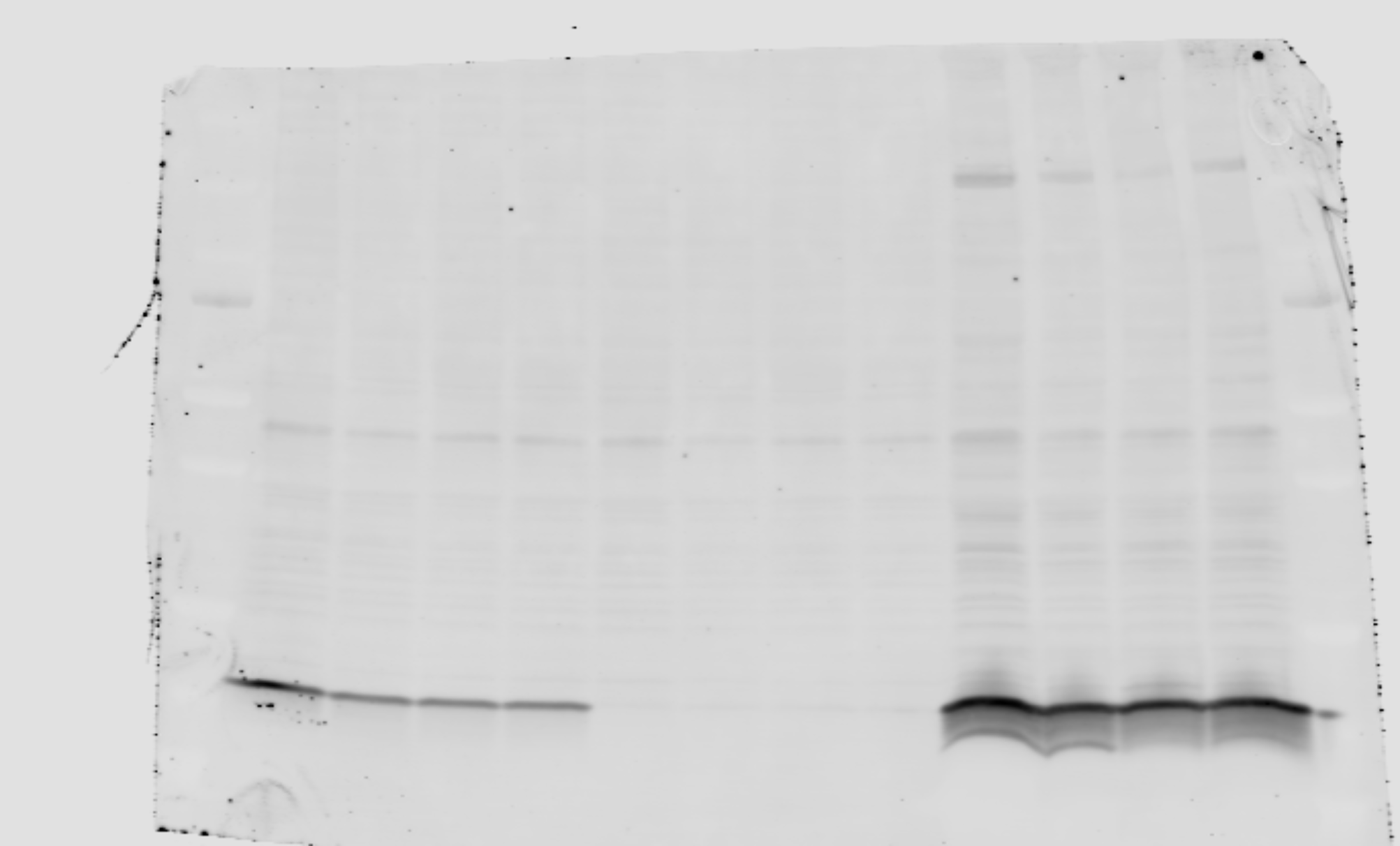

Supplement: Figure 2—figure supplement 1—source data 2. [file elife-105541-fig2-figsupp1-data2.zip › Figure 2 – figure supplement 1-Source Data 2/Figure 2 – figure supplement 1B-Source Data 4.tif]

Figure 2 – figure supplement 1B

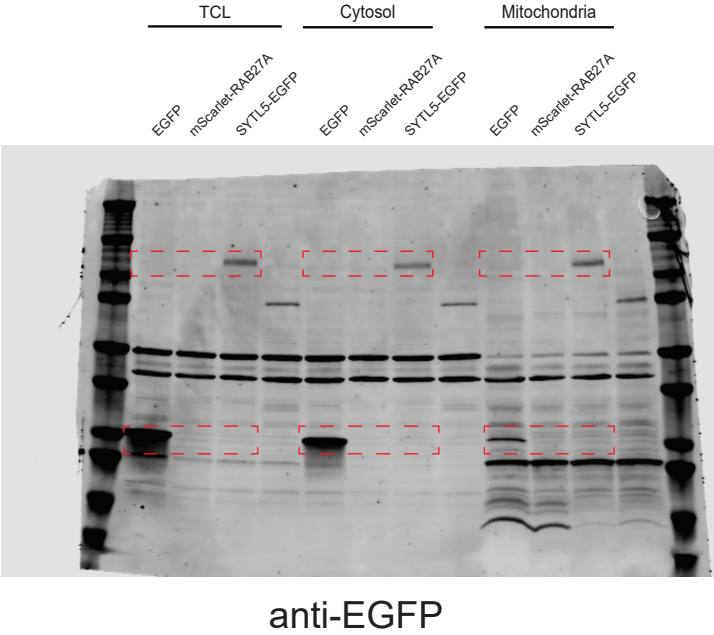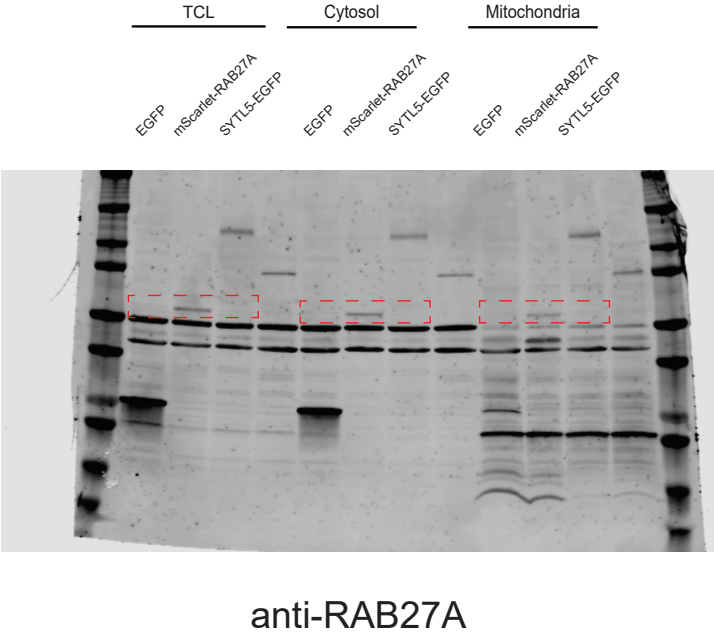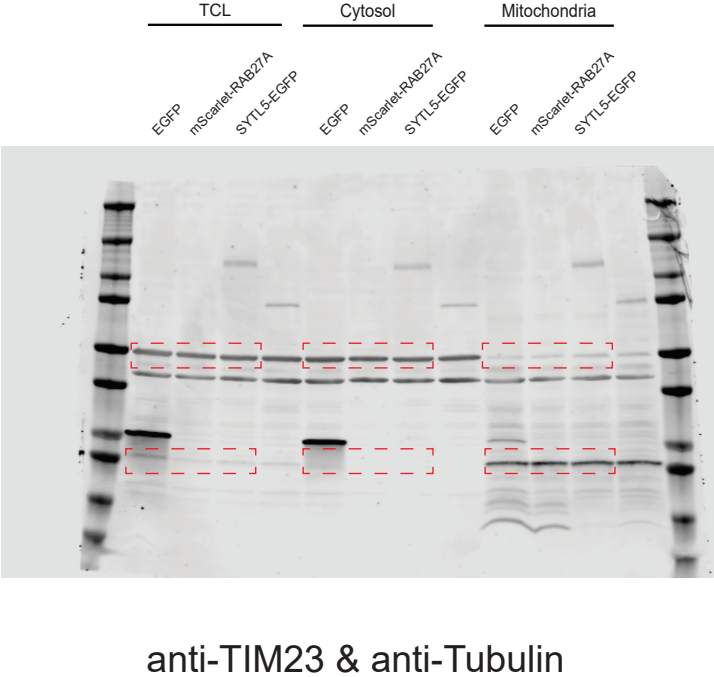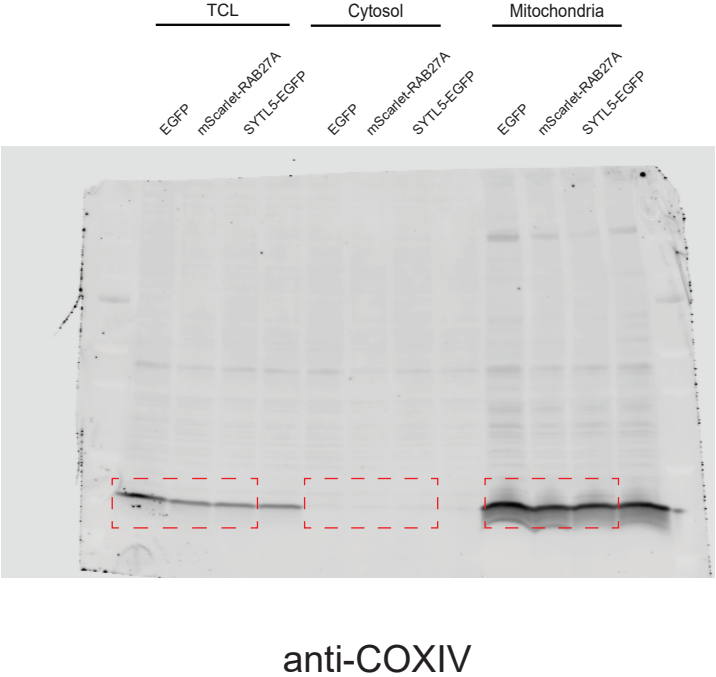

Supplement: Figure 2—figure supplement 1—source data 3. [file elife-105541-fig2-figsupp1-data3.zip › Figure 2 – figure supplement 1-Source Data 3/Figure 2 – figure supplement 1B-Source Data 5.pdf]

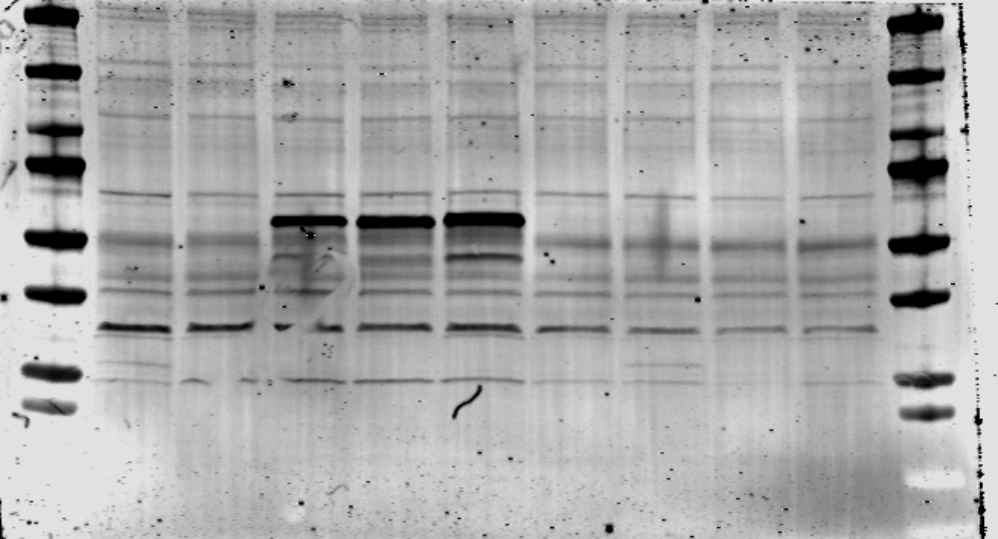

Supplement: Figure 2—figure supplement 2—source data 1. [file elife-105541-fig2-figsupp2-data1.zip › Figure 2 – figure supplement 2-Source Data 1/Figure 2 – figure supplement 2B-Source Data 1.png]

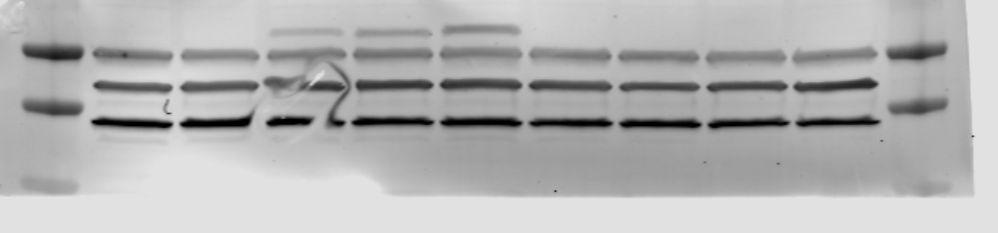

Supplement: Figure 2—figure supplement 2—source data 1. [file elife-105541-fig2-figsupp2-data1.zip › Figure 2 – figure supplement 2-Source Data 1/Figure 2 – figure supplement 2B-Source Data 2.png]

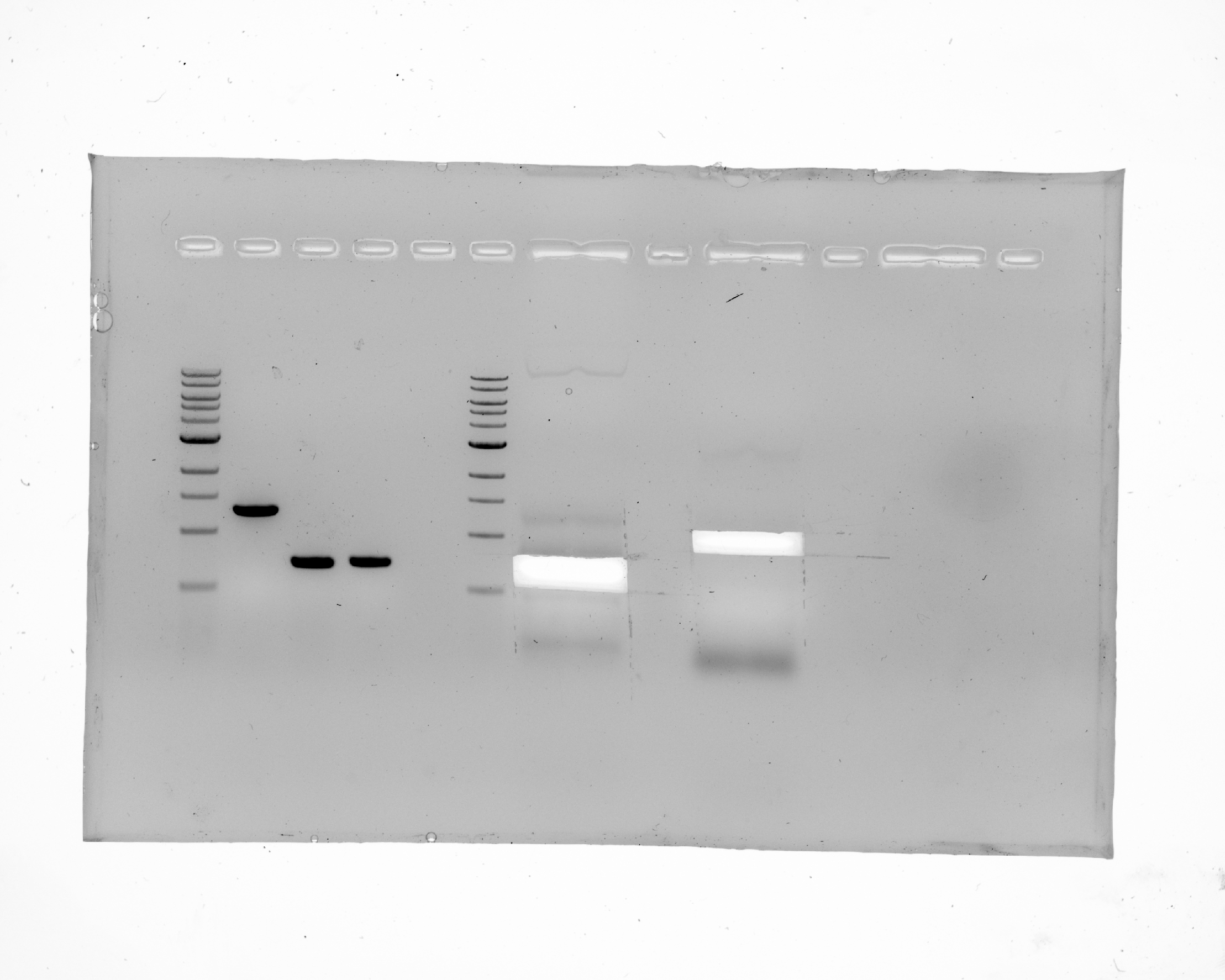

Supplement: Figure 2—figure supplement 2—source data 1. [file elife-105541-fig2-figsupp2-data1.zip › Figure 2 – figure supplement 2-Source Data 1/Figure 2 – figure supplement 2D-Source Data.jpg]

Figure 2 – figure supplement 2B

anti-RAB27A

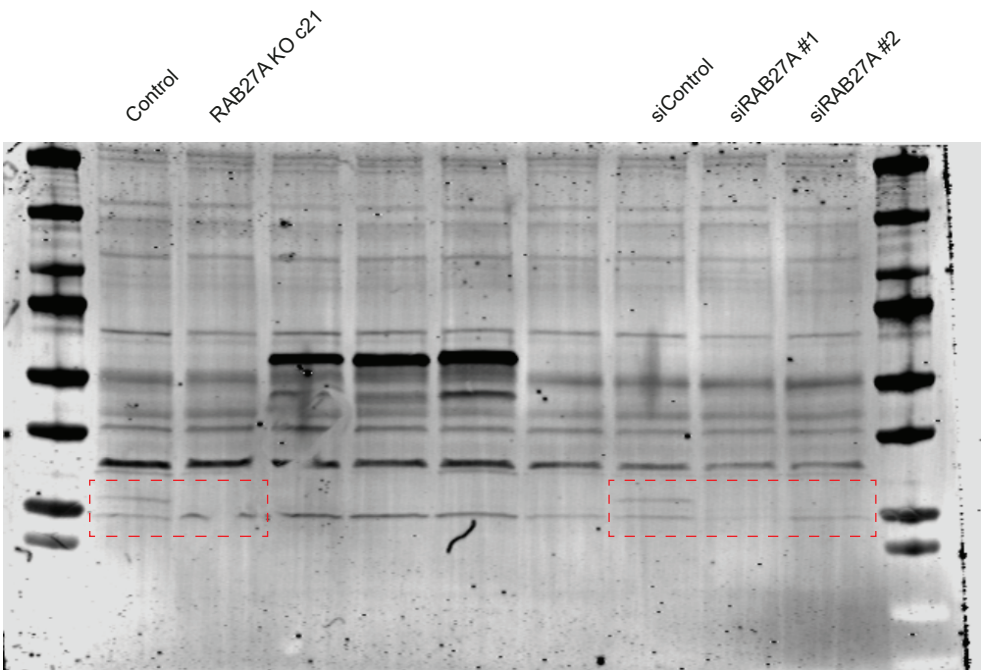

anti-Actin

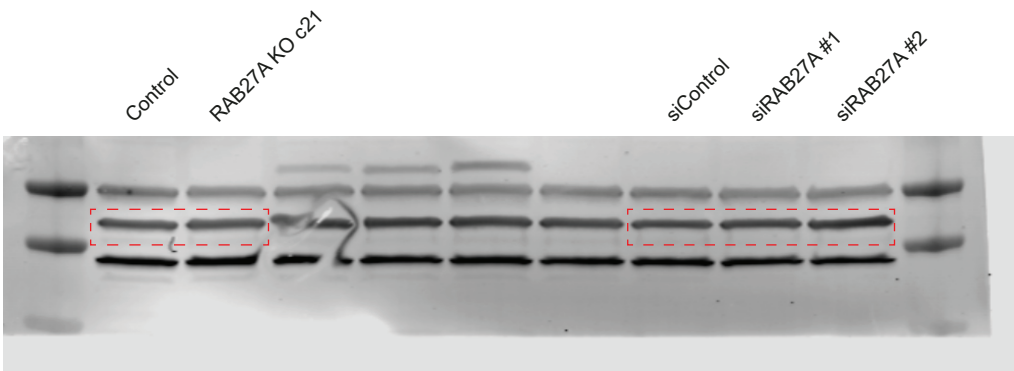

Figure 2 – figure supplement 2D

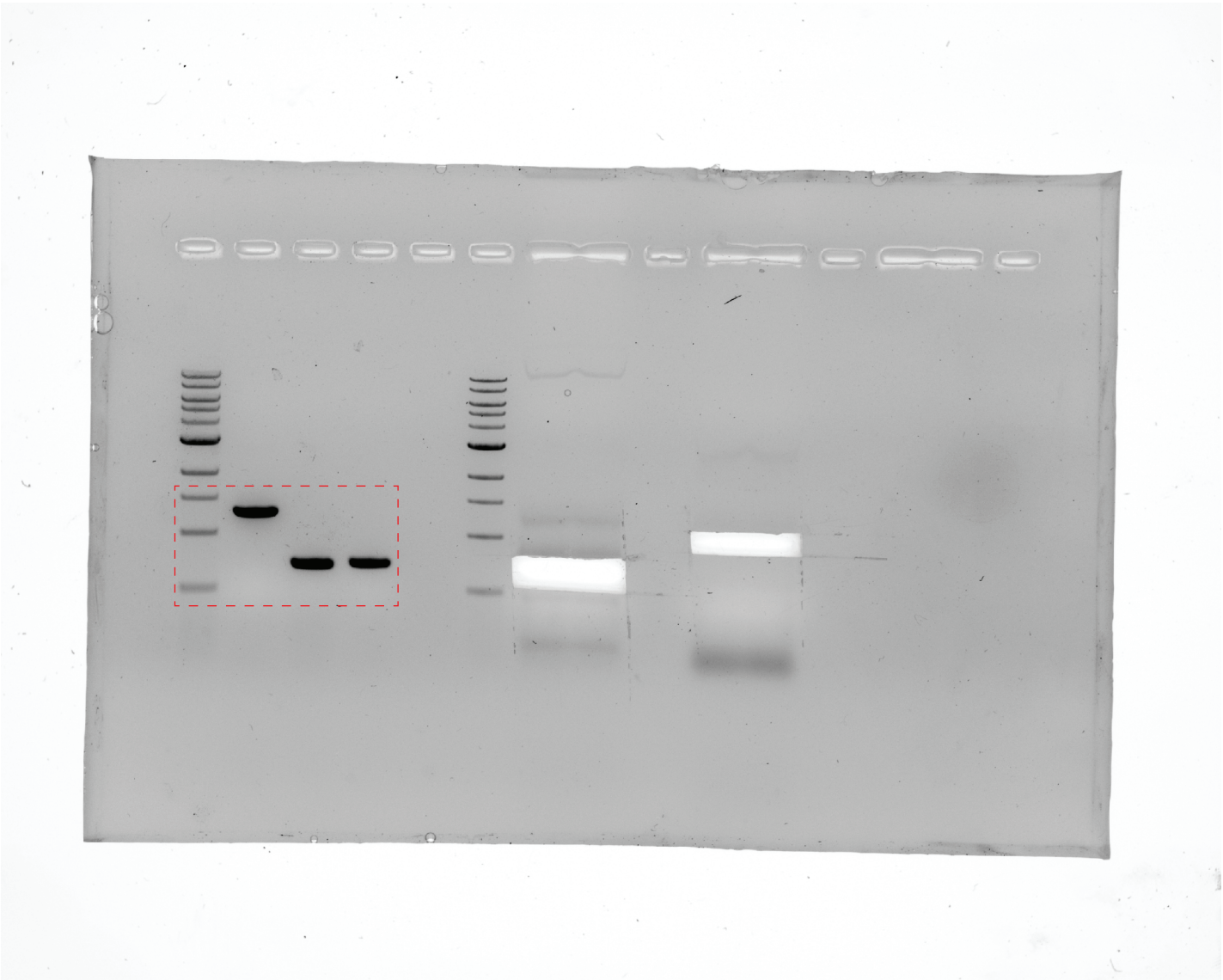

Supplement: Figure 2—figure supplement 2—source data 2. [file elife-105541-fig2-figsupp2-data2.zip › Figure 2 – figure supplement 2-Source Data 2/Figure 2 – figure supplement 2B-Source Data 3.pdf]

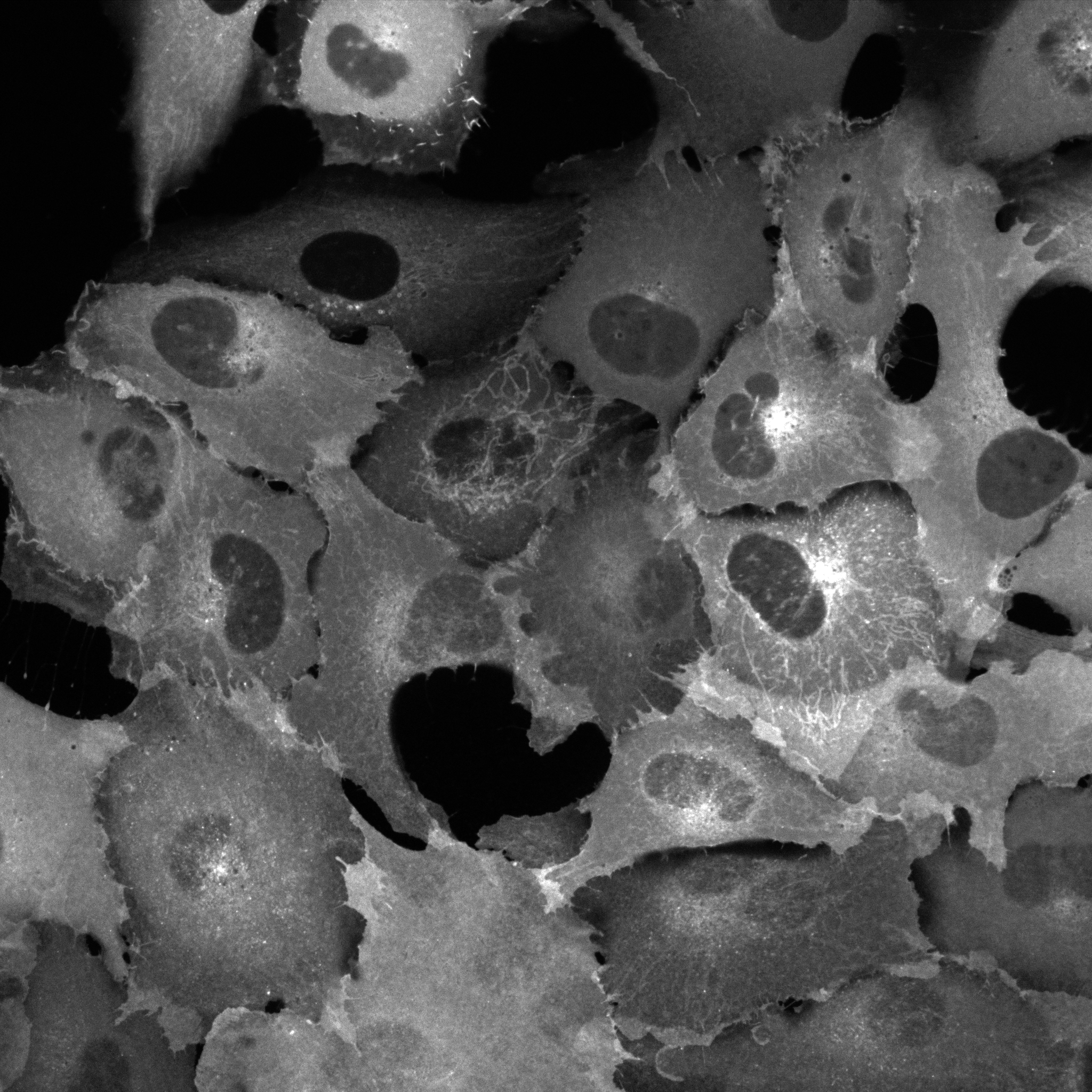

Supplement: Figure 4—source data 1. [file elife-105541-fig4-data1.zip › Figure 4-Source Data 1.1/Figure 4A-Source Data 1.tif]

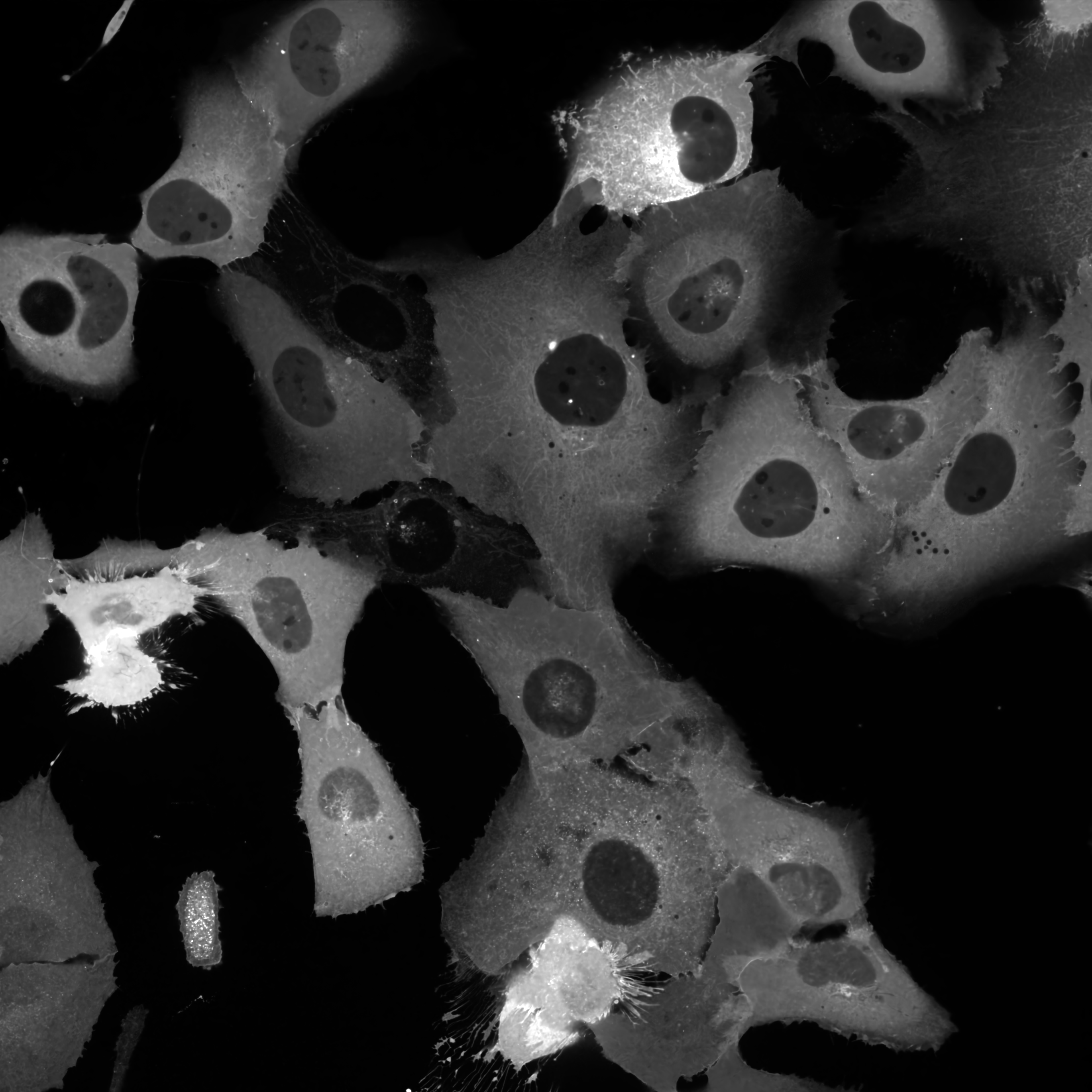

Supplement: Figure 4—source data 1. [file elife-105541-fig4-data1.zip › Figure 4-Source Data 1.1/Figure 4A-Source Data 2.tif]

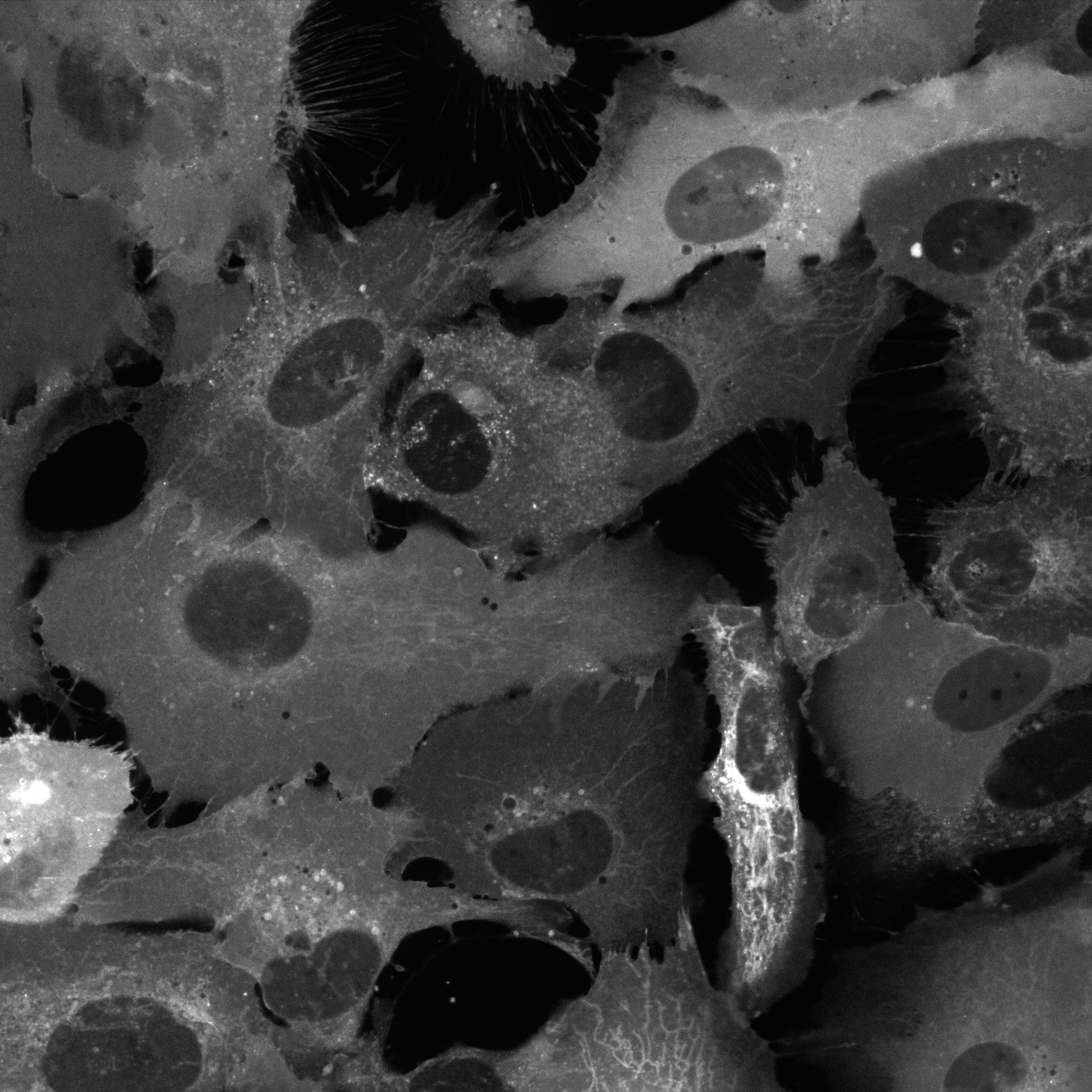

Supplement: Figure 4—source data 1. [file elife-105541-fig4-data1.zip › Figure 4-Source Data 1.1/Figure 4A-Source Data 3.tif]

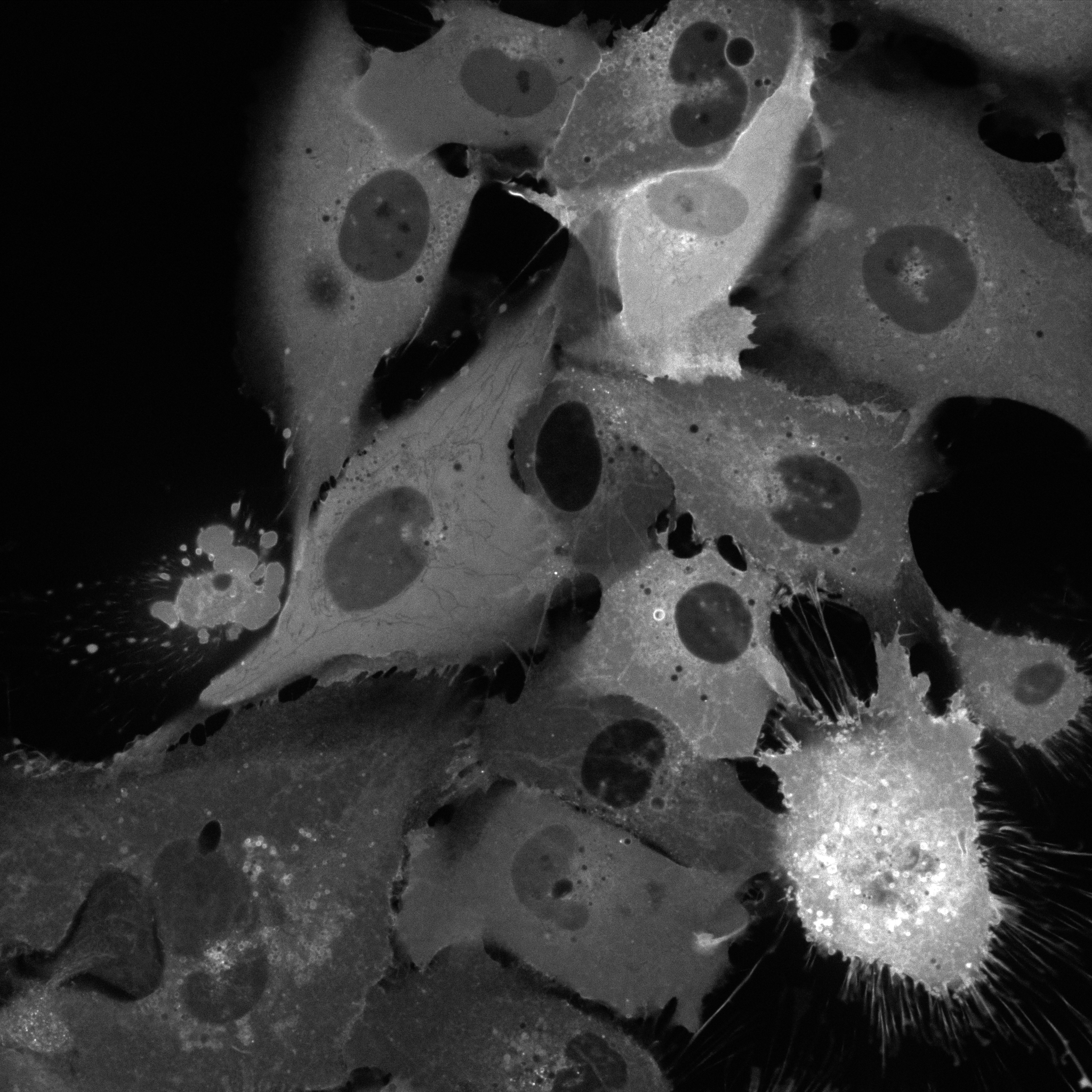

Supplement: Figure 4—source data 1. [file elife-105541-fig4-data1.zip › Figure 4-Source Data 1.1/Figure 4A-Source Data 4.tif]

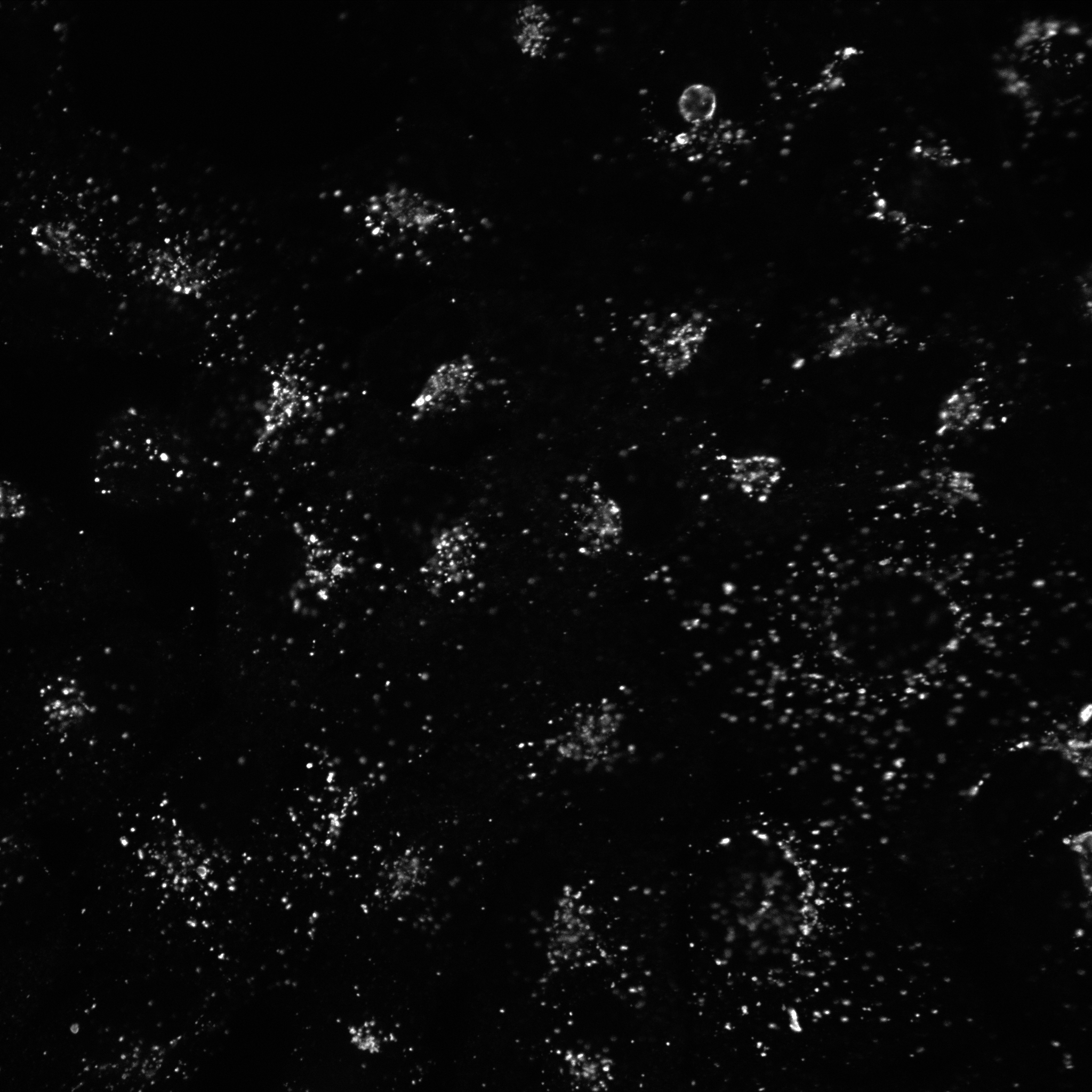

Supplement: Figure 4—source data 2. [file elife-105541-fig4-data2.zip › Figure 4-Source Data 1.2/Figure 4B-Source Data 1.tif]

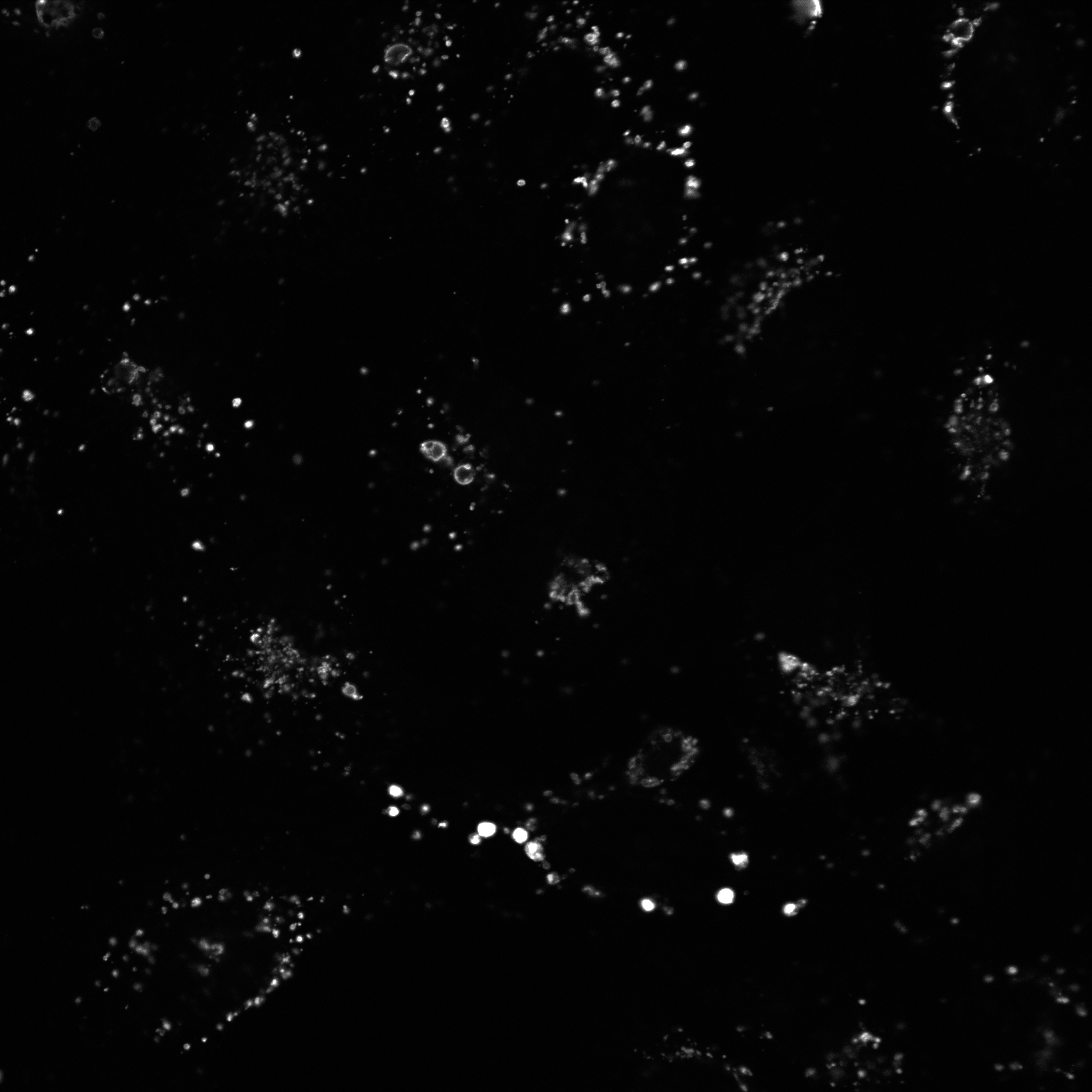

Supplement: Figure 4—source data 2. [file elife-105541-fig4-data2.zip › Figure 4-Source Data 1.2/Figure 4B-Source Data 2.tif]

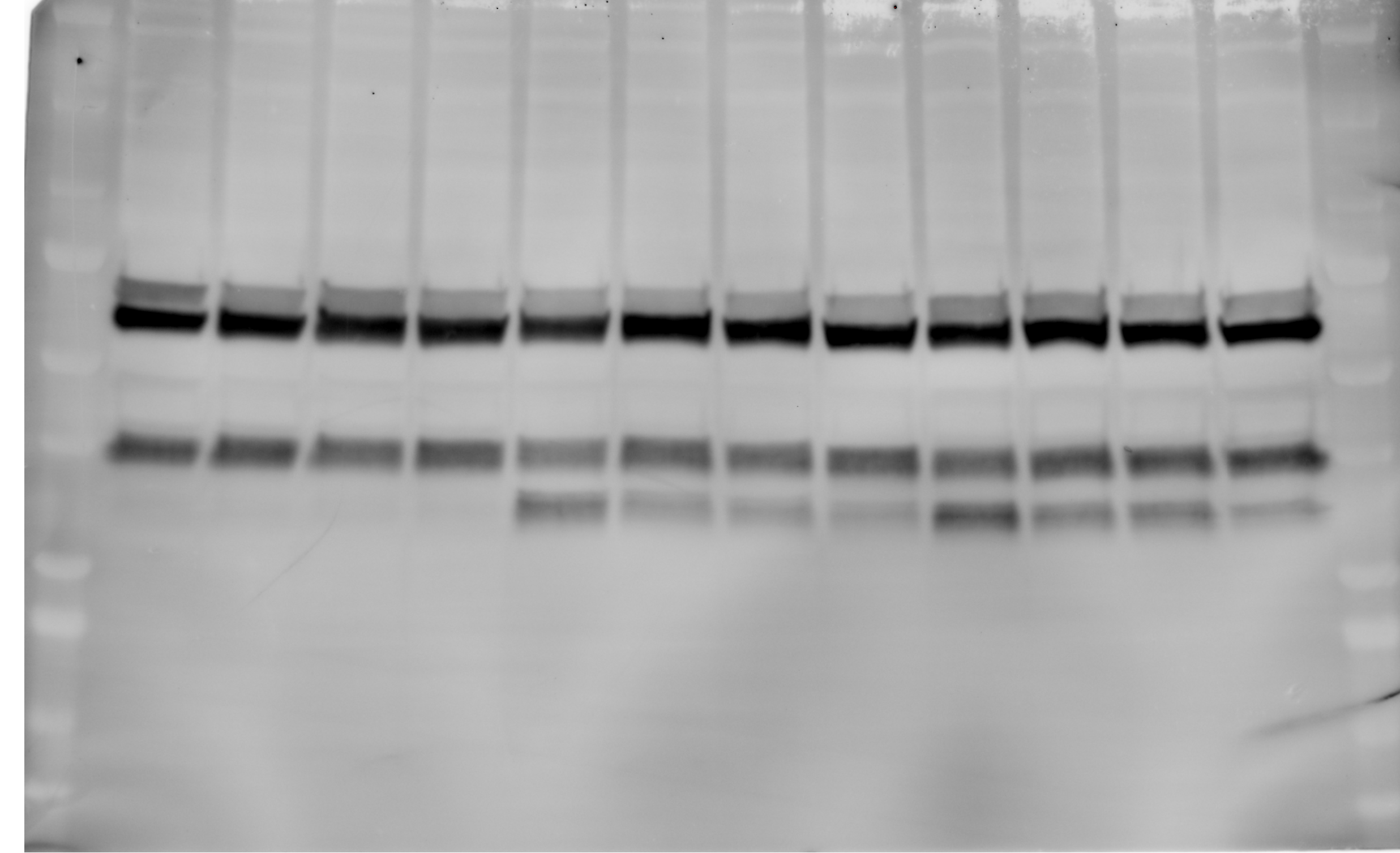

Supplement: Figure 4—source data 3. [file elife-105541-fig4-data3.zip › Figure 4-Source Data 2/Figure 4C-Source Data 1.tif]

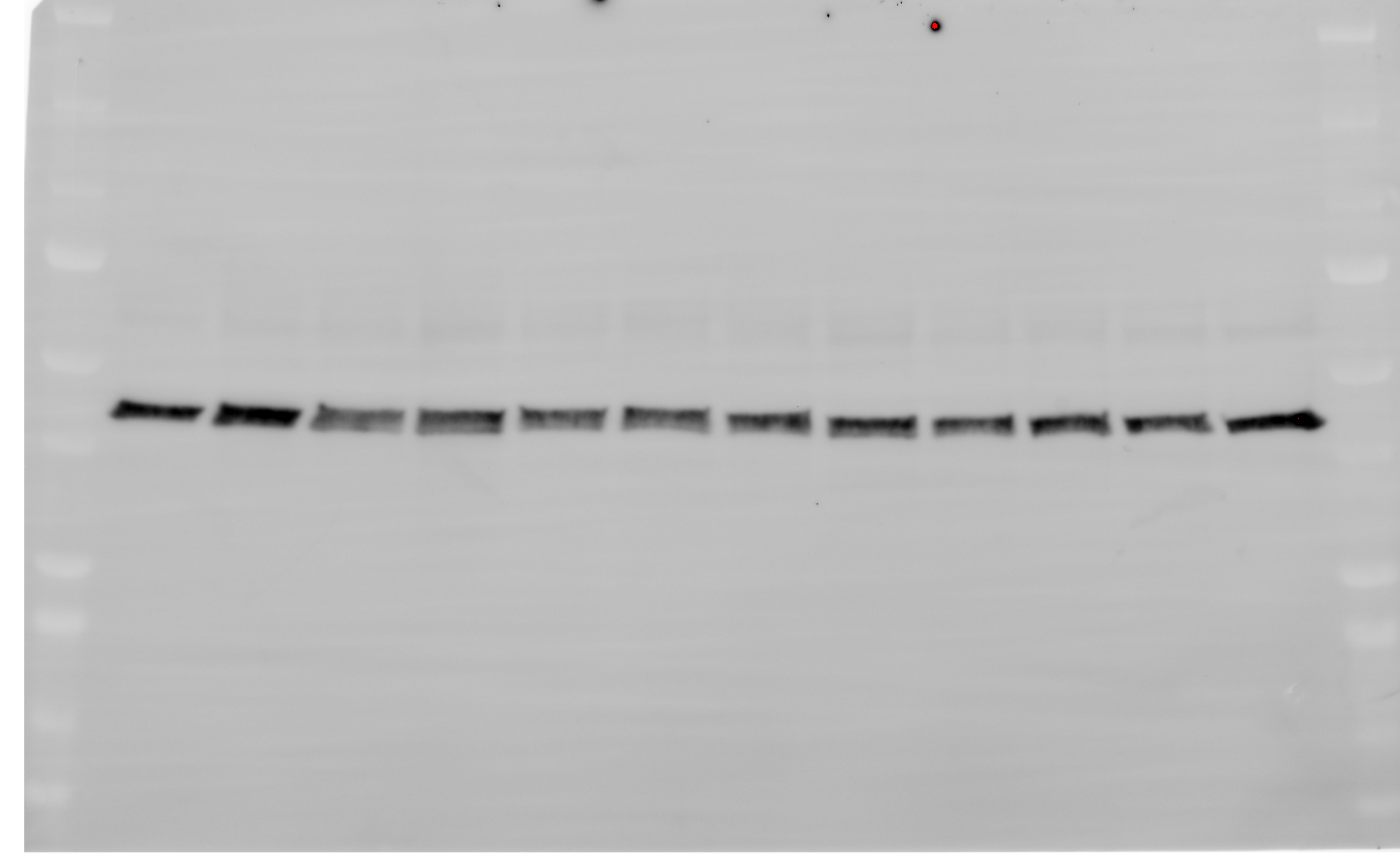

Supplement: Figure 4—source data 3. [file elife-105541-fig4-data3.zip › Figure 4-Source Data 2/Figure 4C-Source Data 2.tif]

Figure 4C

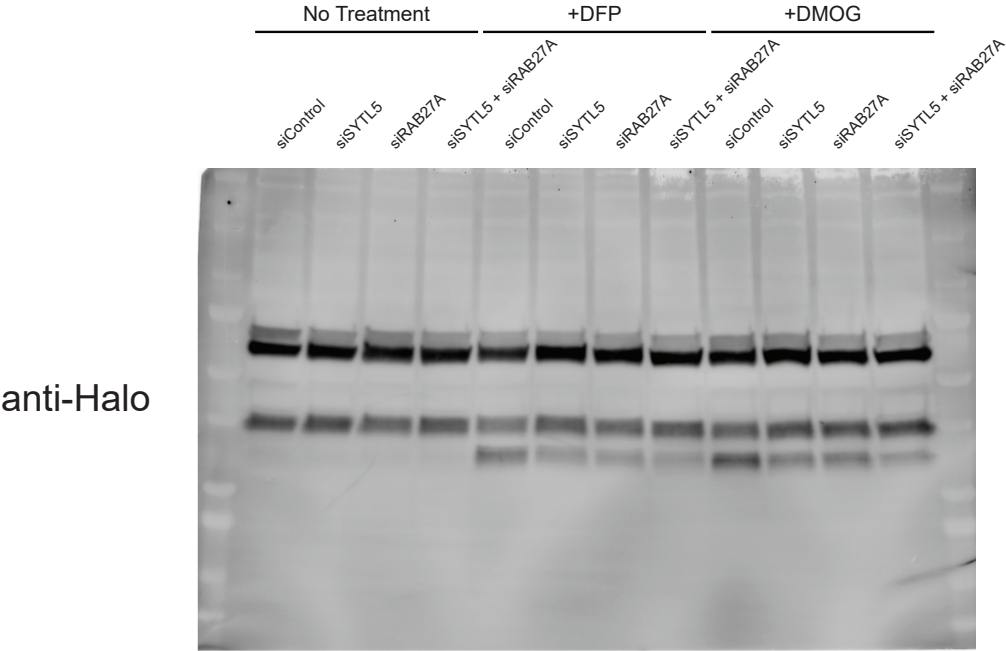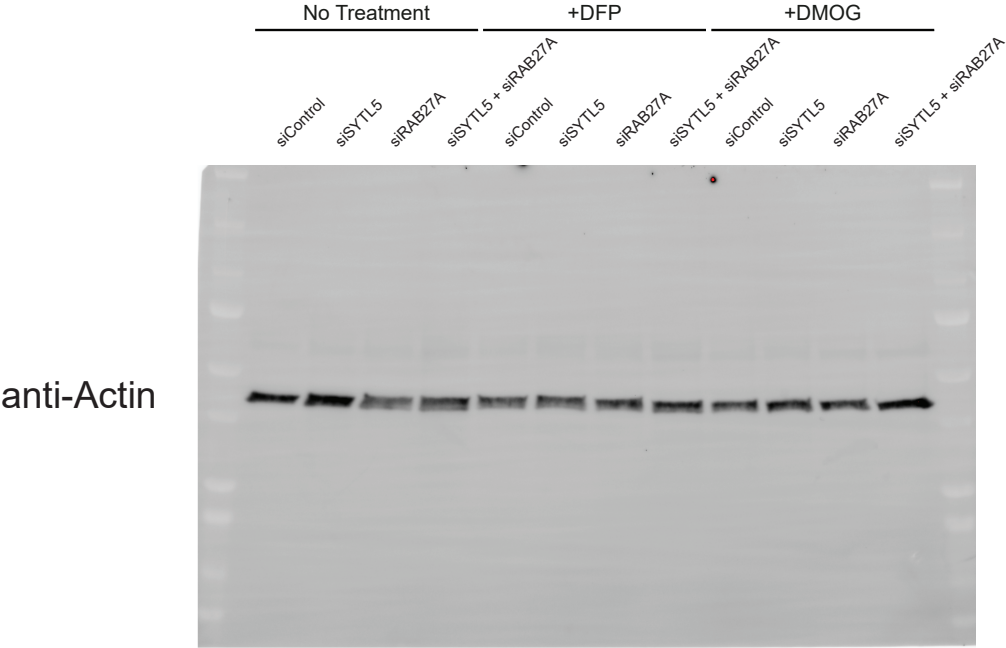

Supplement: Figure 4—source data 4. [file elife-105541-fig4-data4.zip › Figure 4-Source Data 3/Figure 4C-Source Data 3.pdf]

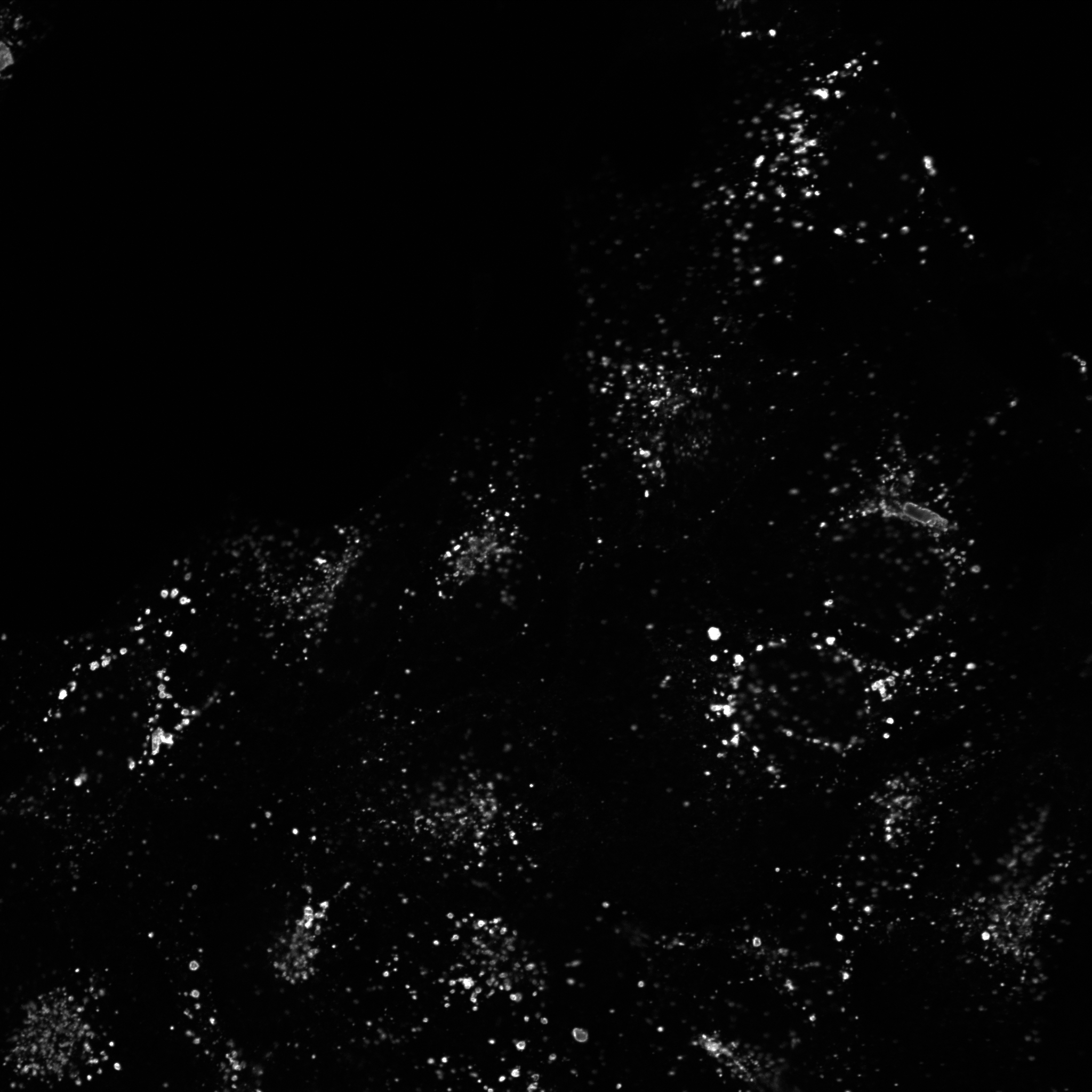

Supplement: Figure 4—figure supplement 1—source data 1. [file elife-105541-fig4-figsupp1-data1.zip › Figure 4 – figure supplement 1-Source Data 1.1/Figure 4 – figure supplement 1A-Source Data 1.tif]

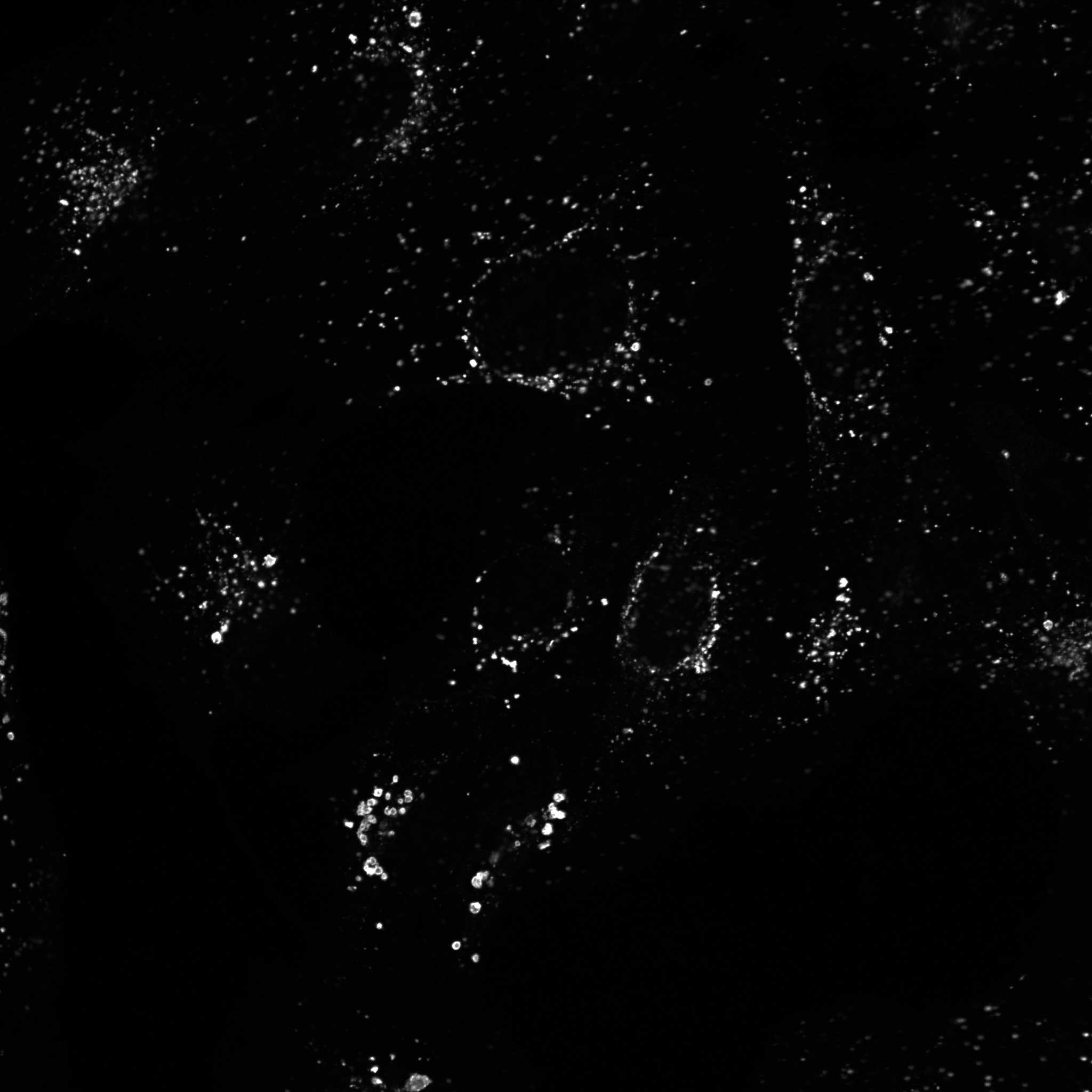

Supplement: Figure 4—figure supplement 1—source data 1. [file elife-105541-fig4-figsupp1-data1.zip › Figure 4 – figure supplement 1-Source Data 1.1/Figure 4 – figure supplement 1A-Source Data 2.tif]

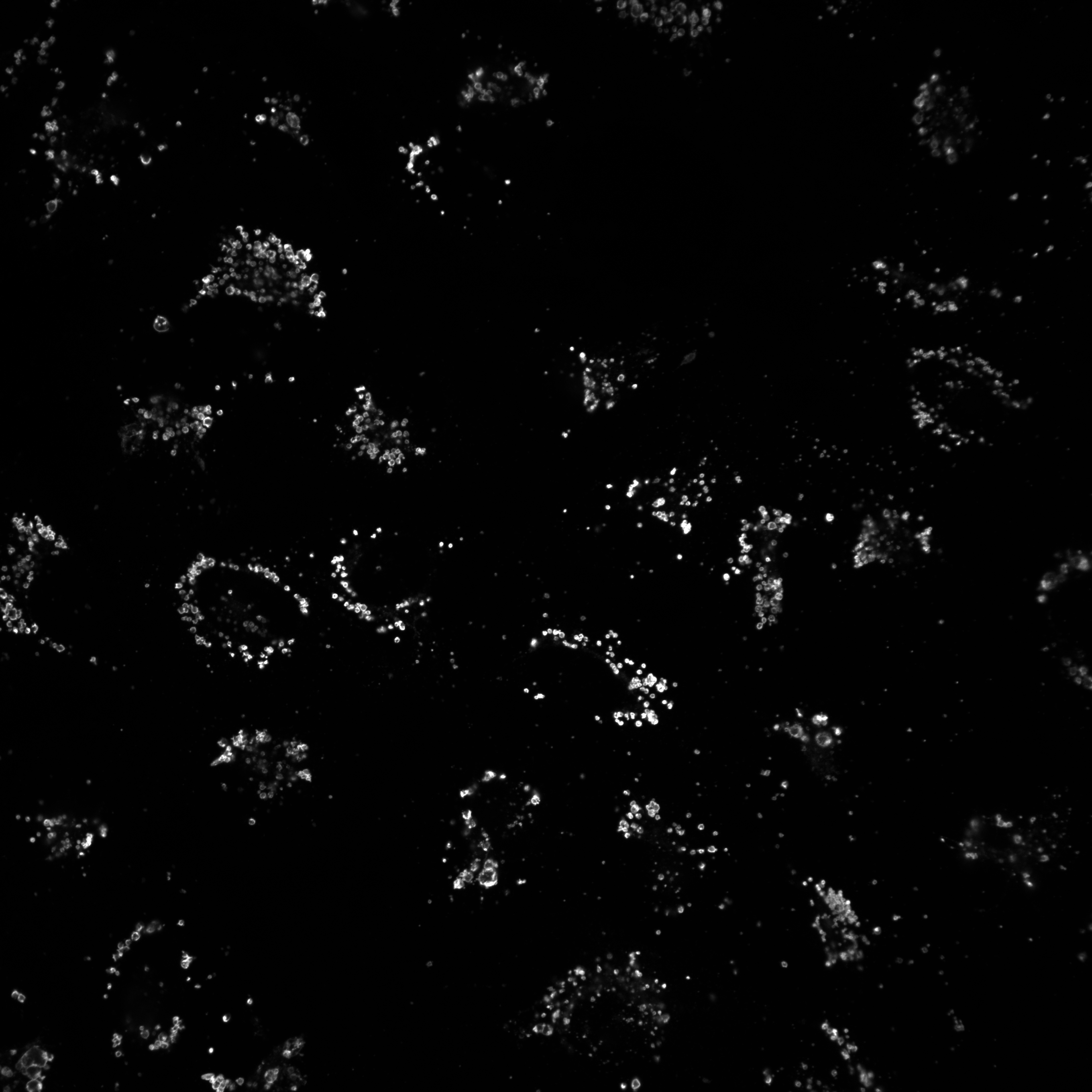

Supplement: Figure 4—figure supplement 1—source data 2. [file elife-105541-fig4-figsupp1-data2.zip › Figure 4 – figure supplement 1-Source Data 1.2/Figure 4 – figure supplement 1B-Source Data 1.tif]

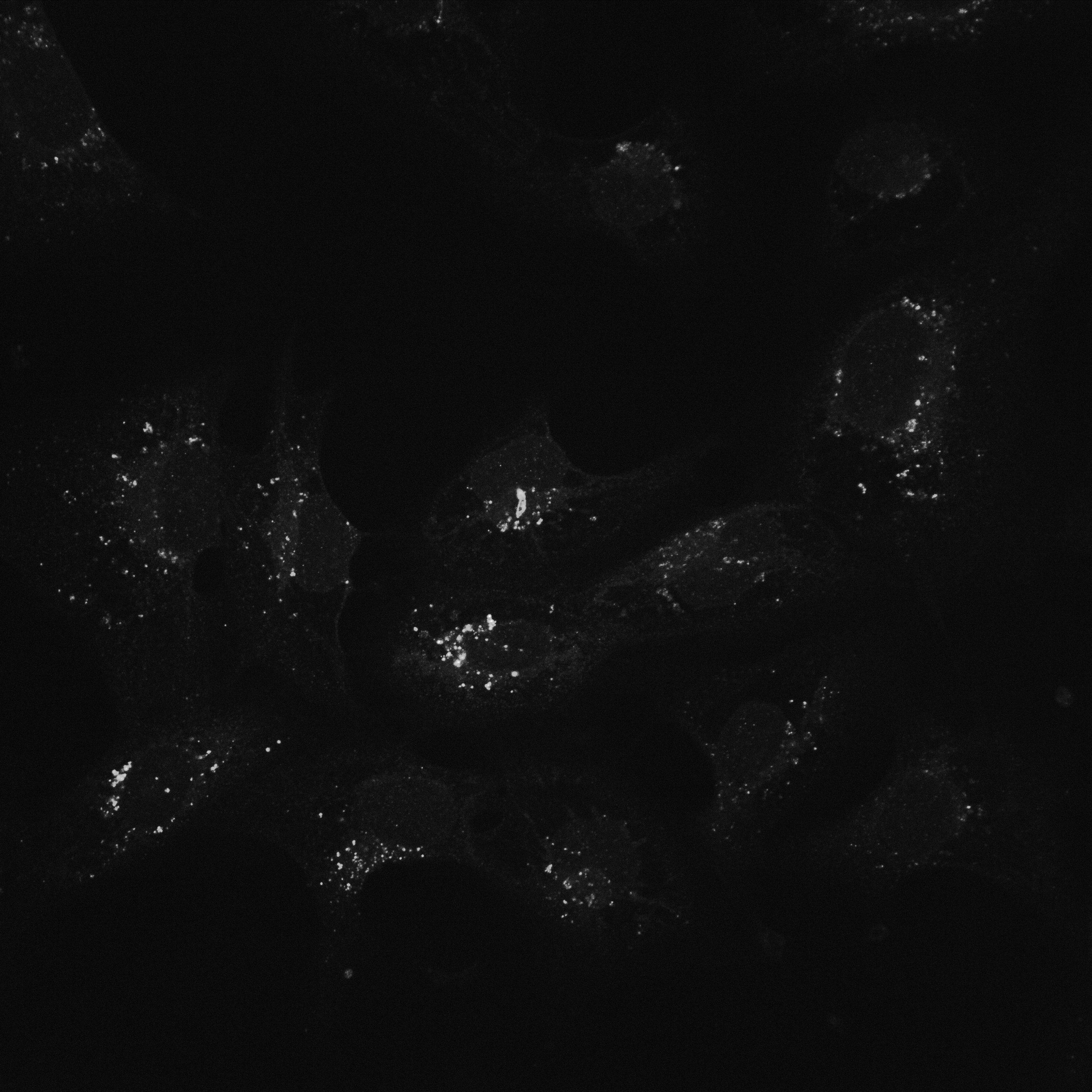

Supplement: Figure 4—figure supplement 1—source data 2. [file elife-105541-fig4-figsupp1-data2.zip › Figure 4 – figure supplement 1-Source Data 1.2/Figure 4 – figure supplement 1B-Source Data 2.tif]

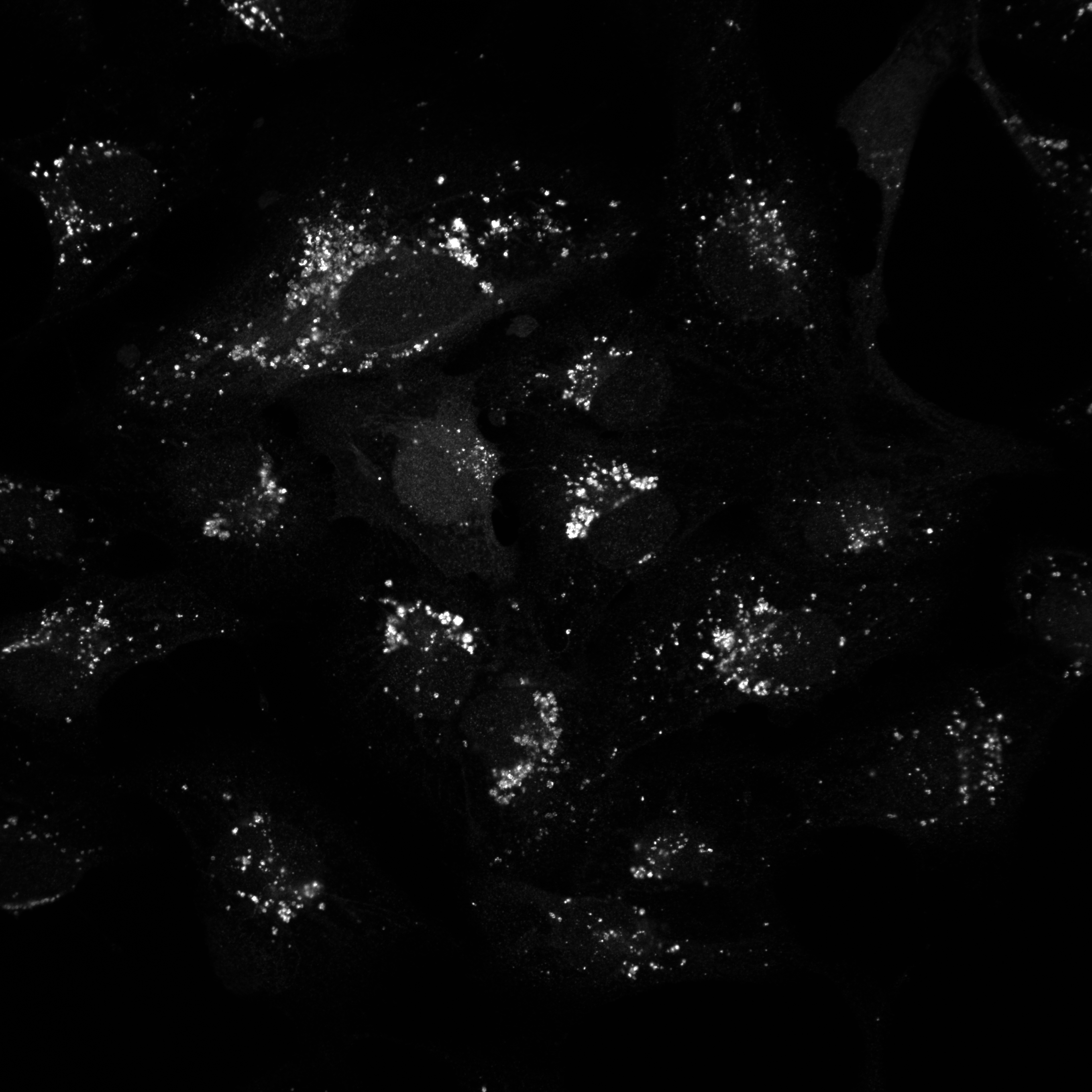

Supplement: Figure 4—figure supplement 1—source data 3. [file elife-105541-fig4-figsupp1-data3.zip › Figure 4 – figure supplement 1-Source Data 1.3/Figure 4 – figure supplement 1B-Source Data 3.tif]

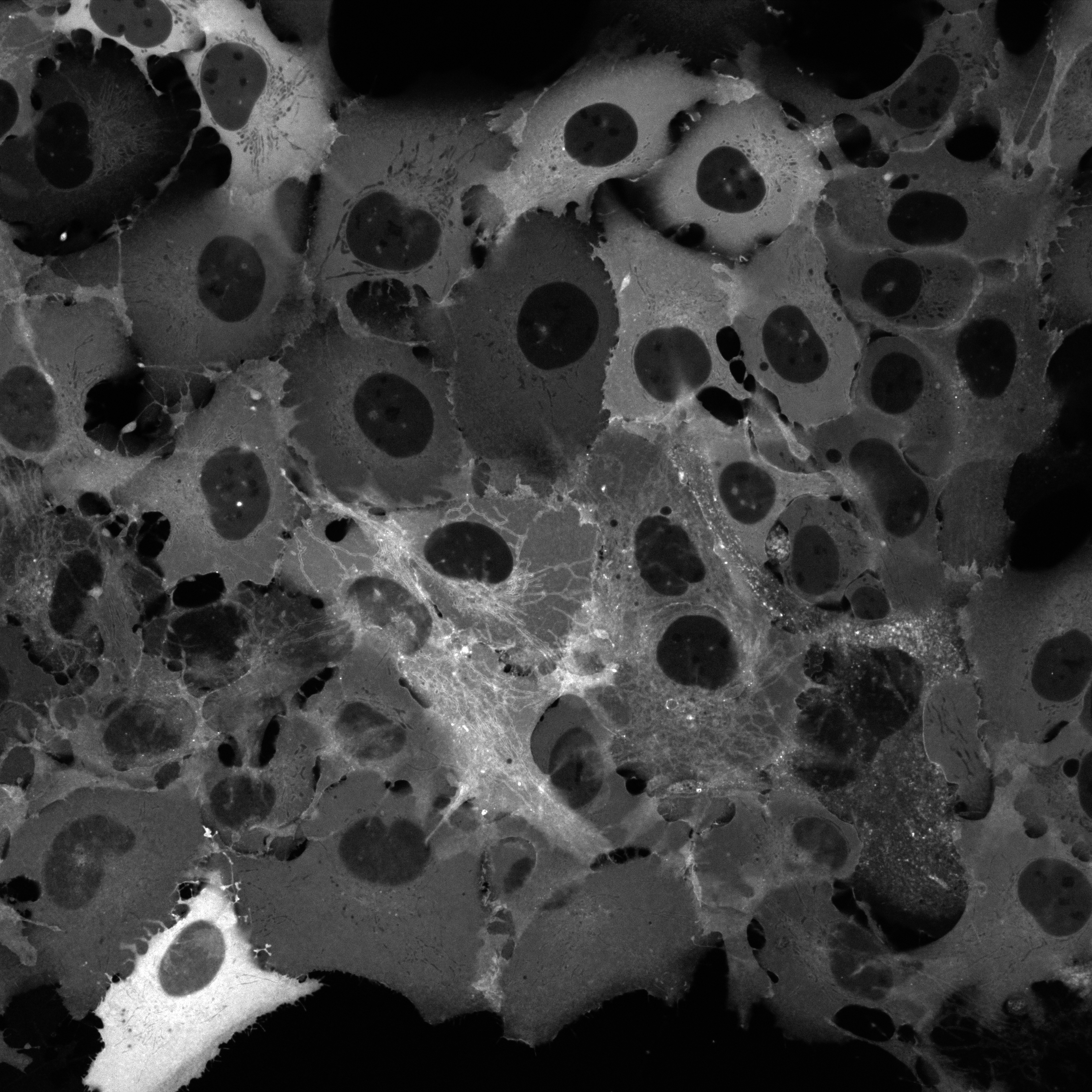

Supplement: Figure 4—figure supplement 1—source data 3. [file elife-105541-fig4-figsupp1-data3.zip › Figure 4 – figure supplement 1-Source Data 1.3/Figure 4 – figure supplement 1C-Source Data 1.tif]

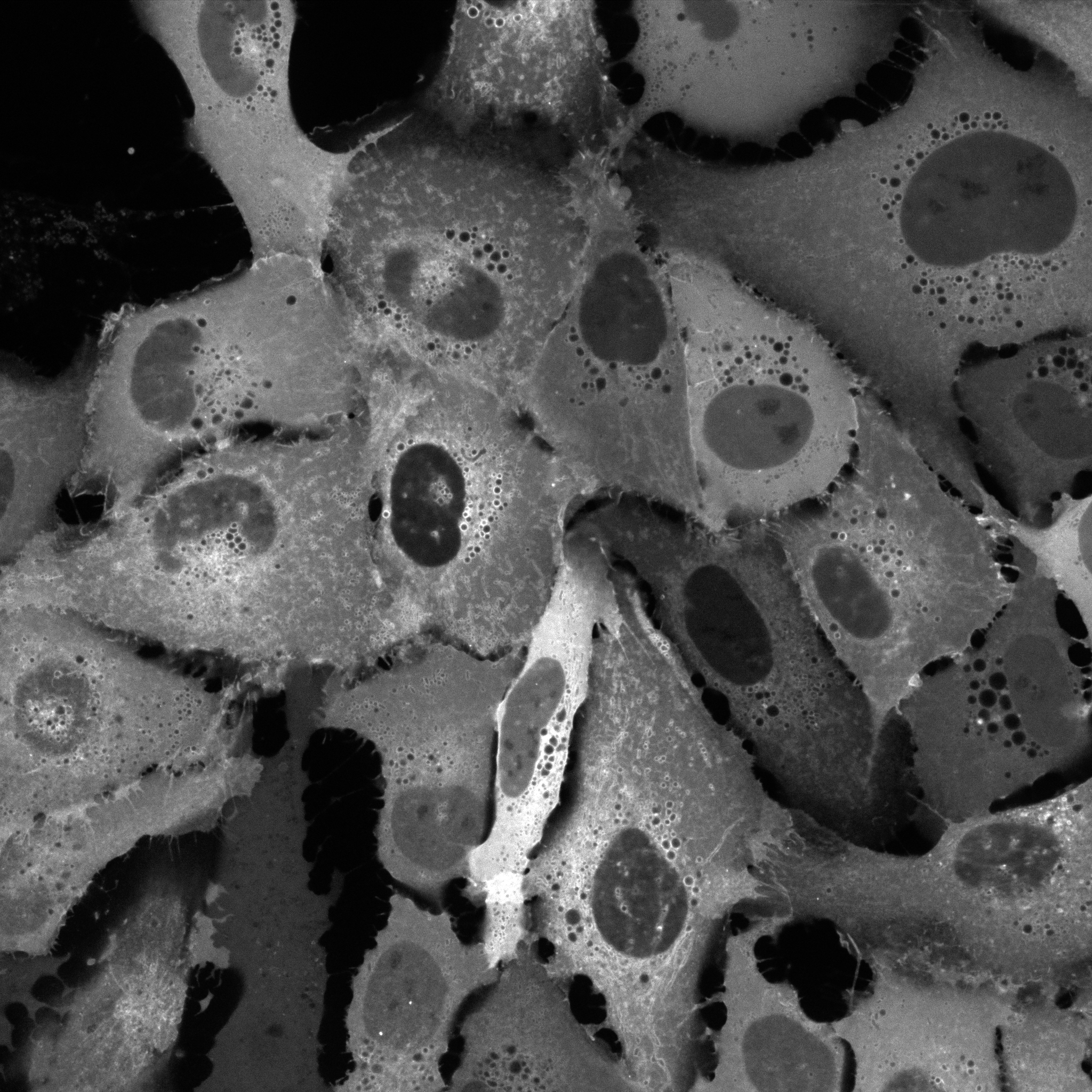

Supplement: Figure 4—figure supplement 1—source data 3. [file elife-105541-fig4-figsupp1-data3.zip › Figure 4 – figure supplement 1-Source Data 1.3/Figure 4 – figure supplement 1C-Source Data 2.tif]

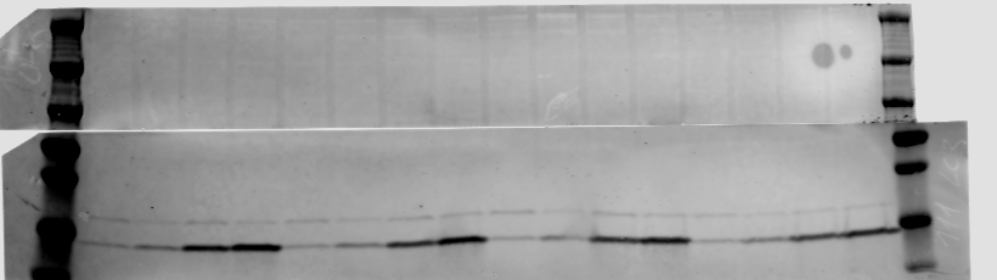

Supplement: Figure 4—figure supplement 2—source data 1. [file elife-105541-fig4-figsupp2-data1.zip › Figure 4 – figure supplement 2-Source Data 1/Figure 4 – figure supplement 2F-Source Data 1.png]

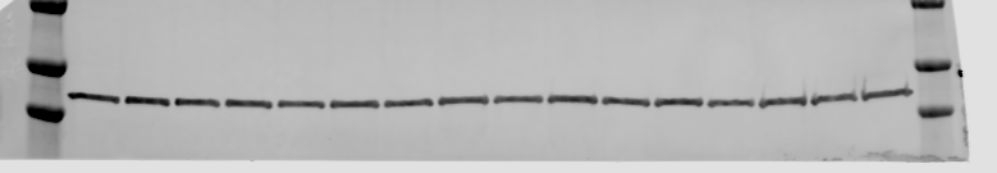

Supplement: Figure 4—figure supplement 2—source data 1. [file elife-105541-fig4-figsupp2-data1.zip › Figure 4 – figure supplement 2-Source Data 1/Figure 4 – figure supplement 2F-Source Data 2.png]

Figure 4 – figure supplement 2F

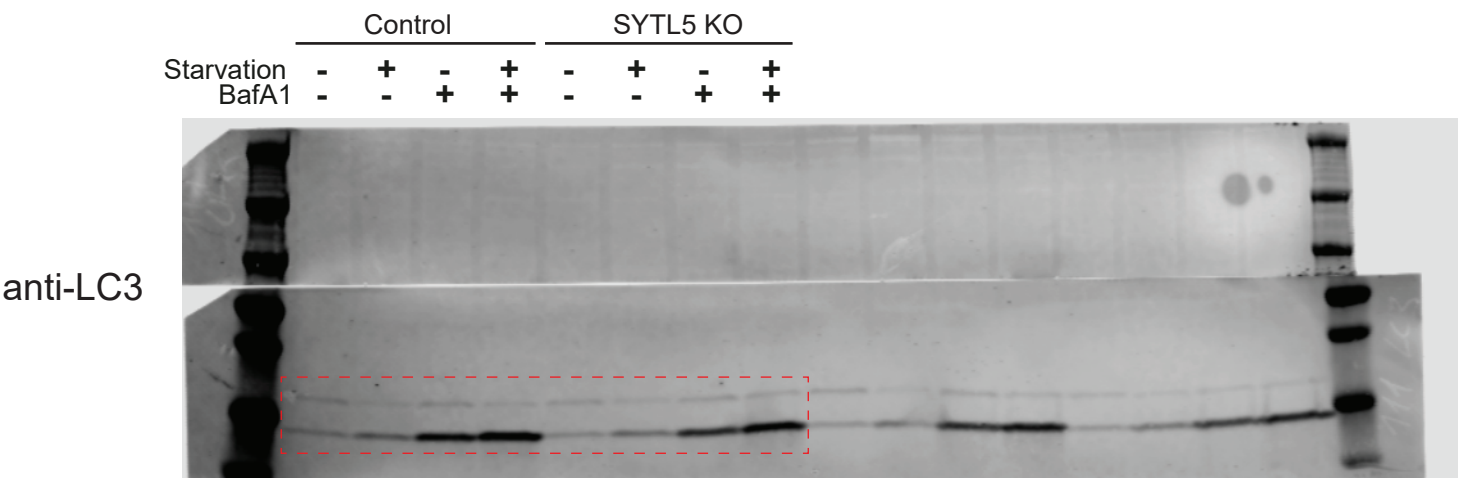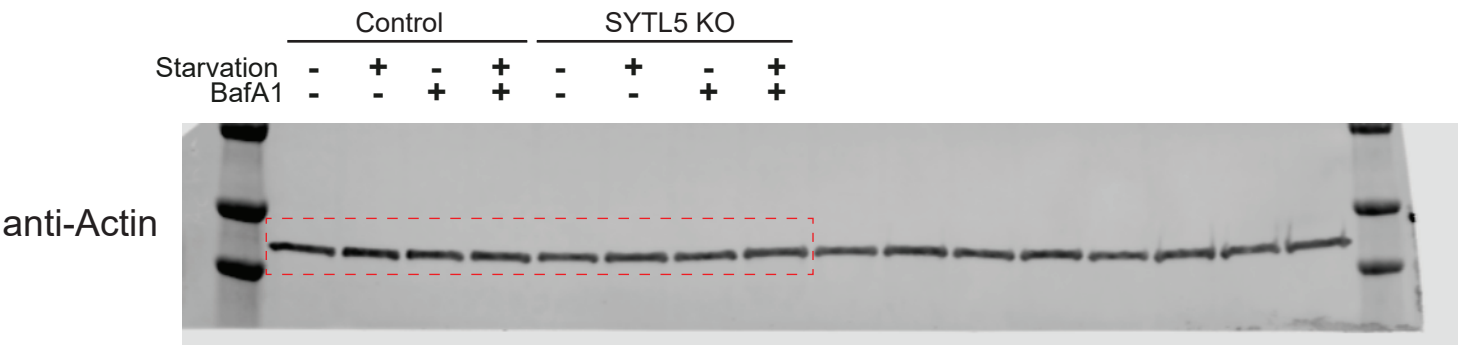

Supplement: Figure 4—figure supplement 2—source data 2. [file elife-105541-fig4-figsupp2-data2.zip › Figure 4 – figure supplement 2-Source Data 2/Figure 4 – figure supplement 2F-Source Data 3.pdf]

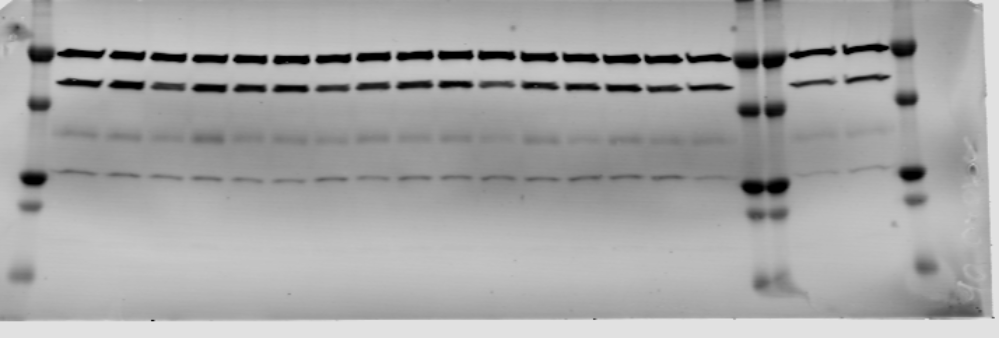

Supplement: Figure 5—source data 1. [file elife-105541-fig5-data1.zip › Figure 5-Source Data 1/Figure 5E-Source Data 1.png]

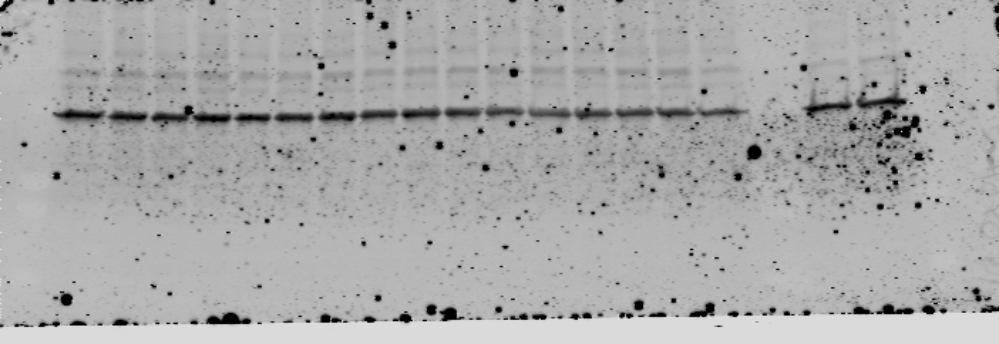

Supplement: Figure 5—source data 1. [file elife-105541-fig5-data1.zip › Figure 5-Source Data 1/Figure 5E-Source Data 2.png]

Figure 5E

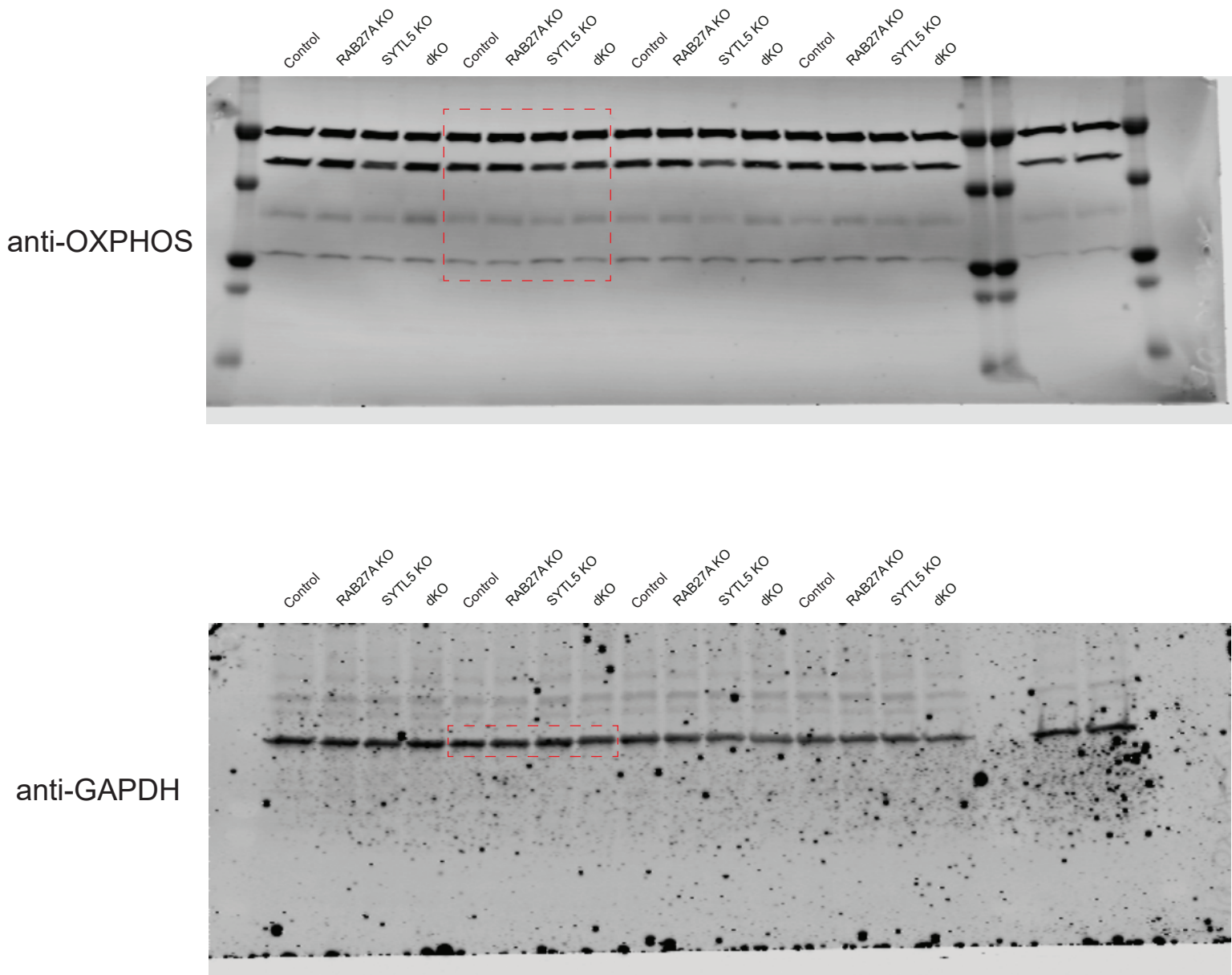

Supplement: Figure 5—source data 2. [file elife-105541-fig5-data2.zip › Figure 5-Source Data 2/Figure 5E-Source Data 3.pdf]
