# Supplementary material for: The RAB27A effector SYTL5 regulates mitophagy and mitochondrial metabolism
Source: eLife. 2025 Nov 26;14:RP105541. doi: 10.7554/eLife.105541 (PMC12656530; doi:10.7554/eLife.105541)

Figure 1F

PIP strip : Full-length

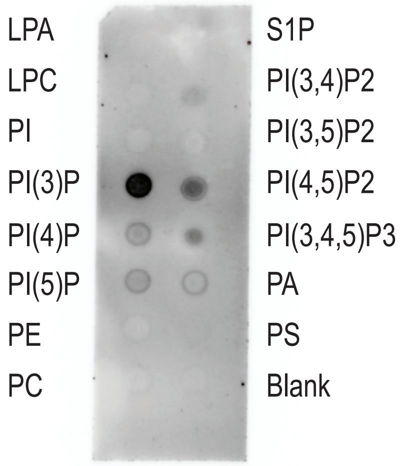

Figure 1F

PIP strip : ΔSHD

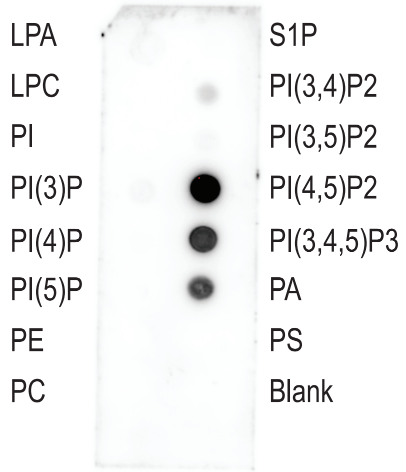

Figure 1F

PIP strip : ΔC2AB

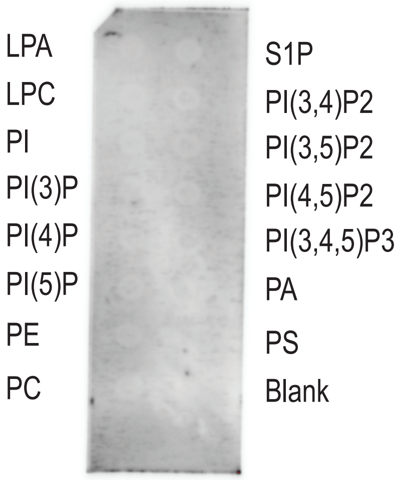

Figure 1F

PIP strip : Control

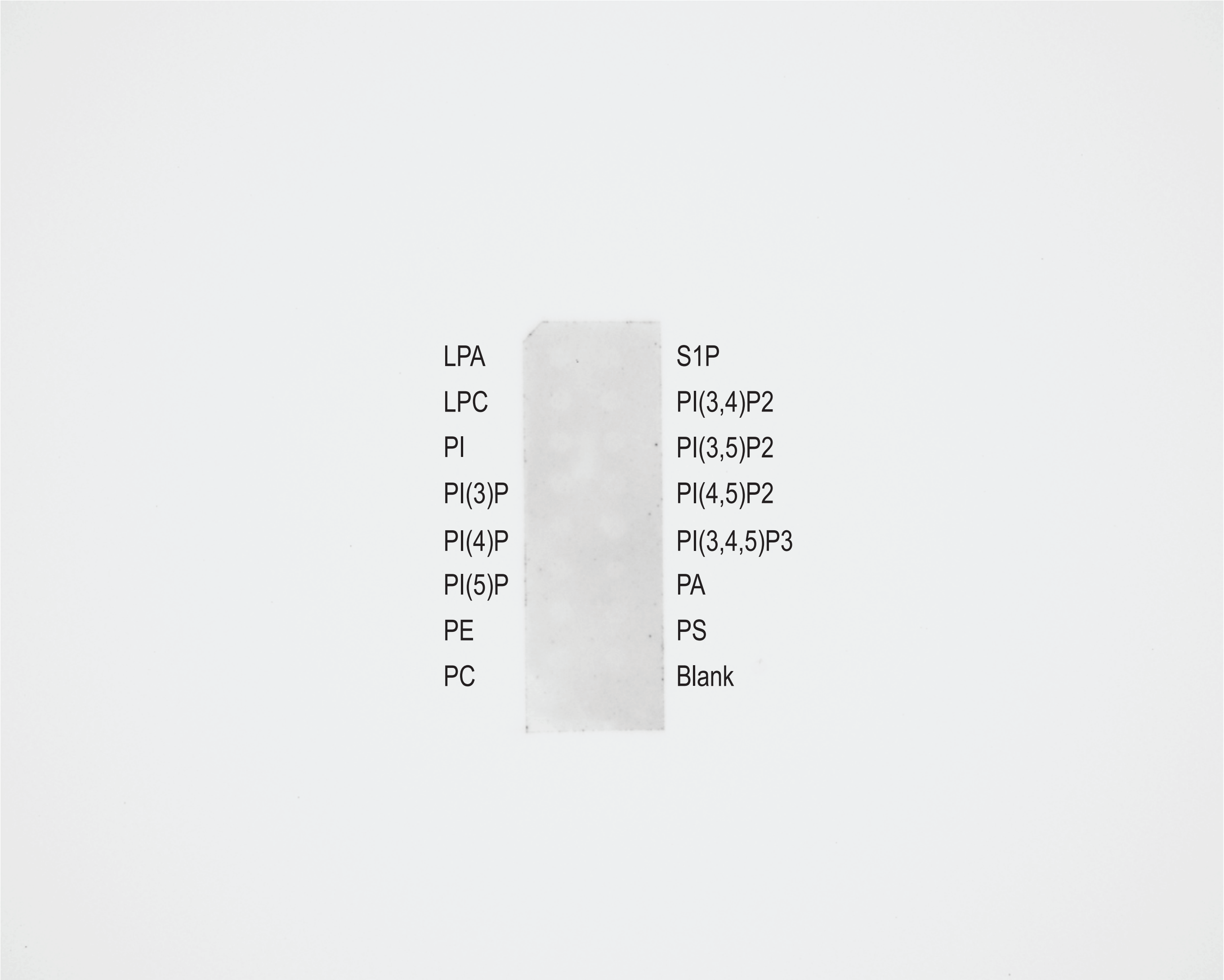

Figure 1F

Lipid membrane strip : Full-length

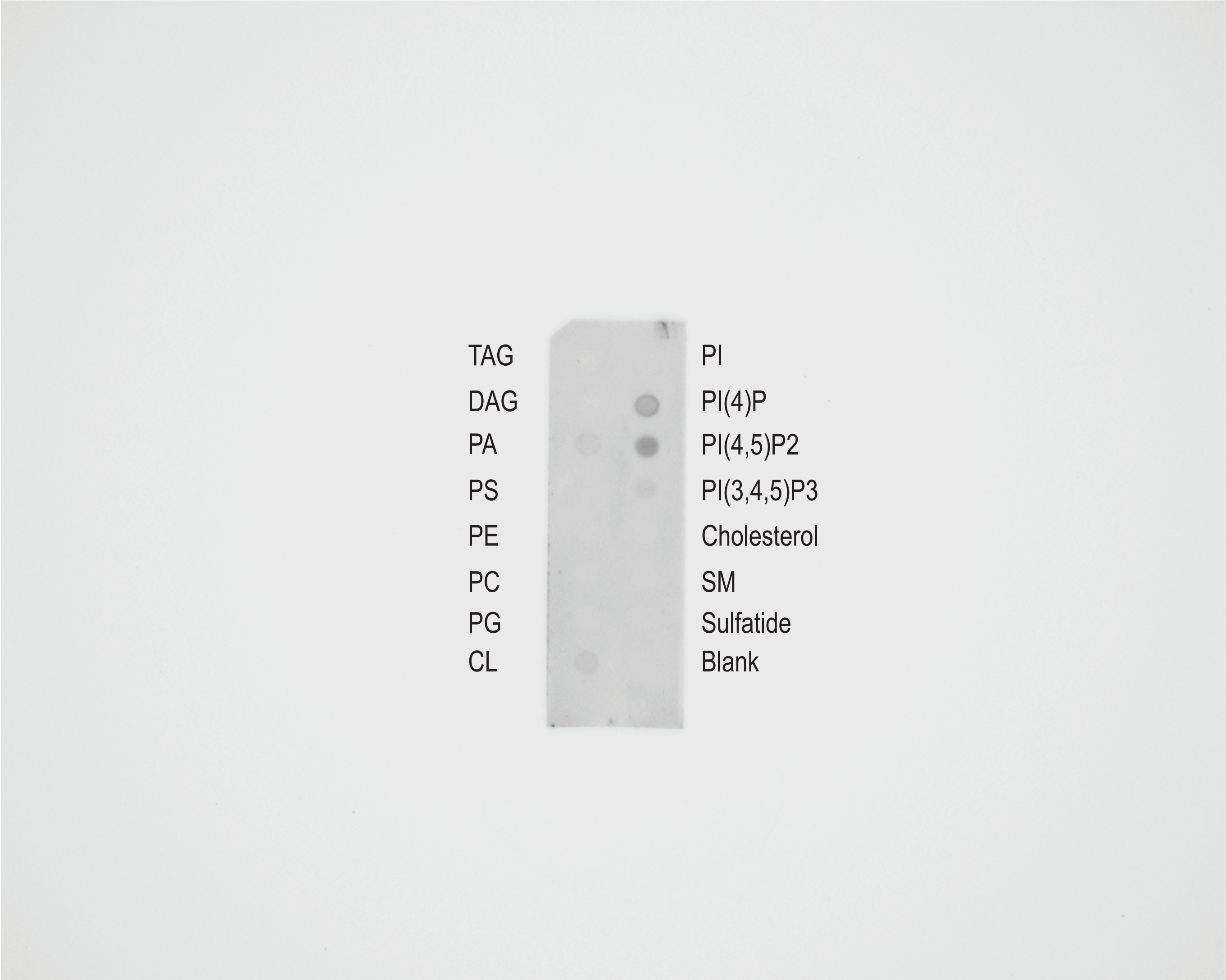

Figure 1F

Lipid membrane strip : ΔSHD

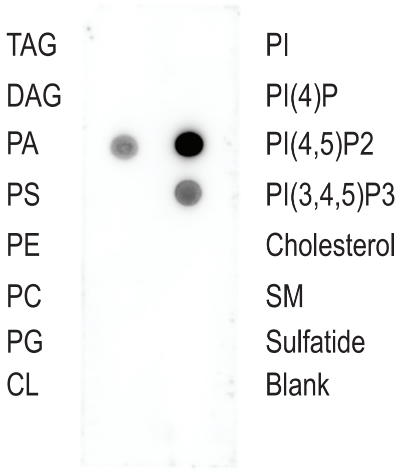

Figure 1F

Lipid membrane strip : ΔC2AB

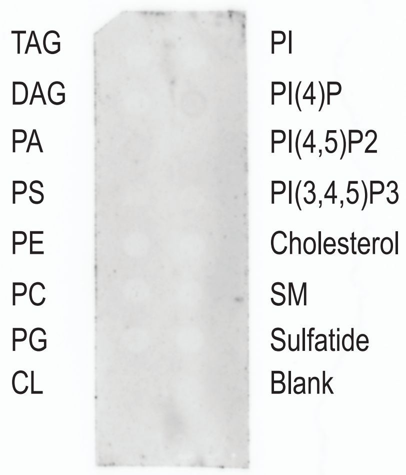

Figure 1F

Lipid membrane strip : Control

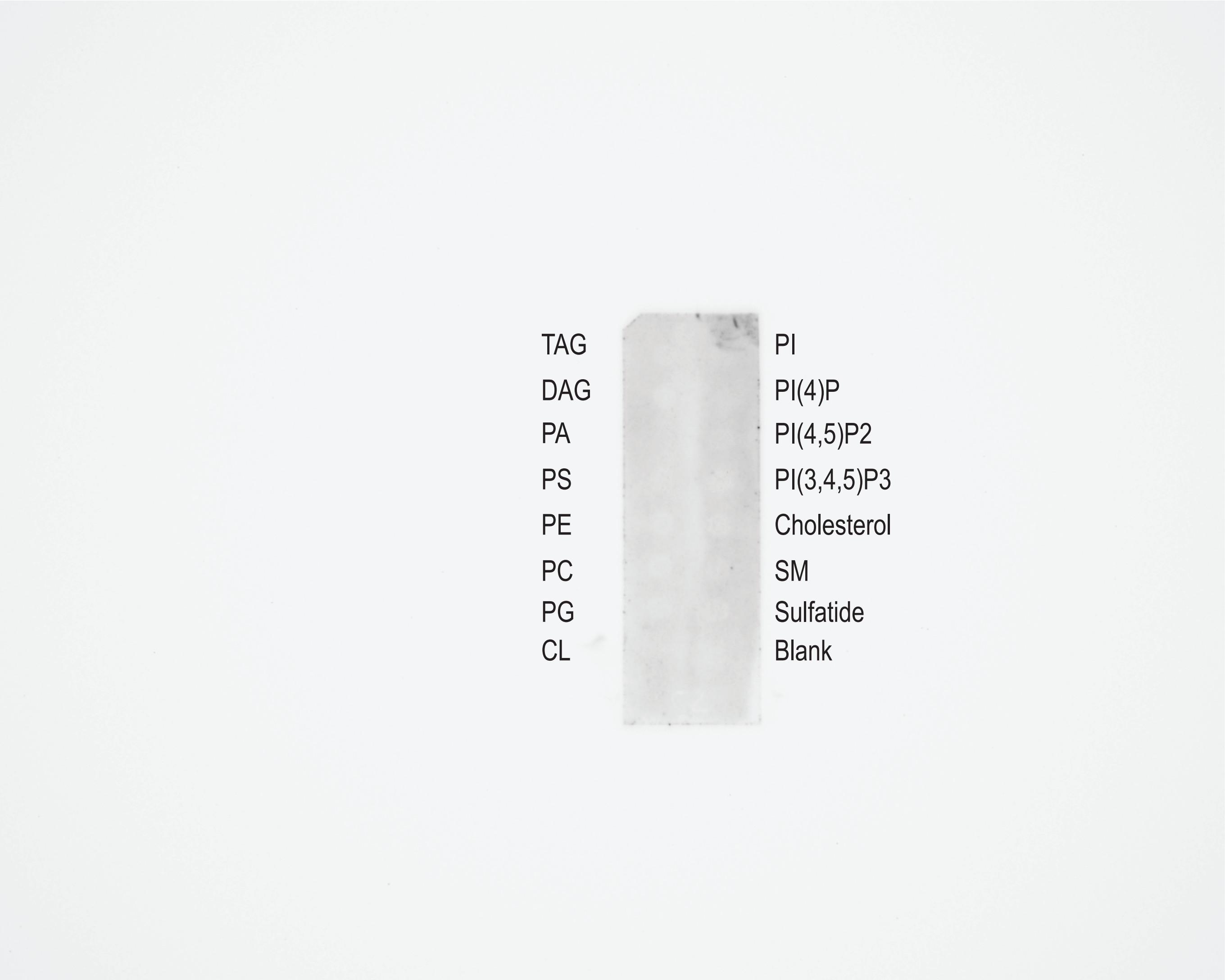

Supplement: Figure 1—source data 3. [file elife-105541-fig1-data3.zip › Figure 1-Source Data 3/Figure 1F-Source Data 9.pdf]
