# Supplementary material for: The RAB27A effector SYTL5 regulates mitophagy and mitochondrial metabolism
Source: eLife. 2025 Nov 26;14:RP105541. doi: 10.7554/eLife.105541 (PMC12656530; doi:10.7554/eLife.105541)
Supplement: Supplementary file 1. [file elife-105541-supp1.docx]

**SYTL5 interactome**

Proteins co-immunoprecipitated with SYTL5-EGFP vs EGFP expressing control.

| **Gene ID** | **logFC** | **P.Value** | **Protein ID** |
| --- | --- | --- | --- |
| SYTL5 | 8,190967 | 6,18E-06 | Q8TDW5 |
| RAB27A | 4,904658 | 0,00013 | P51159; O00194 |
| DICER1 | 4,072889 | 3,36E-05 | Q9UPY3 |
| NRBP1 | 3,616261 | 0,000139 | Q9UHY1 |
| TUBGCP3 | 3,480067 | 2,23E-06 | Q96CW5 |
| UBXN4 | 3,364929 | 0,000445 | Q92575 |
| PPME1 | 2,860594 | 0,003682 | Q9Y570 |
| SUGT1 | 2,644558 | 3,96E-05 | Q9Y2Z0 |
| RRM2 | 2,58741 | 0,000772 | P31350 |
| S100A16 | 2,489595 | 0,019199 | Q96FQ6 |
| RBX1 | 2,283705 | 5,54E-05 | P62877 |
| CAPNS1 | 2,245698 | 0,0045 | P04632 |
| IPO5 | 2,152529 | 0,001141 | O00410 |
| SQSTM1 | 2,087749 | 0,019914 | Q13501 |
| CDK5RAP1 | 2,083531 | 0,022579 | Q96SZ6 |
| PSMD9 | 2,066147 | 0,000637 | O00233 |
| ANXA1 | 2,015019 | 0,008846 | P04083 |
| RIPK2 | 1,998221 | 0,012785 | O43353 |
| TUBB2A | 1,937802 | 0,032975 | Q13885 |
| FBXL18 | 1,932709 | 0,000593 | Q96ME1 |
| ACACA | 1,884369 | 0,00271 | Q13085; O00763 |
| TRAF2 | 1,879215 | 0,041615 | Q12933 |
| ATXN10 | 1,807462 | 0,001441 | Q9UBB4 |
| PFKFB3 | 1,806392 | 0,011617 | Q16875 |
| CASC5 | 1,787295 | 0,038936 | Q8NG31 |
| ACTR2 | 1,753581 | 0,00102 | P61160 |
| PDCD6IP | 1,750369 | 0,001188 | Q8WUM4 |
| DNAJB2 | 1,746104 | 0,020493 | P25686 |
| MTAP | 1,723933 | 0,037343 | Q13126 |
| NOSIP | 1,722576 | 0,041459 | Q9Y314 |
| LARP4 | 1,673434 | 0,010333 | Q71RC2 |
| ENY2 | 1,672969 | 0,029441 | Q9NPA8 |
| ARMCX3 | 1,661646 | 0,020894 | Q9UH62 |
| TIMM13 | 1,652287 | 0,016672 | Q9Y5L4 |
| ZC3HAV1L | 1,646732 | 0,000559 | Q96H79 |
| CLU | 1,641816 | 0,018032 | P10909 |
| STAT3 | 1,630662 | 0,017936 | P40763 |
| MMP2 | 1,596861 | 0,01583 | P08253 |
| ARHGAP28 | 1,594874 | 0,029771 | Q9P2N2 |
| CASP2 | 1,54764 | 0,006055 | P42575 |
| NECAP1 | 1,532475 | 0,001098 | Q8NC96; Q9NVZ3 |
| CHORDC1 | 1,530842 | 0,006654 | Q9UHD1 |
| AHNAK2 | 1,518123 | 0,003188 | Q8IVF2 |
| P4HA2 | 1,507659 | 0,01657 | O15460 |
| SLAIN2 | 1,504025 | 0,038526 | Q9P270 |
| TTLL12 | 1,496256 | 0,016402 | Q14166 |
| CIAO1 | 1,47458 | 0,002001 | O76071 |
| PSMD13 | 1,469132 | 0,004425 | Q9UNM6 |
| PAPSS2 | 1,468267 | 0,024743 | O95340 |
| PKP3 | 1,462143 | 0,000241 | Q9Y446 |
| MAPKAPK3 | 1,43686 | 0,036615 | Q16644 |
| LAMC2 | 1,424905 | 0,014389 | Q13753 |
| TMED10 | 1,420889 | 0,033461 | P49755 |
| GGA2 | 1,401072 | 0,036446 | Q9UJY4 |
| SMYD5 | 1,380067 | 0,000962 | Q6GMV2 |
| MYD88 | 1,379573 | 0,0036 | Q99836 |
| PTPN23 | 1,372845 | 0,042911 | Q9H3S7 |
| PRMT3 | 1,367264 | 0,035439 | O60678 |
| SRR | 1,335156 | 0,018756 | Q9GZT4 |
| METTL2B | 1,318423 | 0,002052 | Q6P1Q9 |
| CEP55 | 1,303196 | 0,042401 | Q53EZ4 |
| KNTC1 | 1,293798 | 0,013151 | P50748 |
| MMADHC | 1,287966 | 0,007304 | Q9H3L0 |
| RPS6KA4 | 1,278372 | 0,026028 | O75676 |
| TCEAL4 | 1,277767 | 0,041367 | Q96EI5; Q9H3H9 |
| KRT18 | 1,273972 | 0,000277 | P05783 |
| EIF3D | 1,249965 | 0,001692 | O15371 |
| MAP3K6 | 1,235349 | 0,038163 | O95382 |
| FAM83D | 1,235204 | 0,001544 | Q9H4H8 |
| FLCN | 1,223376 | 0,025555 | Q8NFG4 |
| NARFL | 1,207308 | 0,005387 | Q9H6Q4; Q9UHQ1 |
| TK1 | 1,191836 | 0,022913 | P04183 |
| TSN | 1,182404 | 0,012781 | Q15631 |
| MOGS | 1,181984 | 0,013095 | Q13724 |
| TRIP13 | 1,179669 | 0,007178 | Q15645 |
| SH3GL1 | 1,148087 | 0,001254 | Q99961; Q99962 |
| TGFB1 | 1,146935 | 0,009311 | P01137 |
| TBK1 | 1,146181 | 0,005314 | Q9UHD2 |
| UBAP2L | 1,131475 | 0,034553 | Q14157 |
| HMMR | 1,124677 | 0,038667 | O75330 |
| MAP2K4 | 1,123533 | 0,004105 | P45985 |
| LRRC40 | 1,116807 | 0,03566 | Q9H9A6 |
| TAB2 | 1,114102 | 0,012624 | Q9NYJ8 |
| IPO7 | 1,106622 | 0,002335 | O95373 |
| DECR1 | 1,096331 | 0,014198 | Q16698 |
| PRKAR1A | 1,090646 | 0,049639 | P10644 |
| ATP6V1A | 1,082598 | 0,006145 | P38606 |
| ATG4B | 1,062937 | 0,01514 | Q9Y4P1 |
| CIAPIN1 | 1,056349 | 0,026294 | Q6FI81 |
| STIM1 | 1,036432 | 0,036684 | Q13586 |
| TSNAX | 1,033898 | 0,012445 | Q99598 |
| PDLIM2 | 1,014087 | 0,008808 | Q96JY6 |
| KIF11 | 1,012826 | 0,007811 | P52732 |
| NCAPD2 | 1,005509 | 0,004454 | Q15021 |
| FLYWCH2 | 1,002772 | 0,036116 | Q96CP2 |
| FLNC | 0,997402 | 0,031493 | Q14315 |
| ZCCHC11 | 0,995037 | 0,011036 | Q5TAX3 |
| ARF5 | 0,98277 | 0,002189 | P84085; P61204; P18085; P84077 |
| ARHGAP29 | 0,982689 | 0,020558 | Q52LW3 |
| INPP4A | 0,977505 | 0,046529 | Q96PE3 |
| IKBKG | 0,964638 | 0,017171 | Q9Y6K9 |
| CACYBP | 0,96422 | 0,007507 | Q9HB71 |
| SEC23B | 0,951773 | 0,047922 | Q15437 |
| CARHSP1 | 0,950642 | 0,016008 | Q9Y2V2 |
| STRN | 0,93495 | 0,004936 | O43815 |
| KIAA0930 | 0,920553 | 0,044996 | Q6ICG6 |
| DPYSL3 | 0,910877 | 0,006555 | Q14195 |
| ACAA1 | 0,902484 | 0,014043 | P09110 |
| PPIA | 0,901801 | 0,005015 | P62937 |
| JAK1 | 0,898744 | 0,029572 | P23458 |
| PXN | 0,89135 | 0,032021 | P49023 |
| RIPK1 | 0,880408 | 0,02096 | Q13546 |
| HACL1 | 0,873144 | 0,005027 | Q9UJ83 |
| C16orf13 | 0,872785 | 0,043785 | Q96S19 |
| STAU2 | 0,871501 | 0,007129 | Q9NUL3 |
| CTTN | 0,868965 | 0,002283 | Q14247 |
| NEDD1 | 0,865712 | 0,00298 | Q8NHV4 |
| RELA | 0,848508 | 0,008652 | Q04206 |
| MOCS2 | 0,844002 | 0,02455 | O96007 |
| SESTD1 | 0,843323 | 0,022617 | Q86VW0 |
| CNN2 | 0,84227 | 0,005121 | Q99439 |
| TNS3 | 0,824331 | 0,019403 | Q68CZ2; Q63HR2 |
| MYH9 | 0,820463 | 0,020254 | P35579; P35749 |
| FAM120B | 0,81995 | 0,012614 | Q96EK7 |
| PHLDB1 | 0,816857 | 0,002876 | Q86UU1 |
| LAMB3 | 0,811098 | 0,007705 | Q13751 |
| ERCC6L | 0,809206 | 0,022191 | Q2NKX8 |
| RASA4 | 0,79333 | 0,044026 | O43374; C9J798 |
| SH3KBP1 | 0,78816 | 0,014872 | Q96B97 |
| ACTB | 0,783173 | 0,00407 | P60709; Q6S8J3; A5A3E0; P0CG38; Q562R1; P0CG39; Q9BYX7 |
| CNN3 | 0,781781 | 0,003728 | Q15417 |
| DNMBP | 0,770568 | 0,030522 | Q6XZF7 |
| INF2 | 0,769802 | 0,005056 | Q27J81 |
| NFKB2 | 0,762598 | 0,02469 | Q00653 |
| DOK1 | 0,758782 | 0,022414 | Q99704 |
| MVP | 0,758137 | 0,025696 | Q14764 |
| SEPT9 | 0,750806 | 0,015745 | Q9UHD8 |
| SFN | 0,747282 | 0,038485 | P31947 |
| UFSP2 | 0,736462 | 0,032911 | Q9NUQ7 |
| GNA13 | 0,729323 | 0,01046 | Q14344 |
| VPS37B | 0,721343 | 0,006336 | Q9H9H4 |
| MCMBP | 0,716545 | 0,008801 | Q9BTE3 |
| DPYSL2 | 0,707331 | 0,02086 | Q16555 |
| RNPEP | 0,705101 | 0,032267 | Q9H4A4 |
| UBA6 | 0,691729 | 0,015468 | A0AVT1 |
| GSTK1 | 0,687332 | 0,046851 | Q9Y2Q3 |
| GMPS | 0,683068 | 0,014906 | P49915 |
| POLR2B | 0,676992 | 0,027717 | P30876 |
| DBNL | 0,675429 | 0,006534 | Q9UJU6 |
| PLCG1 | 0,669824 | 0,049137 | P19174 |
| HSPA4 | 0,665847 | 0,027702 | P34932 |
| LIMD1 | 0,661786 | 0,018739 | Q9UGP4 |
| CAT | 0,653596 | 0,036557 | P04040 |
| ANXA2 | 0,652983 | 0,016136 | P07355; A6NMY6 |
| CBR1 | 0,64346 | 0,032038 | P16152; O75828 |
| PRDX2 | 0,643131 | 0,022495 | P32119 |
| UBA1 | 0,634137 | 0,022102 | P22314 |
| NCAPG | 0,632896 | 0,021943 | Q9BPX3 |
| MAT2A | 0,622619 | 0,013543 | P31153; Q00266 |
| GRB2 | 0,619474 | 0,025687 | P62993 |
| KIF1B | 0,612282 | 0,046537 | O60333; Q12756; O43896 |
| KIF5B | 0,611182 | 0,018915 | P33176; O60282; Q12840 |
| MAT2B | 0,602416 | 0,016771 | Q9NZL9 |
